# Sustainable Diepoxide Monomers for Polyoxazolidinone‐Based High‐Performance Thermoplastics

**DOI:** 10.1002/cssc.71140

**Published:** 2026-10-10

**Authors:** Lisa Van Emelen, Friederike Ratsch, Patrick Klein, Sarah Brettschneider, Hans Gildenast, Markus D. Schötz, Mathias Glaßner, Martin Reiher, Elena Dirksen, Felix Niebisch, Kiruthika Periasamy, Ria Ghosh, Sohajl Movahhed, Kai Laemmerhold, Jürgen Klankermayer

**Affiliations:** ^1^ CAT Catalytic Center Institut für Technische und Makromolekulare Chemie RWTH Aachen Aachen Germany; ^2^ Covestro Deutschland AG Leverkusen Germany

**Keywords:** diglycidyl ether, high performance thermoplastics, polymers, polyoxazolidinone, renewable resources

## Abstract

Owing to their outstanding mechanical, thermal, and chemical properties, polyoxazolidinones, which are accessible from the reaction of diepoxides and diisocyanates, have recently gained interest as a new class of high‐performance thermoplastic materials. However, mainly bisphenol A diglycidyl ether (BADGE) has thus far served as benchmark for the diepoxide part. Herein, ten bisphenol‐free diepoxides that are either bio‐based or derived from chemical recycling are presented. All these epoxides selectively resulted in linear polyoxazolidinones using methylene diphenyl diisocyanate (MDI) and *p*‐tert‐butyl phenyl glycidyl ether as chain regulators. The new polymers were characterized, and the thermal and mechanical properties were compared to the BADGE‐based reference polyoxazolidinone. With three of the new diepoxide monomers, polyoxazolidinones with properties matching or surpassing those of BADGE‐based polyoxazolidinone were obtained.

## Graphical abstract

Sustainable diglycidyl ethers could replace bisphenol A diglycidyl ether (BADGE) as a building block for polyoxazolidinones, yielding high‐performance thermoplastics with similar thermal and mechanical properties.

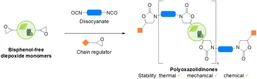

## 1 Introduction

High‐performance thermoplastic materials (HPTs) are polymers that preserve usable properties at elevated temperatures, even after long‐term exposure to solvents or other chemicals, air, mechanical impact, electricity, or radiation [1, 2, 3]. Polymers with such advantageous properties, like high glass‐transition temperatures (*T*_g_), outstanding mechanical properties, and chemical inertness, find application in domestic use in the automotive, electronics, and aerospace industries [1, 2, 3]. However, a drawback of the majority of commonly used HPTs is their tedious, often multistep synthesis from complex monomers, which leads to a significant environmental impact and a higher carbon footprint than commodity plastics [3]. Therefore, the development of HPTs that are accessible through an environmentally benign route is highly desirable. Polyoxazolidinones are accessible via the polyaddition of diepoxides and diisocyanates (Figure 1). As chain terminators, a monofunctional epoxide can be used. While the formation of 1,2‐oxazolidin‐2‐ones from epoxides and isocyanates was reported as early as 1958 [4] and the first characterized polyoxazolidinones were described in 1970 [5, 6], the full potential of this polymer class could only be explored after optimization of the synthesis. Through the development of a tailored synthetic approach, polyoxazolidinones from methylenediphenyl diisocyanate (MDI) and bisphenol A diglycidyl ether (BADGE) are accessible in a highly selective one‐step process [7]; a solvent‐free alternative route for large‐scale production is currently being investigated [8, 9, 10]. In this approach, tetraphenyl phosphonium salt catalysts ([PPh_4_]Cl or [PPh_4_]Br) were employed at elevated temperature to suppress isocyanurate (trimer) formation and selectively yield linear polymers containing 5‐oxazolidinone moieties [7, 10, 11].

**FIGURE 1:**
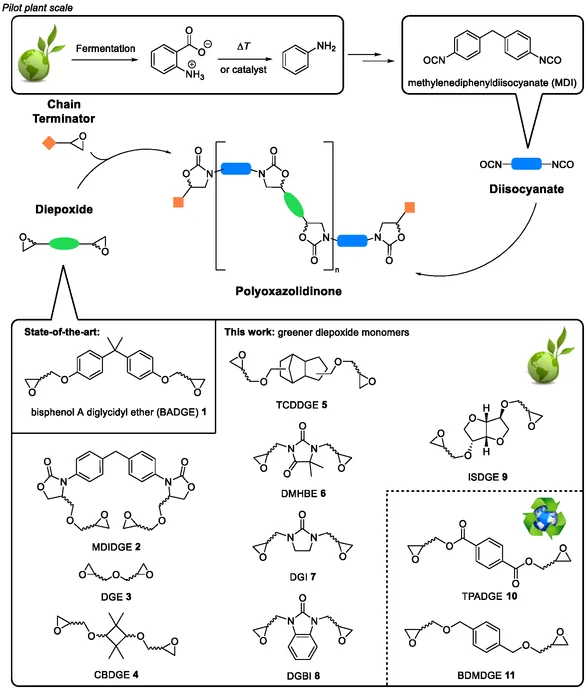
General reaction scheme for the synthesis of polyoxazolidinones and monomers investigated in this work. BDMDGE = 1,4‐benzenedimethanol diglycidyl ether; CBDGE = 2,2,4,4‐tetramethyl‐1,3‐cyclobutanediol diglycidyl ether; DGBI = diglycidyl benzimidazolidinone (1,3‐bis(oxiran‐2‐ylmethyl)‐1,3‐dihydro‐2*H*‐benzo[*d*]imidazol‐2‐one); DGE = diglycidyl ether; DGI = diglycidyl imidazolidinone; DMHBE = 5,5‐dimethyl hydantoin bisepoxide; ISDGE = isosorbide diglycidyl ether; MDIDGE = 4,4′‐methylene diphenyl diisocyanate diol diglycidyl ether; TCDDGE = tricyclodecanedimethanol diglycidyl ether; TPADGE = terephthalic acid diglycidyl ether.

The developed process yields polyoxazolidinone HPT with excellent, adaptable properties comparable to other high‐performance thermoplastics [11]. Furthermore, according to a life cycle analysis, the one‐step process could reduce greenhouse gas emissions by 35–45 % compared to other HPTs (calculated for fossil‐based production) [3. A shift to renewable energy sources and biobased monomers [12] could lead to even further reduction of greenhouse gas emissions [3]. Also, chemical recycling of existing polymers to new monomers, as well as carbon capture and utilization, can contribute to rendering plastics carbon neutral and reducing the energy demands and operational expenses regarding fossil‐based products [13].

Both for the diisocyanate and diepoxide monomers, renewable building blocks are envisaged. For MDI, which is derived from aniline, a biobased route relying on fermentation of sugars to anthranilic acid and subsequent decarboxylation is currently tested on a pilot‐plant scale at Covestro [14, 15, 16, 17, 18, 19]. For the diepoxide, on the other hand, our current benchmark monomer is bisphenol A diglycidyl ether (BADGE) or similar bisphenol‐based diepoxides [11]. Not only are these building blocks mainly fossil‐based, Bisphenol A is also classified as a substance of very high concern (SVHC) in the EU. This may lead to further restrictions of use in the future in addition to the prohibition to use BPA‐based materials in food contact, which entered into force in early 2025 (with transition periods) [20, 21, 22]. Other diepoxide monomers for the synthesis of polyoxazolidinones are mentioned in the literature but lack a detailed study of the thermal and mechanical properties of the resulting polymers [23, 24].

Herein, 10 alternative diepoxides, which are either biobased or obtained as polymer recycling products, were used as monomers for polyoxazolidinones, and the materials were fully thermally and mechanically characterized [25, 26]. Several of the new polyoxazolidinones exhibited comparable or better properties than the BADGE‐derived polymers.

## 2 Experimental Section

### 2.1 Monomer Synthesis

The new diepoxide monomers were either synthesized from the corresponding diols or diamides or purchased if commercially available and purified if necessary (see Supporting Information).

### 2.2 Polymer Synthesis

The new monomers were then used in the drop‐in protocol that was previously optimized for BADGE using 4,4′‐MDI as diisocyanate, *para*‐*tert*‐butylphenyl glycidyl ether (*p*‐*t*BPGE) as monofunctional endcap, and [PPh_4_]Cl as catalyst in PhCN at 175–185 °C [25]. *p*‐*t*BPGE was selected as chain regulator because its CH_3_ protons allow easy determination of the molecular weight via end‐group analysis in ^1^H‐NMR, but it could potentially be replaced by other (bio‐based) aromatic glycidyl ethers, like furfuryl glycidyl ether. Under a continuous flow of Ar, a Schlenk glass flask A was charged with catalyst and benzonitrile and heated to reaction temperature (internal temperature). An overview of the exact reaction conditions is given in Table 1. A second Schlenk flask B was charged under a continuous flow of Ar with 4,4′‐MDI, the diepoxide, a first portion of the end‐capping agent (*p*‐*t*BPGE), and benzonitrile. The monomer solution in flask B was added to the catalyst solution in flask A over 90 min and further stirred for 30 min before the addition of the second portion of *p*‐*t*BPGE to terminate any reactive chain ends. For the DGI‐based polymer, N‐methylpyrrolidone (NMP) was used as solvent instead. The dropwise addition ensured a controlled reaction without MDI accumulation to suppress trimer formation (as monitored by FTIR measurements). After the addition, the reaction was stirred at 175–185 °C for another 180 min. Isocyanate conversion was followed by FTIR measurements, showing a gradual disappearance of the isocyanate band at 2260 cm^−1^. Subsequently, NMP (80 mL) was added to dilute the reaction mixture, and the viscous solution was allowed to cool down to 120 °C (for some polymers, the addition of NMP was omitted—see Table 1). The precipitation of the polymer was performed in ethanol or ethyl acetate (DGE‐based polymer) at ambient temperature by slowly adding the reaction mixture to a large volume of ethanol under vigorous shear force mixing (Ultra‐Turrax, IKA). The product was isolated by filtrationand washed with ethanol, water, and ethanol again, and dried at ambient temperature overnight. In some cases (see Table 1), a reprecipitation step in ethanol to remove residual monomer and/or solvent was performed after redissolving the polymer in NMP under a continuous Ar flow at 80–95 °C. Precipitation in ethanol, washing, and filtration were performed at room temperature using the same procedure as in the first precipitation step. After drying at ambient temperature overnight, the polymer was further dried under vacuum (50 mbar) at 40 °C for 2 h and at 100 °C for 12 h. The polymer prepared from MDIDGE was additionally dried at 140 °C for 4 h, while the material prepared from DMHBE was dried at 100 °C for 20 h, at 150 °C for 2 h, and at 170 °C for 2.5 h. Colourless to off‐white polyoxazolidinone powders were obtained.

**TABLE 1:** Detailed reaction conditions of polyoxazolidinone syntheses with various renewable diepoxide monomers.^a^

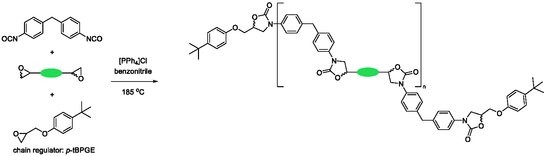							
Diepoxide, equiv.	Target *n*	MDI, g	*p*‐*t*BPGE, equiv.	[PPh_4_]Cl, mol%	Benzonitrile [mL] in flask A + B	NMP [mL] added after 300 min	Redissolved in NMP
BADGE (0.96)	25	29.50	0.08 + 0.20	2	92.1 + 79.2 + 9.9^b^	—	No
MDIDGE^c^ (0.98)	50	20.05	0.04 + 0.10	2	69.8 + 107.3	100	Yes
DGE (0.995)^d^	200	20.56	0.00995 + 0.10	1	92.1 + 46	50^e^	No
CBDGE (0.98)	50	32.00	0.04 + 0.02	2	74.2 + 74.2	80	Yes
TCDDGE (0.98)	50	30.00	0.04 + 0.10	2	76.7 + 76.7	60	Yes
ISDGE (0.97)	30	26.00	0.06 + 0.075	2	61.0 + 61.0	—	Yes
TPADGE (0.96)	25	2.00	0.08 + 0.10	2	3.0 + 7.0	—	Yes
BDMDGE (0.96)	25	25.00	0.08 + 0.20^f^	2	77.9 + 84.8	—	No
DMHBE (0.98)	60	33.00	0.03 + 0.10	2	73.8 + 73.8	100	Yes
DGI (0.99)^g^	100	4.01	0.02 + 0.10	1	10.6 + 10.6	10^e^	No
DGBI (0.99)	70	1.30	0.03 + 0.10	2	3.0 + 3.0	5	No
DGBI (0.98)	50	1.50	0.04 + 0.10	2	3.4 + 3.4	—	No

a Reaction under Ar, stirring at 260 rpm, addition of monomer mixture (diepoxide + MDI + 1st fraction of *p*‐*t*BPGE) over 90 min, 30 min stirring, addition of 2nd portion *p*‐*t*BPGE, 180 min stirring.

b 9.9 mL mixed with the second portion of chain regulator.

c MDIDGE was first dissolved in benzonitrile under heating to 80 °C, after which the mixture was cooled to rt and MDI and the 1st fraction of *p*‐*t*BPGE were added.

d Reaction at 175 °C.

e Benzonitrile added instead of NMP.

f Added with 12.7 mL benzonitrile.

g 180 °C, 3 h, reaction in NMP.

## 3 Results and Discussion

### 3.1 Monomer Synthesis

The diepoxide monomers were synthesized from the corresponding diol, either through direct glycidylation (**2, 4, 5, 7, 8, 11** from Figure 1) with epichlorohydrin [27] or via (di)allylation with allyl bromide and subsequent epoxidation in 42–89 % yield (Table 2) [28]. In the direct glycidylation route, self‐condensation of epichlorohydrin to yield a tetramer [29] (Figure 2) was observed in some instances when the diol was more sterically hindered and therefore less reactive (ISDGE) [30] or when less reactive urea starting materials were employed (DGI, DGBI). In the synthesis of DGI and DGBI, this side reaction could be suppressed by using a smaller excess of base. For ISDGE and DGE, the allylation route was preferred.

**TABLE 2:** Synthesis of starting monomers.

Diepoxide	Method	Yield, %
MDIDGE	Diglycidylation	49
DGE	Allylation–epoxidation	43
CBDGE	Diglycidylation	89
TCDDGE	Diglycidylation	65
ISDGE	Diallylation–epoxidation	67
BDMDGE	Diglycidylation	74
DGI	Diglycidylation	70
DGBI	Diglycidylation	52

**FIGURE 2:**
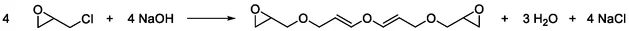
Tetramerization of epichlorohydrin.

Both epichlorohydrin and allyl bromide can be prepared from glycerol [31]. For epichlorohydrin, this is already performed at an industrial scale: chlorination of glycerol with HCl in the presence of an acidic catalyst results in monopropanediols and dichloropropanol species [32, 33, 34]. The latter then undergo elimination of HCl in the presence of a base to yield epichlorohydrin, while the former are recycled to the first step [35]. On the other hand, deoxydehydration of glycerol yields allyl alcohol [36, 37], which under acidic conditions can be converted into allyl bromide using HBr [38]. For allyl alcohol, also biobased sources via propylene glycol from sugars have been reported [39].

The MDI‐based diol (obtained from Covestro) was successfully diglycidylated to give MDIDGE (MDI‐diol diglycidyl ether, **2**) in 49 % yield. The access to the diepoxide from MDI is possible in three steps, with all reactants being available from biobased sources [31]. The corresponding polyoxazolidinone could be potentially biobased as both of its monomers are MDI‐based and thus accessible from biobased aniline [14, 15, 16, 17, 18, 19], which is already available on an industrial scale [18].

DGE (diglycidyl ether, 2,2′‐(oxybis(methylene))bisoxirane, **3**) leads to a very similar polymer as MDIDGE, yet as preparation of the MDI‐based diol is not necessary, its synthesis requires fewer steps than MDIDGE, but a possible drawback, however, is its toxicity. Epoxidation of allyl glycidyl ether with *meta*‐chloroperbenzoic acid (1.4 equiv.) resulted in 43 % diglycidyl ether [28, 39, 40]. Polyoxometalate‐catalyzed epoxidation with H_2_O_2_ as a more atom‐efficient oxidant has been reported as well [41]. Allyl glycidyl ether can be prepared from allyl alcohol and epichlorohydrin [42].

CBDGE (cyclobutanediol diglycidylether **4**) was synthesized from 2,2,4,4‐tetramethylcyclobutanediol, which is already broadly investigated as a replacement for bisphenol A in polyesters [43] and used, for example, by Eastman as a component of the bisphenol A‐free Tritan copolyester. 2,2,4,4‐tetramethylcyclobutanediol in its turn, can be prepared from isobutyraldehyde, which is a byproduct of propene hydroformylation, but biobased access could be envisaged from CO_2_ [44]. Oxidation of the aldehyde to isobutyric acid and subsequent pyrolysis, followed by reduction of the diketone intermediate, produces the diol in high yield [45]. In spite of the rather sterically hindered alcohol groups, diglycidylation was successfully performed in up to 89 % yield, and the corresponding polyoxazolidinone was prepared.

Tricyclodecane dimethanol (TCD) is traditionally obtained by hydroformylation of dicyclopentadiene available from fossil sources [46], but recently, Li and co‐workers reported a sustainable pathway to dicyclopentadiene based on biobased furfuryl alcohol [47]. The diepoxide (TCDDGE, tricyclodecane dimethanol diglycidyl ether, **5**) was obtained via diglycidylation of TCD with epichlorohydrin in an aqueous NaOH solution in 65 % yield.

Isosorbide diglycidyl ether (ISDGE, **9**) has been used as a sugar‐derived, nontoxic, rather rigid, and chiral building block for epoxy resins [12, 48, 49, 50]. Due to the steric hindrance, especially on the endo‐positioned 5‐OH group [30], direct diglycidylation of isosorbide with epichlorohydrin or epibromohydrin resulted in low yields (37–42 %) and considerable oligomerization of the epihalohydrin (Figure 2) [29, 51]. Diallylation with allyl bromide and subsequent epoxidation with oxone [12, 52, 53] produced ISDGE in 67 % overall yield and high purity (98.7 %).

TPADGE (terephthalic acid diglycidyl ether, **10**) and its reduced form BDMDGE (benzenedimethanol diglycidyl ether, **11**) can be obtained from chemical recycling of PET (polyethylene terephthalate), which is a well‐established process [54]. TPADGE is commercially available as Denacol‐Ex 711, whereas BDMDGE was obtained by diglycidylation of 1,4‐benzenedimethanol in 74 % yield. 1,4‐benzenedimethanol could be either obtained by reduction of dimethyl terephthalate (PET methanolysis product) [55] or directly via PET hydrogenolysis [56, 57, 58].

For DGI (diglycidyl imidazolidinone, **7**) and DGBI (diglycidyl benzimidazolidinone, **8**), diglycidylation with epichlorohydrin of the corresponding imidazolidinone was performed in moderate yield (70 and 52 % yield, respectively) in DMSO. The imidazolidinones are cyclic ureas, which can be prepared from the corresponding diamines and CO_2_ [59]. For ethylene diamine [60, 61, 62] biobased routes starting from carbohydrates [62] have already been disclosed, while *ortho*‐phenylenediamine [63] in principle could be prepared from aniline, which could be produced renewably as mentioned above [14, 15, 16, 17, 18, 19]. Lastly, 5,5‐dimethyl hydantoin bisepoxide (DMHBE, **6**) is commercially available and can be synthesized via the Bucherer‐Bergs reaction starting from inexpensive base chemicals (acetone, potassium cyanide, and ammonium carbonate) [64, 65, 66, 67].

### 3.2 Polymers

#### 3.2.1 Chain Length and Molecular Weight

The polyoxazolidinone polymers were prepared from the corresponding diepoxides and 4,4′‐MDI using 1–2 mol% [PPh_4_]Cl as catalyst and benzonitrile as solvent. According to the modified Carother’s equation [68], the obtained chain length is controlled by the molar ratio of diepoxide, MDI and, end cap as well as the conversion. The monofunctional end cap *p*‐*t*BPGE not only helps control the chain growth, but the excess of this epoxide at the end of the reaction also converts any isocyanurate moieties formed into oxazolidinone groups [69], which improves the thermoplastic properties of the material and lowers the polydispersity. The polymers were precipitated in ethanol or ethyl acetate to remove unreacted monomers and short oligomers as well as the reaction solvent. In the FTIR spectrum, a strong absorption band around 1750 cm^−1^ indicated the formation of oxazolidinone moieties, whereas the disappearance of the band at *ca*. 2260 cm^−1^ pointed at full conversion of the isocyanate. For assigned NMR spectra of the polymers, we refer to the Supporting Information.

The BADGE‐based polyoxazolidinone reference material exhibited a number average molecular weight (*M*_*n*_) of 11.7 kg/mol. In Figure S62, the obtained chain lengths (based on end‐group analysis in NMR) *versus* the targeted values are depicted, whereas the properties of all synthesized polymers are listed in Table 3. As expected, the higher the targeted chain length, the more the obtained chain length (as measured by ^1^H‐NMR) deviates from the target as the exact stoichiometric ratio and purity of the reactants strongly impact the chain length that can be obtained. This is well illustrated by the MDIDGE‐ and DGE‐based polymers. In the MDIDGE‐based polymer, 4‐ and 5‐oxazolidinone moieties in principle alternate in an AA–BB fashion, whereas in the DGE‐derived polymer, almost exclusively 5‐oxazolidinone units are present. To achieve a polymer of about the same molecular weight, the number of repeating units (*n*) in the DGE‐based material should be twice (target *n* = 100) as high as for the MDIDGE‐based polymer (target *n* = 50). However, it can be seen that only with a targeted *n* = 200, the molecular weight of the DGE‐based polymer marginally surpassed that of the MDIDGE‐derived compound. With CDDGE, TCDDGE, DMHBE, and ISDGE, the obtained chain length was close to the targeted value. With TPADGE, an insoluble residue precipitated during the reaction, and afterwards, a poorly soluble polymer was obtained after the reaction so that GPC and NMR measurements were not possible. For this reason, and due to the high brittleness of the material, this polymer was not further studied despite its high glass‐transition temperature (*T*_g_ = 178 °C). The presence of crosslinking isocyanurate moieties in the polymer might explain its low solubility and brittleness, but the FTIR spectrum remains inconclusive since the C═O and C–O vibrations around 1730 and 1410 cm^−1^ of the terephthalate ester moieties [70] appear in the same region as those of the isocyanurate group.‍ For BDMDGE, it was not clear why the chain length obtained considerably surpassed the targeted value. It must be noted, however, that end‐group analysis becomes less accurate at higher *M*_*n*_ (above *ca*. 10^4^ kg/mol), which could partially explain this observation. For DGI, the synthesis was conducted in NMP due to the limited solubility of the polymer in benzonitrile, which prevented the formation of long polymer chains. *Ortho*‐dichlorobenzene or 1,2,4‐trichlorobenzene in combination with sulfolane did not improve the polymerization, yet, in NMP, an average chain length of about 50 repeating units could be achieved. Polymerization of DGBI, which is more soluble in benzonitrile, resulted in relatively short chains (*n* = 20) compared to the target (*n* = 70).

**TABLE 3:** Molecular weight, thermal and mechanical analysis of the synthesized polyoxazolidinones.

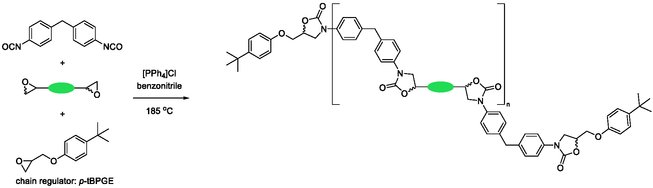							
Diepoxide	*n*	*M*_*n*_ from NMR^a^, kg/mol	*M*_*n*_ from GPC, kg/mol	*M*_w_ from GPC, kg/‍mol	*T*_g_, °C	*T*_d_, °C	Δ*w*% after 1 h at 300 °C	*E*‐‍modulus, GPa	Tensilestrength, MPa	Average elongation at break, %	Maximal elongation at break, %	Microhardness, 10^**2 **^N/mm^2^
BADGE	19	11.7	8.4	27.8	170	365	n.d.	1.55	80 ± 1	80 ± 11	93.1	1.80 ± 0.11
MDIDGE	34	26.5	9.8	43.2	167	374	2.8	1.67 ± 0.05	77 ± 1	51 ± 21	81.8	1.74 ± 0.18
DGE	94	36.4	14.6	40.0	168	377	<0.5	1.81 ± 0.10	77 ± 1	40 ± 12	51.2	1.54 ± 0.16
CBDGE	34	17.9	12.7	27.3	146	419	1.2	1.38 ± 0.08	58 ± 1	51 ± 22	83.0	1.09 ± 0.07
TCDDGE	43	24.7	16.0	31.6	124	422	0.8	1.40 ± 0.11	53 ± 4	4.3 ± 0.4	4.6	1.55 ± 0.07
ISDGE	25	13.4	7.6	24.1	151	390	1.5	1.68 ± 0.06	69 ± 1	56 ± 29	95.8	1.23 ± 0.20
TPADGE	n.d.^b^	n.d.^b^	n.d.^b^	n.d.^b^	178	324	24.9	n.d.	n.d.	n.d.	n.d.	n.d.
BDMDGE	79	40.2	15.4	49.7	126	388	n.d.	1.32 ± 0.06	66 ± 1	60 ± 25	85.4	1.40 ± 0.17
DMHBE	52	26.2	8.8	38.0	204	380	3.4	1.82 ± 0.04	85 ± 1	45 ± 9	55.7	1.76 ± 0.27
DGI	50	23.1	3.10	5.0	172	379	<0.5	n.d.	n.d.	n.d.	n.d.	n.d.
DGBI	20	10.6	13.3	28.0	213	367	<0.5	n.d.	n.d.	n.d.	n.d.	n.d.

a From ^1^H‐NMR via end group analysis.

b Sample not soluble in DMAc or DMSO due to high amount of isocyanurate trimers.

GPC measurements suggested a monomodal molecular weight distribution for all synthesized polymers (Figure 3), but as each material expands differently in the eluent, a comparison of the GPC molecular weights is not useful.

**FIGURE 3:**
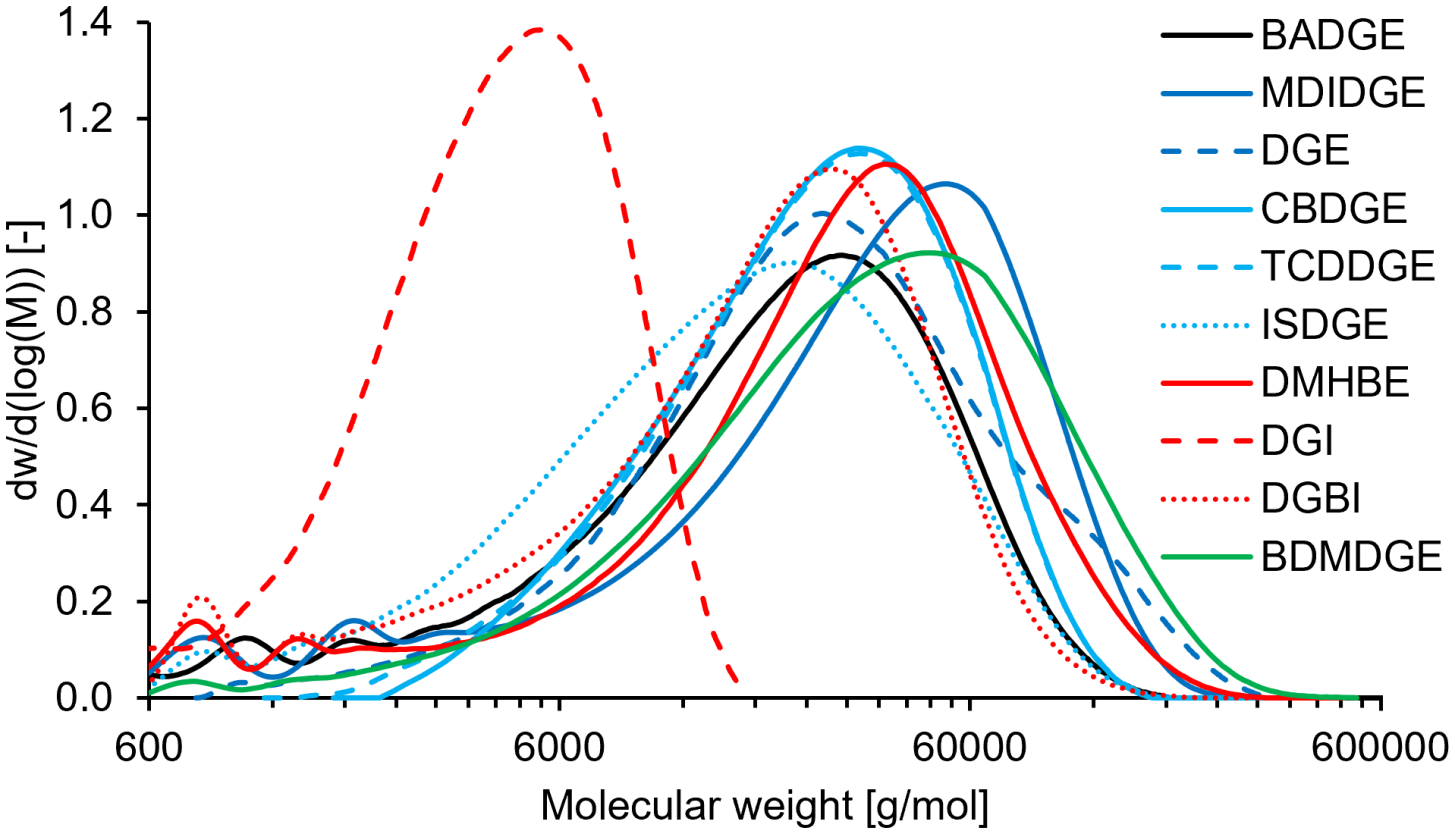
GPC molecular weight distributions for the different polymers. As the sample of the BADGE‐based material was measured for the reaction mixture at 300 min (i.e., before precipitation), there was also a high signal from the solvent at low molecular weight (not shown here to allow better comparison with the distribution of the other polymers, around 33 g/mol).

#### 3.2.2 Thermal Properties

The BADGE‐derived reference material exhibited a glass‐transition temperature (*T*_g_) of 170 °C and a degradation temperature (*T*_d_) of 365 °C (Figure 4). Both the MDIDGE‐ and DGE‐derived material showed equally high thermal stability, and these values are in the range of other amorphous high‐performance thermoplastics, like polysulfone or polyamide‐imide [11]. Although the TPADGE‐derived material had an even slightly higher *T*_g_ (178 °C), its degradation temperature was substantially lower (324 °C), and almost 25 % weight loss was observed after 1 h at 300 °C. The high *T*_g_ is likely due to the rigidity of the terephthalate units and the stronger intermolecular interactions (dipole of carbonyl moieties), since its reduced counterpart (BDMDGE) gave a much lower *T*_g_ (126 °C) [71]. Possibly, the ester linkages are less stable than the ether linkages in the other diglycidyl ethers and could undergo chain scission through β‐H transfer [72], since the BDMDGE‐based polymer had a much higher *T*_d_. Furthermore, the DGBI‐based polymer showed a high weight loss despite its elevated *T*_d_. High thermal stability was observed for the DGI‐, CBDGE‐, TCDDGE‐, and ISDGE‐based polyoxazolidinones on the other hand, with some polymers having a *T*_d_ > 400 °C and weight losses below 2 % after 1 h at 300 °C. In general, diepoxides with a bulky (CBDGE, TCDDGE, ISDGE) aliphatic backbone resulted in a lower *T*_g_ than the more planar, easily stacked, and rigid aromatic epoxides. TCDDGE, for example, had a *T*_g_ of 124 °C, which is rather low for an application as a high‐performance thermoplastic. Possibly, the *T*_g_ could still be further enhanced by increasing the molecular weight of the TCDDGE‐ and ISDGE‐derived polymer, as their *M*_*n*_ was still <20 kg/mol [71]. Lastly, DMHBE exhibited both high *T*_g_ (204 °C) and *T*_d_ (380 °C).

**FIGURE 4:**
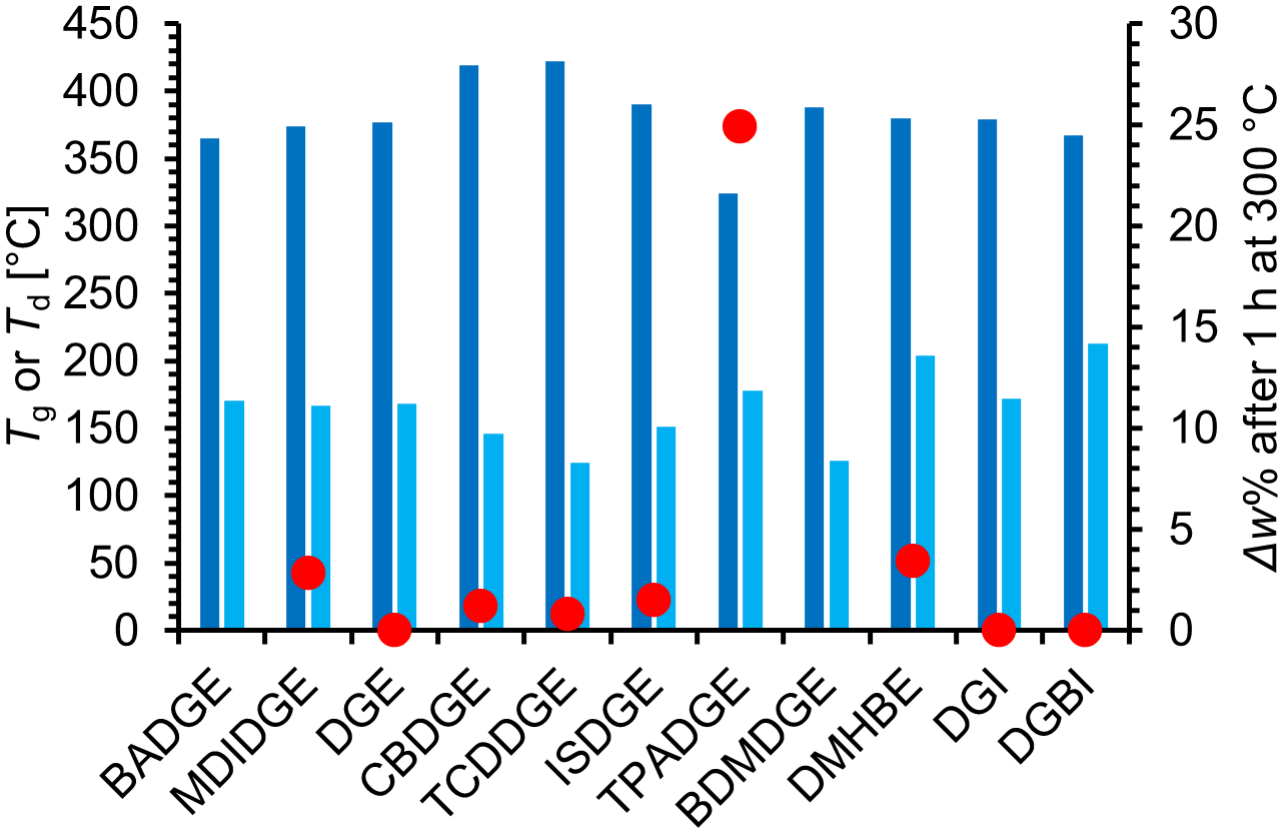
Thermal properties of the polyoxazolidinone polymers prepared with different diepoxides (dark blue: *T*_d_, blue: *T*_g_, red: Δ*w*% after 1 h at 300 °C).

#### 3.2.3 Mechanical Properties and Chemical Stability

In a preliminary test, the novel polyoxazolidinones were pressed into thin plates using a hot‐melt press (Figures S29, S33, S37, S41, S45, S49, S53, S57, and S61). Both for the MDIDGE‐ and DGE‐based polymers, flexible and transparent plates were obtained. While the MDIDGE‐polyoxazolidinone gave almost colourless plates, the DGE‐derived ones were slightly brownish and also slightly less bendable, but this might be due to the difficult processing of the fluffy polymer powder, which hampered an even spreading for the plate making. Moreover, CBDGE‐, TCDDGE‐, and DMHBE‐based polyoxazolidinone delivered colourless, transparent test plates with high flexibility. While flexible and transparent, ISDGE‐ and BDMDGE‐derived polyoxazolidinone plates were slightly brownish. As mentioned before, the TPADGE‐based polymer, on the other hand, was so brittle that it could not be processed into plates and was therefore not further tested. Also, plates prepared from DGI and DGBI proved quite brittle. While the DGI‐based polyoxazolidinone delivered brownish yet transparent plates, those made with the DGBI‐based material were brown and turbid. The poor mechanical properties of the latter material might be due to its low molecular weight (10.6 kg/mol) [71].

Furthermore, a preliminary solvent test with the MDIDGE‐ and DMHBE‐derived plates was conducted, in which the plates were submerged in acetone for 5 min. No optical change was observed for the polymers.

Next, micro‐injection‐moulded probes were prepared (see also Supporting Information) from all polymers except the DGBI‐ and TPADGE‐based material, which were then subjected to mechanical testing and compared to the BADGE‐based material (Figure S57). A first group of polyoxazolidinones with similar properties are the materials with methylene‐bridged diaryl moieties (BADGE‐, MDIDGE‐, and DGE‐based polyoxazolidinones). The reference BADGE‐based material is rather stiff (high *E*‐modulus) and exhibited an average elongation at break of around 80 % (Table 3 and Figure 5) and a tensile strength of 80 MPa, the latter of which is in the range of other amorphous high‐performance thermoplastics [73] (taking into account the small sample size). The mechanical properties of the MDIDGE‐derived polymer were, in all aspects, comparable to the BADGE‐based standard. The DGE‐based polyoxazolidinone was similar in hardness and strength to the MDIDGE‐based material, as would be expected from the similar molecular weight range, yet somewhat less ductile. This may be due to the difference in stacking of the polymer chains as the DGE‐based material should not contain 4‐oxazolidinone moieties. The MDIDGE‐ and DGE‐based polymers are of interest for applications in the automotive (*e.g.*, replace metal parts) or electr(on)ics industry (*e.g.*, battery housing) or in membrane applications when high thermal stability is required.

**FIGURE 5:**
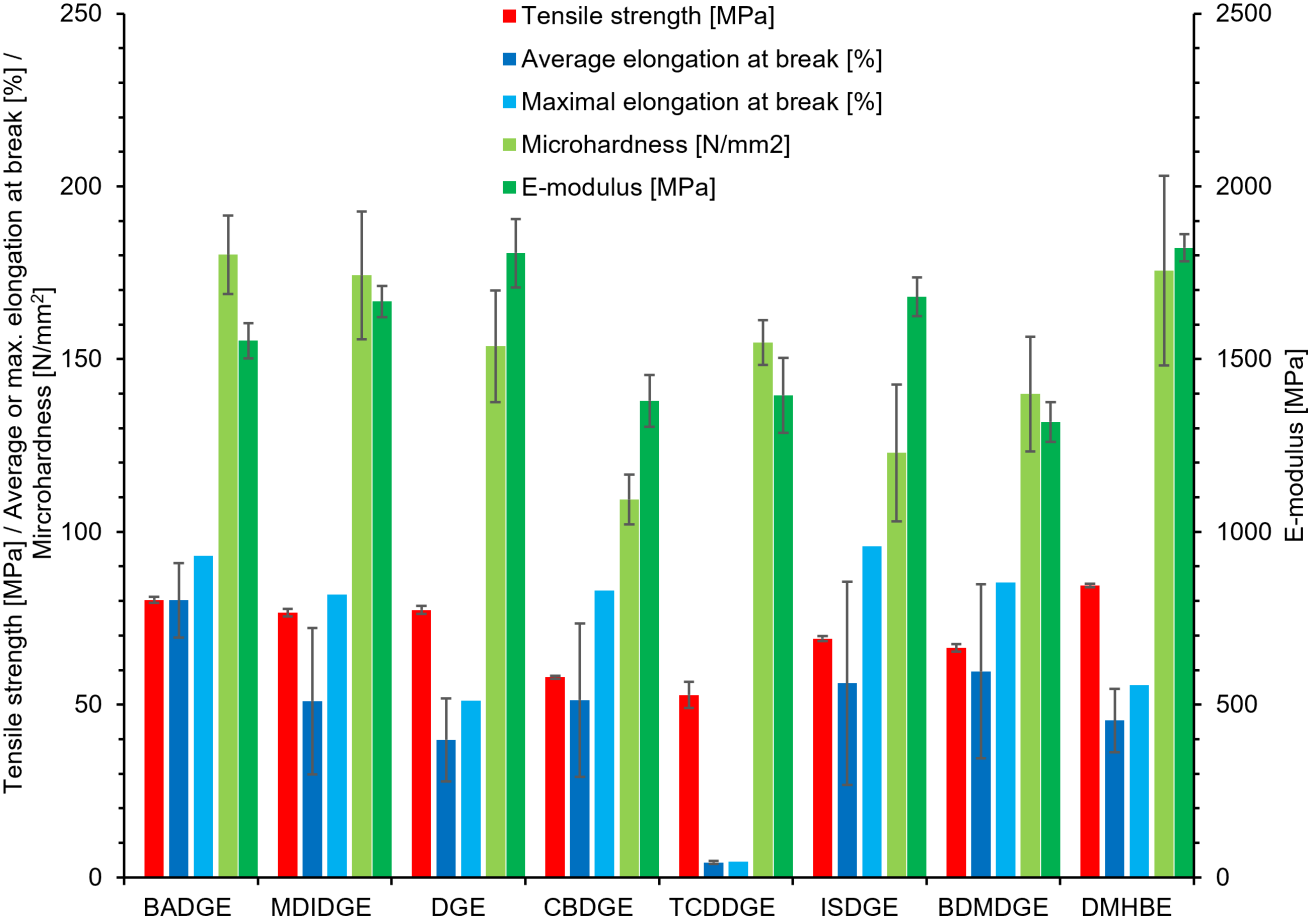
Mechanical properties of the different polyoxazolidinone polymers tested on micro‐injection‐moulded probes.

A second class are the polyoxazolidinones based on bulky nonaromatic diglycidyl ethers (CBDGE, TCDDGE, ISDGE). The CBDGE‐based polymer was less stiff and tough than the reference and MDIDGE‐ or DGE‐based polymers, although its average (51 %) and maximal elongation at break was comparable (83 %) with the results for the MDIDGE‐based polymer. This could be explained by the lower molecular weight of this polymer [71]. The CBDGE‐based polyoxazolidinone was also much softer than all other polymers tested. Although this material may be unsuitable as direct substitute for the BADGE‐derived polyoxazolidinone, the favourable elongation at break and high thermal stability may render it more suitable for applications requiring more flexible polymers with high thermal stability (such as solvent‐resistant thermoplastic coatings or membrane applications (for gas separation)). Further increasing of the monomer bulk, as in the TCDDGE‐based material, not only explains the low *T*_g_, but probably also weakens the interactions between the polymer chains to the extent that the material becomes neither ductile nor elastic, resulting in an insufficient performance in the tensile stress–strain test, breaking at small elongation (<5 %). In addition, the polymer had a rather low‐molecular weight, which could also have contributed to the weak mechanical performance. The ISDGE‐derived polymer, on the other hand, performed quite well despite its low‐molecular weight, with an *E*‐modulus and maximal elongation similar to the reference material. Possibly, slightly stronger interactions between the polymer chains exist due to the presence of stronger dipole–dipole interactions (fused tetrahydrofuran core instead of completely alicyclic core). On the other hand, the tensile strength proved somewhat lower, as well as the microhardness. Since tensile strength is related to the molecular weight, this could possibly be further improved by lowering the isocyanate‐to‐epoxide excess and the amount of chain regulator.

A third group are the *N*‐heterocyclic monomers (DMHBE, DGI, and DGBI), for which strong dipole–dipole interactions are expected between the polymer chains. Good mechanical properties were nevertheless obtained with the DMHBE‐based polyoxazolidinone, which surpassed the BADGE‐based polymer in terms of *E*‐modulus and tensile strength. Its microhardness was very similar to that of the BADGE reference material. Although somewhat harder and stiffer, the DMHBE‐derived polyoxazolidinone was a bit less ductile (with an average (45 %) and maximal (56 %) elongation at break). On the other hand, the polymer based on the less sterically hindered DGI proved too brittle to be moulded reproducibly into homogeneous probes so that its mechanical properties could not be determined. The even more brittle, low‐molecular‐weight DGBI‐based polymer, where additional interactions are expected due to stacking of the aromatic moieties, could not be subjected to mechanical testing either.

Finally, while the TPADGE‐based polyoxazolidinone was far too brittle for mechanical testing, likely due to the high amount of isocyanurate groups and due to strong stacking and dipole–dipole interactions of the flat phthalate moieties. On the other hand, the material based on BDMDGE turned out to be rather elastic and sticky. Nevertheless, test specimens could be prepared, and the polymer had similar properties to the CBDGE‐derived material.

## 4 Conclusion

In this work, ten diglycidyl ethers were introduced as bisphenol A‐free sustainable building blocks for polyoxazolidinone polymers, being either biobased or derived from plastic waste streams.

With all evaluated building diepoxides, a polymer with MDI as isocyanate and *p*‐*t*BPGE as chain regulator could be obtained using PPh_4_Cl as a catalyst. The chain length, and hence the molecular weight (10–40 kg/mol), could be controlled by varying the epoxide‐to‐isocyanate ratio, as well as the amount of end cap, yet for high targeted values, the real chain length deviated more. The most promising monomers to replace BADGE were MDIDGE, DGE, and DMHBE, which delivered materials with similar thermal properties (*T*_g_ around 170 °C, for DMHBE even > 200 °C) and thermal stability up to *ca*. 370 °C and similar mechanical behavior in terms of stiffness (*E*‍‐‍modulus around 1.5 GPa), strength (tensile strength *ca*. 80 MPa) and ductility. Moreover, the MDIDGE‐ and DMHBE‐based materials exhibited good acetone resistance in a preliminary solvent test.

While also CBDGE, BDMDGE, and ISDGE delivered thermally stable materials (all with *T*_d_ > 385 °C), their *T*_g_ was considerably lower, and CBDGE‐ and BDMDGE‐derived polymers were not as hard and strong as the BADGE‐based reference. ISDGE‐based polyoxazolidinone exhibited a relatively high (1.68 GPa) *E*‐modulus, but was more brittle and softer than the BADGE‐based polyoxazolidinone. Likely, the properties of this polymer could still be improved by increasing its molecular weight. Nevertheless, these materials could be attractive for applications that require thermal and chemical stability with somewhat higher flexibility, like coatings or gas separation membranes. For this, elaborate further testing of the materials in those applications is necessary, which falls outside the scope of this work. The mechanical and thermal properties of the TCDDGE‐, TPADGE‐, DGI‐, and DGBI‐based polymers were not sufficient for high‐performance thermoplastic applications.

## Conflicts of Interest

The authors declare no conflicts of interest.

## Supporting information

Supplementary Material

## Data Availability

The data that support the findings of this study are included in the Supporting Information of this paper (such as detailed experimental procedures, NMR spectra, mechanical testing results) or available from the corresponding authors upon reasonable request.
