# Supplementary material for: Sustainable Diepoxide Monomers for Polyoxazolidinone‐Based High‐Performance Thermoplastics
Source: ChemSusChem. 2026 Oct 10;19(19):e71140. doi: 10.1002/cssc.71140 (PMC13662711; doi:10.1002/cssc.71140)
Supplement: Supplementary file 1 [file CSSC-19-e71140-s001.pdf]

# Sustainable diepoxide monomers for polyoxazolidinone-based high performance thermoplastics

Lisa Van Emelen,*+[a] Friederike Ratsch, [a]+, Patrick Klein, [a] Sarah Brettschneider, [a] Hans Gildenast, [a] Markus D. Schötz, [a] Mathias Glaßner, [a] Martin Reiher, [a] Elena Dirksen, [a] Felix Niebisch,[a] Kiruthika Periasamy,[a] Ria Ghosh, [a] Sohajl Movahhed,[b] Kai Laemmerhold[b] and Jürgen Klankermayer.*[a]

[a] CAT Catalytic Center, Institut für Technische und Makromolekulare Chemie, RWTH Aachen, Worringer Weg 2, 52074 Aachen, Germany.

[b] Covestro Deutschland AG, Kaiser-Wilhelm-Allee 60, 51373 Leverkusen, Germany.

+ Co-first authors

* Corresponding authors

## Supporting information

Total number of pages: 57

Total number of figures: 62

Total number of tables: 3

# Contents

Contents ... 2
General ... 4
Chemicals ... 4
Characterization ... 5
NMR measurements ... 6
FTIR-spectroscopy ... 6
GPC ... 7
Differential scanning calorimetry ... 7
Thermogravimetric analysis (TGA) ... 7
Mechanical testing ... 8
Monomer synthesis ... 10
BADGE ... 10
MDIDGE ... 10
DGE ... 12
CBDGE ... 13
TCDDGE ... 14
ISDGE ... 15
TPADGE ... 17
BDMDGE ... 17
DMHBE ... 18
DGI ... 18
DGBI ... 21
Polymer synthesis ... 22
BADGE-derived polyoxazolidinone ... 22
MDIDGE-derived polyoxazolidinone ... 25
DGE-derived polyoxazolidinone ... 29
CBDGE-based polyoxazolidinone ... 33

TCDDGE-derived polyoxazolidinone ..... 37
ISDGE-derived polyoxazolidinone ..... 41
TPADGE-derived polyoxazolidinone ..... 45
BDMDGE-derived polyoxazolidinone ..... 46
DMHBE-derived polyoxazolidinone ..... 48
DGI-derived polyoxazolidinone ..... 52
DGBI-derived polyoxazolidinone ..... 54
Targeted molecular weight *versus* attained molecular weight ..... 56
References ..... 57

# General

## Chemicals

**Caution:** epoxides and isocyanates may undergo exothermic reactions! The internal temperature was monitored in all reactions. Leftover epoxide and isocyanate were quenched before disposal. Epoxides were handled wearing LLDPE-laminated gloves (Ansell AlphaTec 02-100).

Chemicals (**Table S1**) were used as received, unless stated otherwise.
4,4'-Methylenebis(phenyl isocyanate) (MDI 44 M flakes) was delivered from the Covestro MDI plant in Krefeld-Uerdingen and stored at -18 °C in a freezer for not more than 6 months. Bisphenol A diglycidylether (BADGE, D.E.R. 332, Sigma Aldrich, < 0.3 wt% water from Karl-Fischer titration) and sulfolane (Acros Organics, 99 %) were heated to 160 °C and 50 °C respectively, prior to use. Benzonitrile is heated over $CaH_2$ over night at 80 °C and then distilled off before use (< 0.1 wt% water).

***Table S1.*** *Overview of chemicals with purity and supplier*

| Compound | Purity | Supplier |
|---|---|---|
| 4,4'-methylenebis(phenyl isocyanate) | ≥ 98.2 % | Covestro |
| *p*-*tert*-butylphenyl glycidyl ether | Not mentioned | Abcr |
| *N*-methylpyrrolidone | 99 % | Acros Organics |
| Tetraphenylphosphonium chloride | 99 % | Sigma-Aldrich |
| Bisphenol A diglycidyl ether (BADGE, D.E.R. 332) | ~ 95 % | Sigma-Aldrich |
| Sulfolane | 99 % | Acros Organics |
| *Ortho*-dichlorobenzene | > 98 % | Fisher Scientific |
| Benzonitrile | > 99 % | Sigma-Aldrich |
| MDI-diol (Mediol) | Technical grade | Covestro |
| Epichlorohydrin | ≥ 99 % | Sigma-Aldrich |
| NaOH | 98 % | Thermoscientific |
| Tetrabutylammonium bromide | ≥ 98 % | Sigma-Aldrich |
| Dichloromethane | 99.90 | Julius Hoesch, GmbH |
| $MgSO_4$ | ≥ 97 %, anhydrous | Carl Roth |
| NaCl | ≥ 99.0 % | Sigma-Aldrich |
| Silica gel, for chromatography, 0.035-0.070 mm 60A | - | Thermo Scientific |
| Ethyl acetate | 99.5 | Julius Hoesch GmbH |
| Cyclohexane | 99.8 | Julius Hoesch GmbH |
| Allyl glycidyl ether | ≥ 99.0 % | Sigma-Aldrich |
| *Meta*-chloroperbenzoic acid | ≤ 77 % | Aldrich |
| $Na_2S_2O_3 . 5 H_2O$ | ≥ 99.5 % | Carl Roth |
| 2,2,4,4-tetramethyl-1,3-cyclobutanediol | 98 % | Eastman |
| Acetic acid | 100 %, Ph. Eur. | Carl Roth |
| Tricyclodecanedimethanol | 96 % | Sigma-Aldrich |
| Dimethylhydantoin bisepoxide | 90 % | BLD Pharm |

***Table S1.*** *Overview of chemicals with purity and supplier (continued)*

| Compound | Purity | Supplier |
|---|---|---|
| 2-imidazolidinone | > 97.0 % | TCI |
| Acetone | 99.5 | Julius Hoesch GmbH |
| Dimethylsulfoxide | > 99.9 % | Sigma-Aldrich |
| Ethanol | ≥ 99.6 % | Julius Hoesch |
| 1,3-dihydro-2*H*-benzo[*d*]imidazole-2-one | > 98.0 % | TCI |
| Dimethylsulfoxide-$d_6$, 0.03 V% TMS | 99.8 % D | Eurisotop |
| Chloroform-d, 0.03 V% TMS | 99.8 % D | Eurisotop |
| Isosorbide | > 98 % | TCI |
| Allyl bromide | 97 % | Sigma-Aldrich |
| Oxone (2 $KHSO_5 \cdot KHSO_4 \cdot K_2SO_4$) | > 4.0 % (active oxygen basis) | Sigma-Aldrich |
| Diethyl ether | 99.5 | Julius Hoesch, GmbH |
| Terephtalic acid diglycidyl ether (Denacol Ex-711) | 95 | Nagase |
| 1,4-benzenedimethanol | > 99.0 | TCI |
| Aluminium oxide, for chromatography, neutral, Brockman I, 40-300 µm, 60A) | - | Acros |
| LiBr | ≥ 99.0 %, anhydrous | Sigma-Aldrich |
| *N*,*N*-dimethylacetamide | 99.8+ % | Thermo Scientific |
| 2,6-di-*tert*-butyl-4-methylphenol | > 99.0 % | Aldrich |
| $CaH_2$ | 93 % | Thermo Scientific |

## Characterization

The average chain length of the thermoplastic polyoxazolidinones was controlled by the molar ratio of bisepoxide, diisocyanate and/or monofunctional end cap. Carother's equation[1] allows to calculate the average chain length $n$ in the polymeric product obtained with a diisocyanate and a bisepoxide:

$$n = (1 + q) / (1 + q - 2pq) \qquad (1)$$

with $q = n_x / n_y \leq 1$ the molar ratio of diisocyanate and bisepoxide, with the molar amount of the limiting reagent ($n_x$) in the nominator and the reagent in excess ($n_y$) in the denominator; and the conversion p.

The number average molecular weight ($M_n$) of the thermoplastic polyoxazolidinones can be calculated as follows:

$$M_n = n*((M_{diisocyanate}+M_{bisepoxide})/2) + (2*M_{end\ cap}) \qquad (2)$$

With $M_{diisocyanate}$, $M_{bisepoxide}$, and $M_{end\ cap}$ being the molar masses of the diisocyanate, bisepoxide and end cap, respectively.

***NMR measurements***

*$^{1}H$-NMR.* $^{1}H$-NMR spectra were recorded at 25 °C on a Bruker AV III 400 Spectrometer with the zg30 pulse program (p1 10 μs; plw1 16 W; ds 2; ns 16; d1 1 s; aq 4,089 s; sw 20 ppm) or on a Bruker AV III 300 Spectrometer with the zg30 pulse program (p1 10.5 μs; plw1 16 W; ds 2; ns 16; d1 1 s; aq 3.985 s; sw 27.45 ppm). Samples were measured in DMSO-$d_6$ or $CDCl_3$. In addition, $^{1}H,^{1}H$-COSY, $^{1}H,^{13}C$-HSQC and $^{1}H,^{13}C$-HMBC 2D liquid NMR spectra were recorded on the same equipment for product identification.

*$^{13}C$-NMR.* $^{13}C$-NMR spectra were recorded at 25 °C on a Bruker AV III 400 Spectrometer with a sample case and a 5 mm PABBO BB/19F-1H/D probe with z-gradients and ATM accessory for automatic tuning and matching, using the zgpg30 pulse program (p1 9,75 μs; plw1 75,5 W; ds 4; ns 2048; d1 2 s; aq 1,36 s; sw 600 ppm) or on a similarly equipped Bruker AV III 300 Spectrometer with the zgpg30 pulse program (p1 10.30 μs; plw1 45 W; ds 4; ns 2048; d1 2 s; aq 1,36 s; sw 319 ppm). Samples were measured in DMSO-$d_6$ or $CDCl_3$.

Based on the ratio of the signals for the protons in the *tert*-butyl moiety ($I_{tert\text{-butyl}}$, C**H₃** at 1.26 ppm) of the chain regulator *p*-*t*BPGE, and the oxazolidinone unit ($I_{oxa}$,C-**H** at 5.05 ppm), the number average molecular weight ($M_n$) of the polymer can be calculated.
The number of repeating units *n* can then be calculated from the following formula:

$$I_{oxa} / (2n + 2) = I_{tert\text{-butyl}} / 18$$

With *n*, $M_n$ can be derived.

***FTIR-spectroscopy***

Solid-state FTIR analyses were performed on a Bruker ALPHA-IR spectrometer equipped with a diamond probe head. The software OPUS 7.0 was used for data treatment. A background spectrum was recorded against ambient air. Thereafter, a small sample of the thermoplastic polyoxazolidinone (2 mg) was applied to the diamond probe and the FTIR spectrum recorded averaging over 24 spectra obtained in the range of 4000 to 400 $cm^{-1}$ with a resolution of 4 $cm^{-1}$. Typical FTIR spectra are shown in **Figures S20, S24, S28, S32, S36, S40, S44, S45, S48, S52** and **S56** and relevant vibrations are listed in **Table S2**.

***Table S2.*** *Relevant FTIR vibrations and corresponding wave numbers for polyoxazolidinone, the starting materials and possible side products.*

| Wave number [$cm^{-1}$] | Functional group | Vibration |
|---|---|---|
| 905-910 | Epoxide | Ring deformation |
| 1410 | Isocyanurate | Ring deformation |
| 1710-1715 | Isocyanurate | C=O vibration |
| 1740-1760 +/- 10 | Oxazolidinone | C=O vibration |
| 2250 | Benzonitrile | C≡N vibration |
| 2260 | Isocyanate | N=C=O vibration |

***GPC***

The determination of the number average molecular weights, weight average molecular weights, and the polydispersity index were carried out by gel permeation chromatography (GPC). GPC was performed on an Agilent 1100 Series instrument with *N,N'*-dimethylacetamide (DMAc) + LiBr (1.7 g/l) as the eluent, PSS GRAM analytical columns (1 x 100 Å, 2 x 3000 Å) from PSS, equipped with a refractive index (RI) detector. The column flow rate in all measurements was set to 1 ml/min. For determining molecular weights, the calibration was performed with poly(styrene) standards (ReadyCal-Kit PS - $M_p$ 370-2520000 Da from PSS). The samples were analyzed using PSS WinGPC UniChrom V 8.2 Software.

***Differential scanning calorimetry***

The glass transition temperature ($T_g$) of the polyoxazolidinones was determined *via* differential scanning calorimetry (DSC). Measurements were performed using a STARe DB V11.00 from Mettler Toledo equipped with STARe Software V16.30a. 10 mg samples were weighed in disposable alumina crucibles (100 µl). The DSC measurements were conducted under a constant flow of argon (10 ml/min). Initially, the sample was brought at 30 °C and held at this temperature for 3 min. Then, the sample was heated at a heating rate of 10 °C/min to 250 °C. After reaching this temperature, the sample was cooled down again to 30 °C at a cooling rate of 10 °C/min. The heating-cooling cycle was repeated twice. The second cycle was used to determine the $T_g$.

***Thermogravimetric analysis (TGA)***

The degradation temperature ($T_d$) of the polyoxazolidinone polymers was measured *via* thermogravimetric analysis (TGA) under inert conditions on a Mettler Toledo STARe DB V11.00. For data analysis the software STARe software V16.30a was used. To study the thermal stability at 300 °C over time, 10-20 mg polyoxazolidinone was weighed in a 150 µl alumina pan (previously cleaned at 800 °C for 8 h) and heated from 25 °C to the target temperature (300 °C) at a heating rate of 10 °C/min under argon (15 ml/min). The sample was

then kept at this temperature for 1 h, during which the relative weight loss was recorded as a function of time. The $\Delta w$% given in the examples is the weight loss percentage of the sample after 1 h at the target temperature. For the $T_d$, samples were kept at 25 °C for 3 min and then heated at 10 °C/min to 600 °C under an Ar-flow (15 ml/min). Afterwards the samples were cooled down to 25 °C at a rate of 50 °C/min.

***Mechanical testing***

Mechanical properties of the polymers were measured at the KUZ Leipzig on injection-moulded samples (see **Figure S1**). For the injection moulding, a formicaPLAST piston plasticizing apparatus with a 7 mm pre-plasticizing piston and a 3 mm injection piston was employed. The maximum shot volume of the standard version is 150 mm³. The main parameters for the injection moulding are listed in **Table S3**.

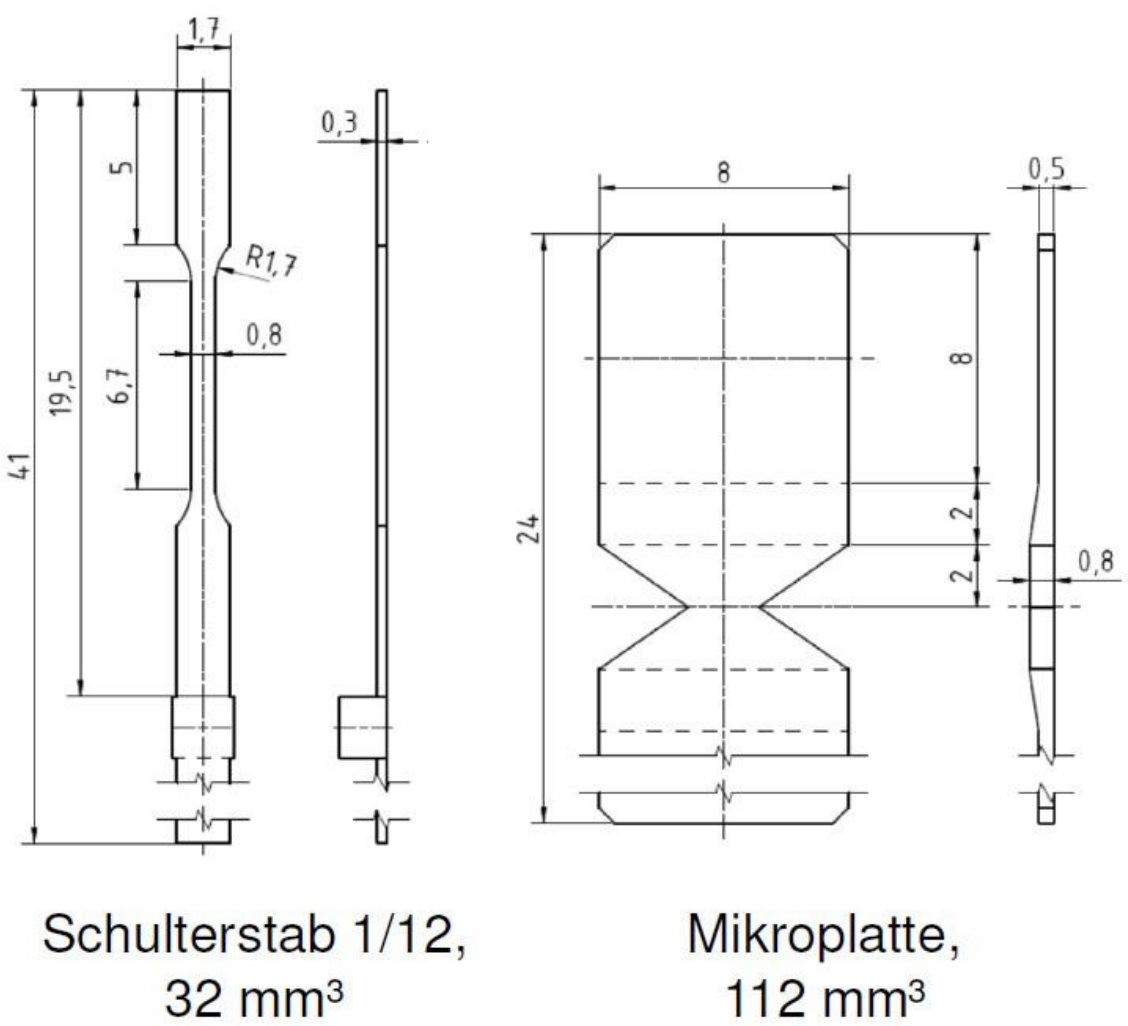


***Figure S1.*** *Dimensions of the micro-injection moulded test samples.*

The tensile tests were performed on a Zwick material testing machine (Zwicki Z2.5/TN), with a 200 N load cell. The determination of the Young's modulus was done using crosshead displacement, so that for such small samples the measurements should be carefully interpreted. Microhardness, on the other hand, was measured using a method similar to DIN EN ISO 14577-1:2003-05 on an inspect micro S500N device (Hegewald & Peschke Meß- und Prüftechnik GmbH) equipped with LabMaster Version 2.5.0.0. The data were analyzed with IdentAnalyser Version 2.5.0 (ASMEC GmbH). The maximal applied force was 2 N, and the measurements were done in force-controlled mode at a rate of 5 N/s and with a hold time of 30 s.

***Table S3.*** *Settings for injection moulding.*

| Epoxide monomer | $T_{cylinder}$ [°C] | $T_{equipment}$ [°C] | Injection rate [mm/s] |
|---|---|---|---|
| BADGE | 290 | 140 | 140 |
| MDIDGE | 290 | 130 | 120 |
| DGE | 230 | 120 | 200 |
| CBDGE | 260 | 120 | 130 |
| TCDDGE | 240 | 120 | 80 |
| ISDGE | 250 | 140 | 180 |
| BDMDGE | 260 | 120 | 120 |
| DMHBE | 320 | 150 | 200 |
| DGI | 270 | 120 | 250 |

# Monomer synthesis

## BADGE

Bisphenol A diglycidyl ether is commercially available (Sigma Aldrich, D.E.R. 332) and was used as received.

## MDIDGE

MDI-diol diglycidyl ether was synthesized from the MDI-diol (Mediol) obtained in technical grade from Covestro through glycidylation according to the literature (**Figure S2**).[2] In a flask equipped with a reflux condenser, Mediol (40 g, 0.1004 mol), epichlorohydrin (11.7 equiv.), NaOH (10 equiv.) and tetrabutylammonium bromide (TBAB, 10 mol%) were introduced at rt. Next, 0.3 equiv. water was added, and the mixture was stirred at room temperature, using ice to keep the temperature low. After 40 min the temperature was gradually increased to 75 °C and the reaction was monitored by TLC. After 2 h, the reaction was complete and dichloromethane was added, after which the mixture was filtered to remove insoluble residues. Subsequently, the dichloromethane phase was washed with water (3 x) and the first aqueous phase was re-extracted with dichloromethane. The organic phase was washed with brine and then dried over $MgSO_4$, filtered, and the solvent was removed under vacuum. Finally, the crude product was purified over a silica pad using an eluent gradient from 1:1 cyclohexane:ethyl acetate to pure ethyl acetate, yielding 49 % of pure MDIDGE.

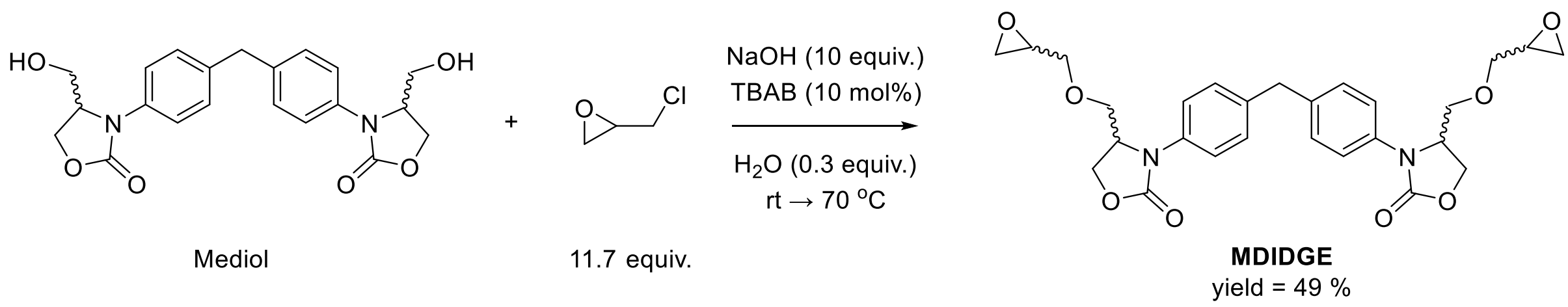


***Figure S2.*** *Diglycidylation of the MDI-based diol.*

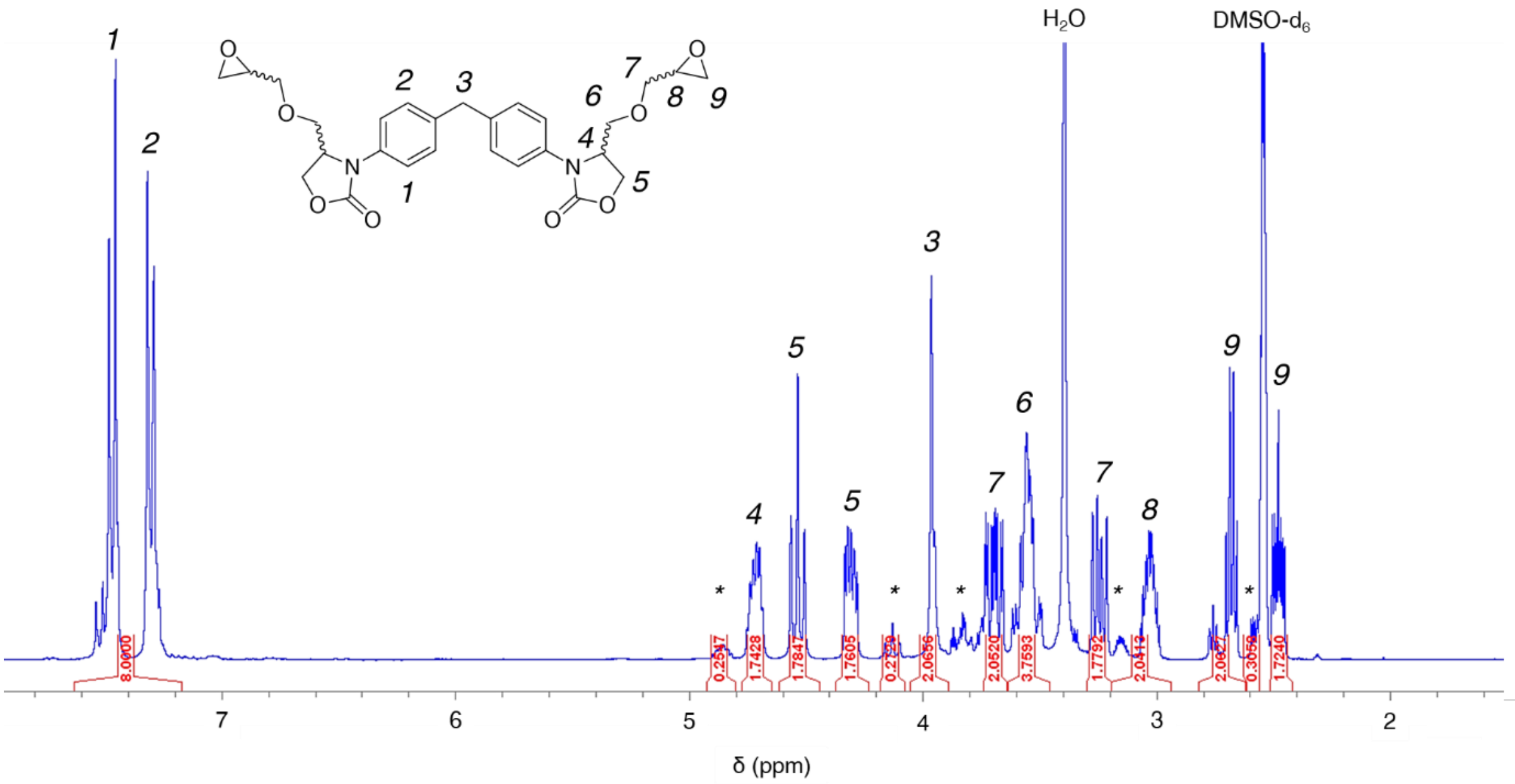


***Figure S3.*** *$^{1}$H-NMR spectrum of MDIDGE in DMSO-d$_6$ (300 MHz).*

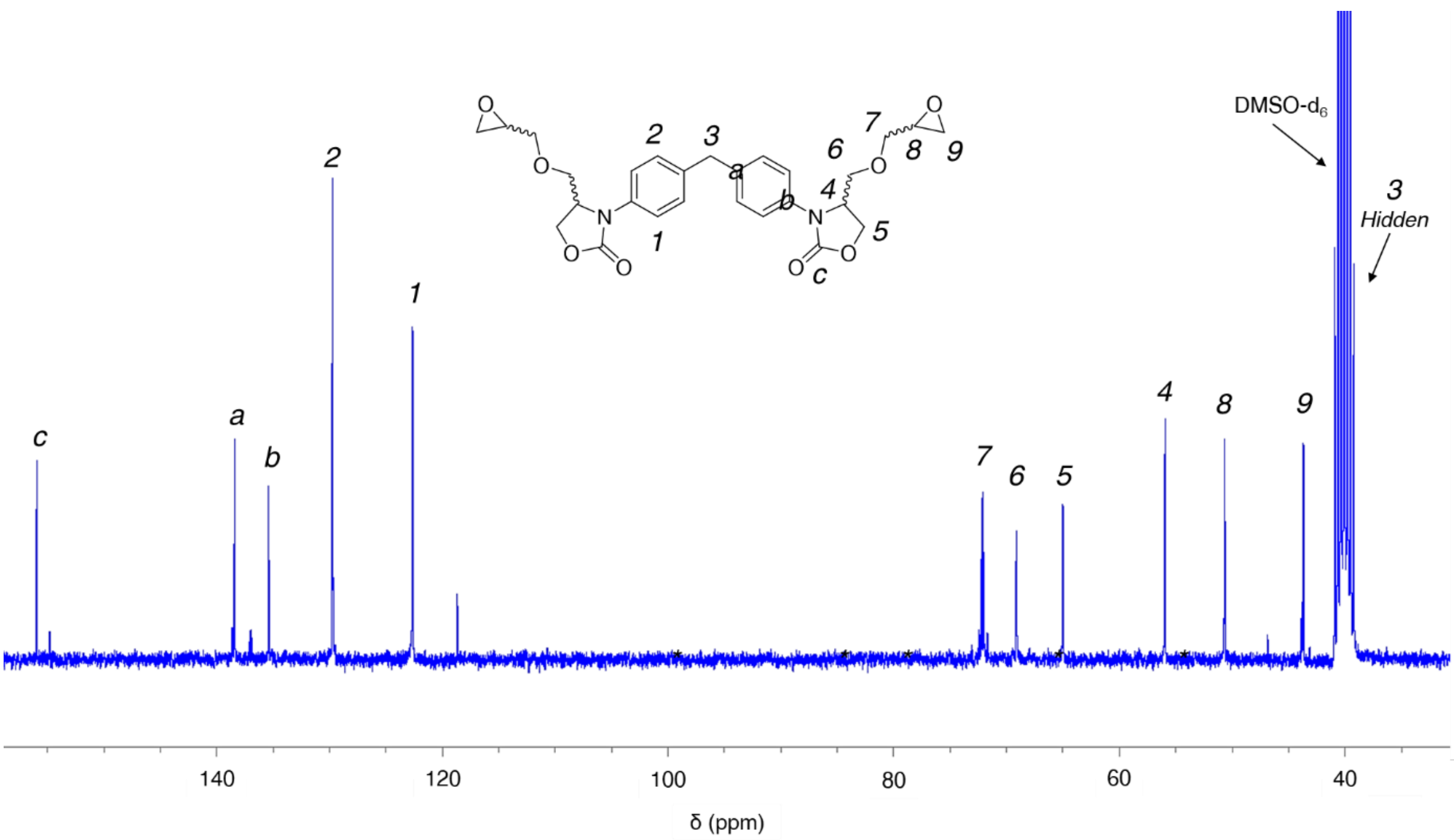


***Figure S4.*** *$^{13}$C-NMR spectrum of MDIDGE in DMSO-d$_6$ (300 MHz).*

## DGE

DGE (diglycidyl ether) was obtained through epoxidation of allyl glycidyl ether following a literature procedure (**Figure S5**).[3] Allyl glycidyl ether (0.2125 mol, 25 ml) was dissolved in 500 ml dichloromethane in a round bottom flask equipped with a reflux condenser, stirred, and cooled on ice to 0 °C. Then, 0.3000 mol *meta*-chloroperbenzoic acid (70 %, 1.4 equiv.) was added over 1 h, while the reaction mixture was allowed to heat to room temperature. The mixture was left to stir for 2 days and subsequently filtered, followed by a washing step with ethyl acetate. Next, the crude product was stirred at 0 °C in a $Na_2S_2O_3$ solution. After filtration and three washing steps with a 0.5 M NaOH solution, the product was dried under reduced pressure. Finally, the diepoxide was distilled from $CaH_2$ (42 % yield).

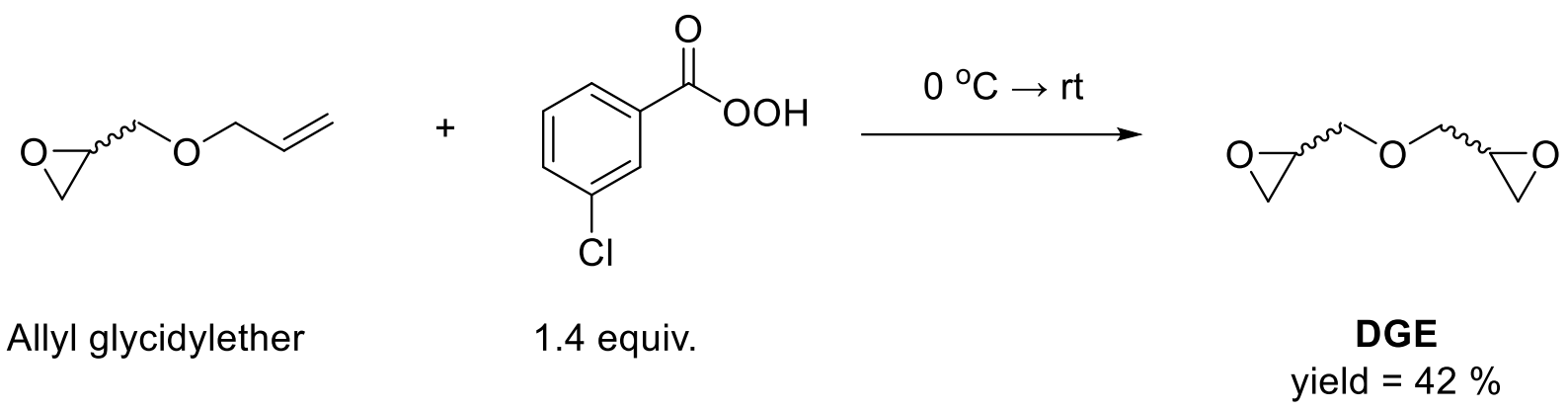


***Figure S5.*** *Epoxidation of allyl glycidylether.*

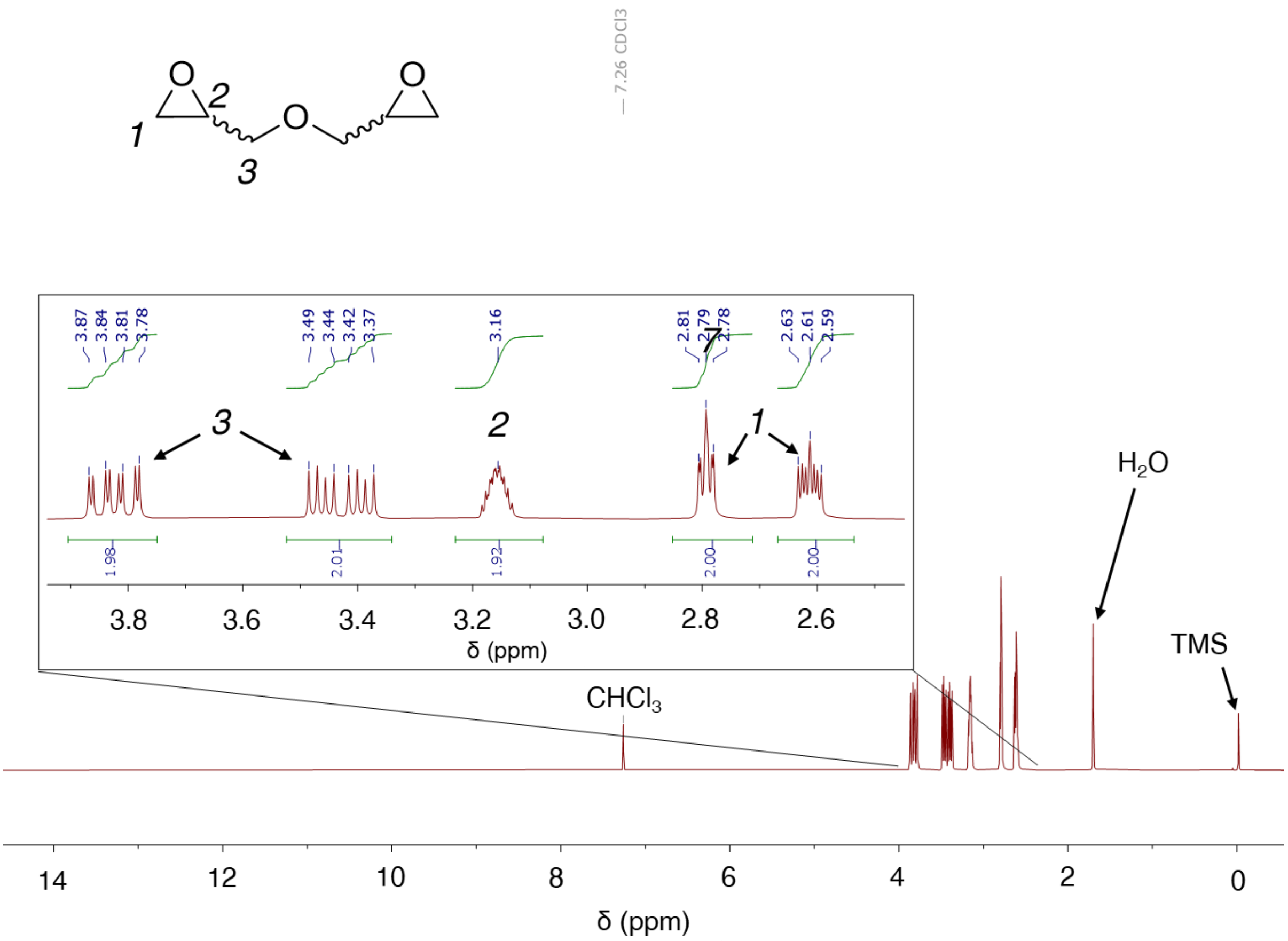


***Figure S6.*** *$^{1}$H-NMR spectrum of DGE in $CDCl_3$ (300 MHz).*

## CBDGE

The corresponding diol (2,2,4,4-tetramethyl-1,3-cyclobutanediol) was diglycidylated following a literature procedure (**Figure S7**).[2] 2,2,4,4-tetramethyl-1,3-cyclobutanediol (12.50 g, 0.0867 mol), epichlorohydrin (8 equiv.), NaOH (10 equiv.) and tetrabutylammonium bromide (10 mol%) were combined in a round-bottom flask equipped with a reflux condenser, stirred, and cooled on ice to 0 °C, after which 0.3 equiv. water was added. The mixture was left stirring, and the ice was removed, so that the mixture gradually heated to 40 °C over 165 min. The temperature was kept between 37 and 40 °C using a water bath, and after 310 min (total reaction time) the reaction was complete, after which dichloromethane was added to the mixture, which was filtered. The organic phase was washed with water, an aqueous solution of AcOH (*pH* ~ 4) and brine. The dichloromethane phase was then dried over $MgSO_4$, filtered and the solvent was removed under vacuum. The crude product was further purified *via* vacuum distillation, yielding the pure diepoxide in 89 % yield.

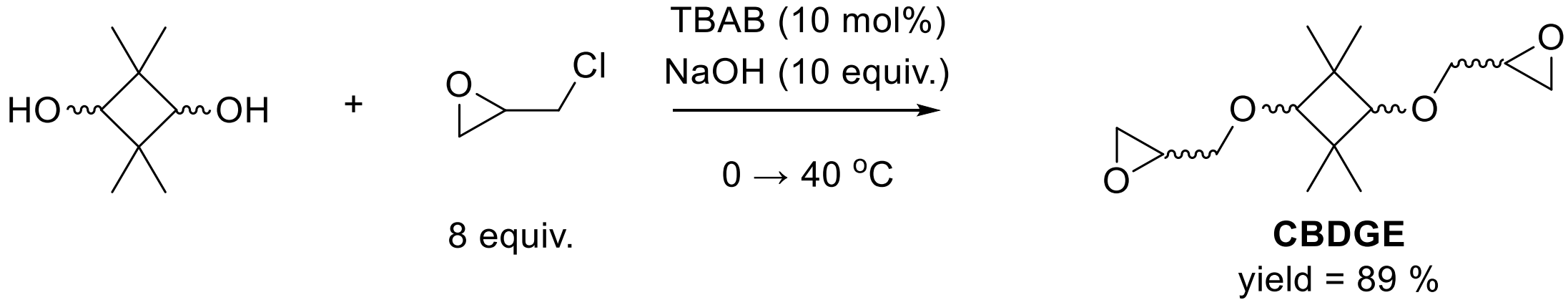


***Figure S7.*** *Diglycidylation of 2,2,4,4-tetramethyl-1,3-cyclobutanediol.*

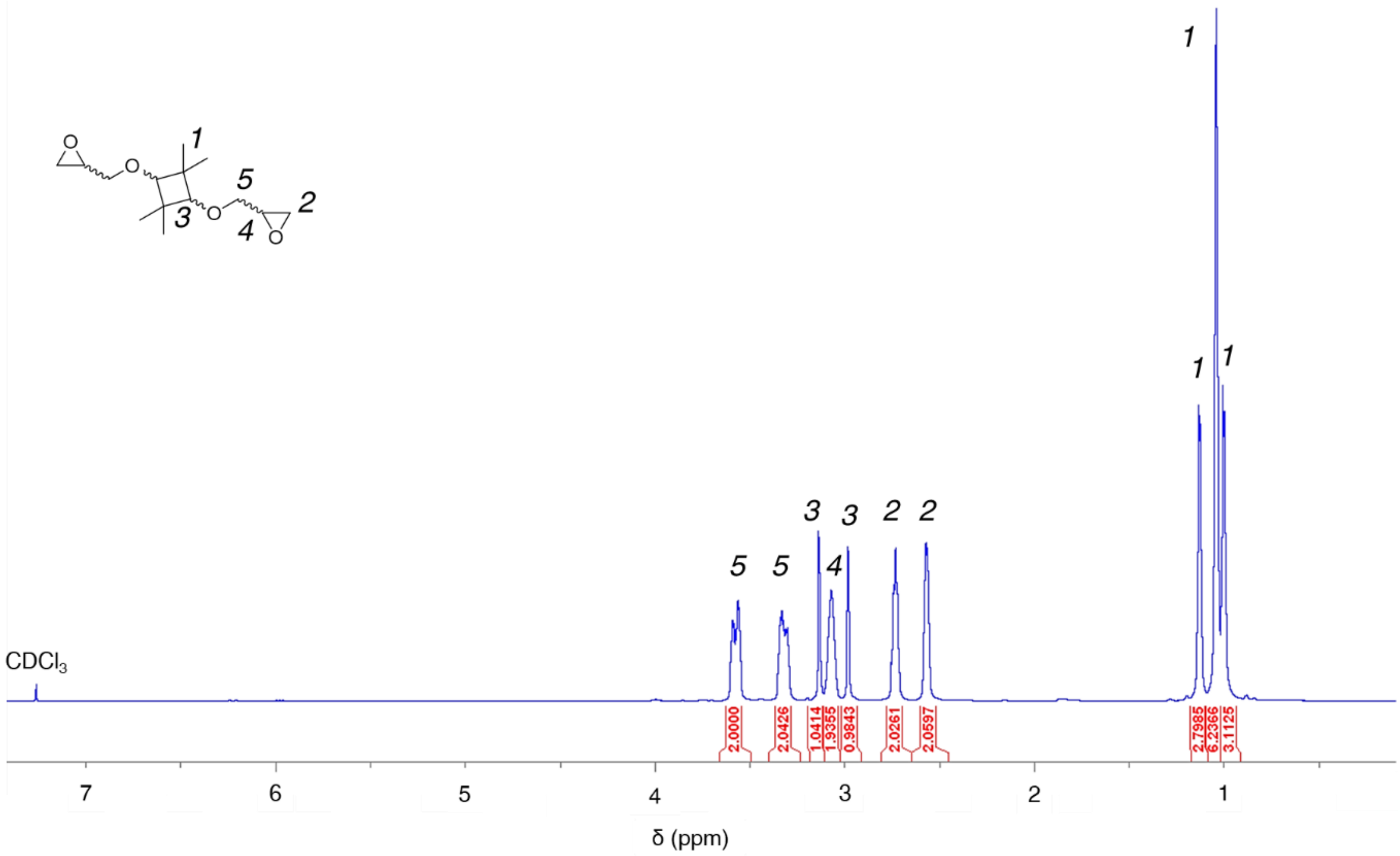


***Figure S8.*** *$^1$H-NMR spectrum of CBDGE in $CDCl_3$ (400 MHz).*

## TCDDGE

Tricyclodecanedimethanol diglycidyl ether was synthesized by glycidylation of tricyclodecanedimethanol (TCD alcohol) following a standard procedure (**Figure S9**).[2] 300 ml of a 40 w% NaOH solution was prepared, in which 0.2038 mol (40.00 g) TCD alcohol and 10 mol% TBAB were dissolved. For 1 h the mixture was warmed to 55-60 °C and stirred under Ar. Next, the solution was cooled to 0 °C using an ice bath and 10 equiv. of epichlorohydrin were added dropwise. After the addition, the mixture was left to heat up gradually to 27 °C and was stirred overnight (water bath). Subsequently, the product was extracted with dichloromethane (3 x 100 ml) and the combined organic phases were washed with water (3 x) and brine and dried over $MgSO_4$. After solvent removal under vacuum, the crude product was purified by vacuum distillation, resulting in 65 % TCDDGE yield.

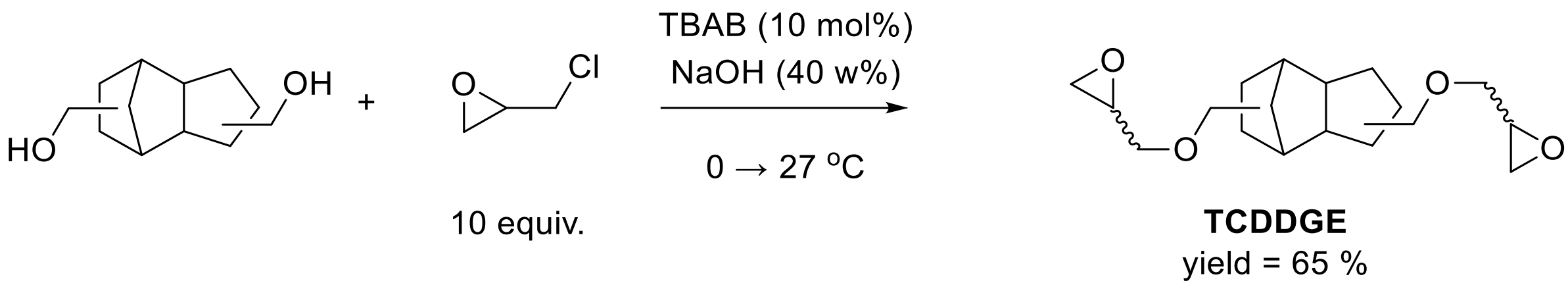


***Figure S9.*** *Diglycidylation of tricyclodecanedimethanol.*

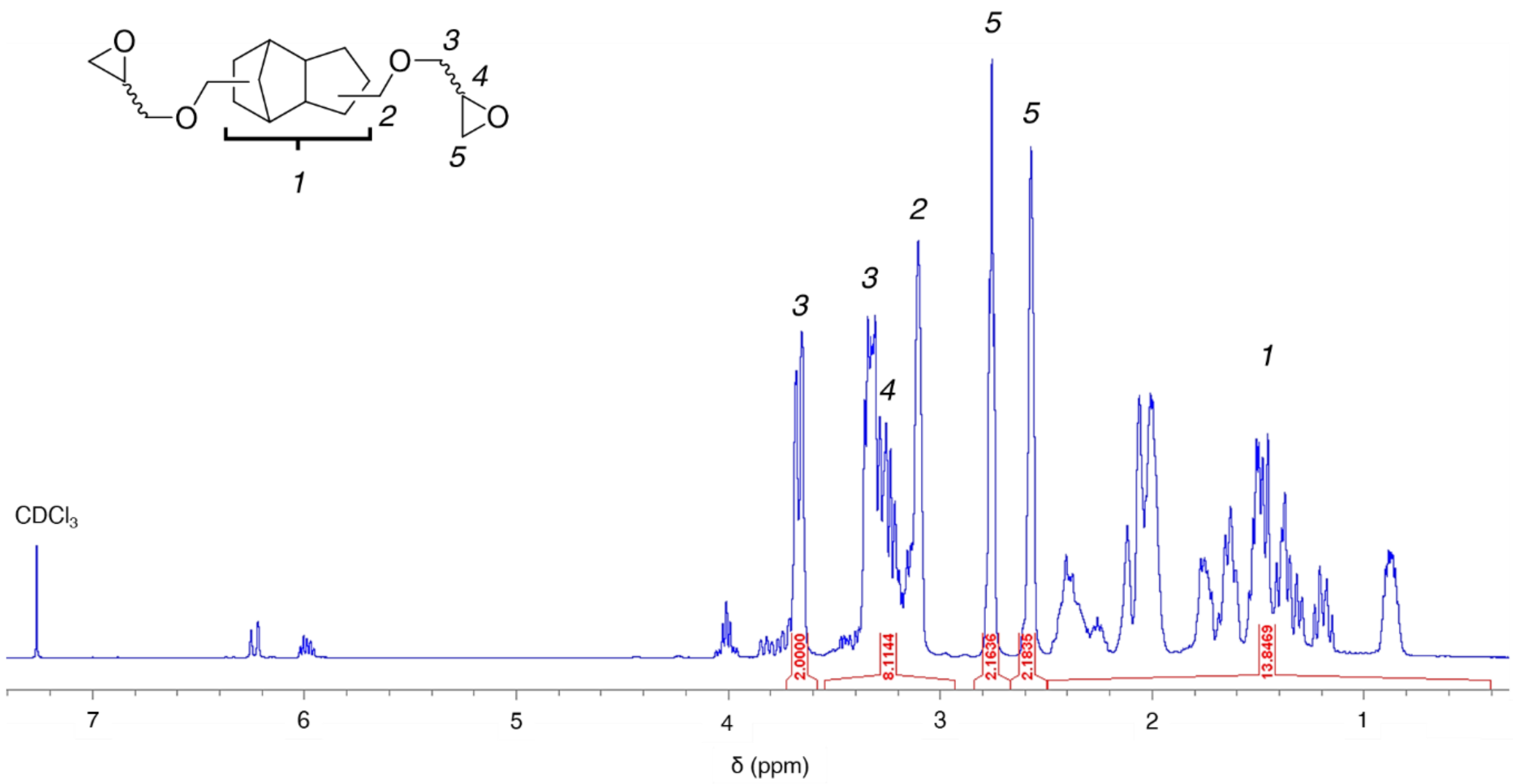


***Figure S10.*** *$^1$H-NMR spectrum of TCDDGE in $CDCl_3$ (400 MHz).*

## ISDGE

Isosorbide diglycidyl ether was prepared in two steps from isosorbide (**Figure S11**), as direct glycidylation of the sterically hindered alcohol groups with epichlorohydrin proved difficult.[4–6] In a first step, isosorbide is reacted with allyl bromide (distilled before use and dried over $MgSO_4$ under Ar) to yield isosorbide diallyl ether. Isosorbide (0.8211 mol, 120.00 g) and 5 mol% TBAB were dissolved in 2.5 equiv. (178 ml) allyl bromide in a two-necked flask equipped with a reflux condenser and stirred under Ar. Next, NaOH (pellets, 4 equiv.) was added, and the mixture was heated to 75 °C for 5 h (reflux), while monitoring the reaction progress *via* TLC. After cooling to rt, NaBr and NaOH were removed through filtration, and the solids were washed with dichloromethane. Next, the solvent and residual allyl bromide were removed under reduced pressure to yield a crude product. Finally, the diallyl ether was subjected to vacuum distillation to give a pure product in 74 % yield.

In a second step, the double bonds of isosorbide diallyl ether were oxidized using Oxone (2 $KHSO_5 \cdot KHSO_4 \cdot K_2SO_4$). In a three-necked flask, equipped with a mechanical stirrer and a reflux condenser, isosorbide diallyl ether (0.6055 mol, 137.00 g) was dissolved in acetone (6 equiv., 268.8 ml) and water (806.3 ml). After cooling the solution to 0 °C (ice bath), a mixture of Oxone (4.5 equiv.) and $NaHCO_3$ (9 equiv.) was added portion-wise over 5 h (10 portions). Then, the solution was allowed to heat to rt and was left stirring overnight. Subsequently, the mixture was filtered (solids washed with dichloromethane) and extracted with dichloromethane (2 x), ethyl acetate (2 x) and diethyl ether (2 x). The solvent was removed under vacuum, giving ISDGE in 86 % yield with 98 % purity.

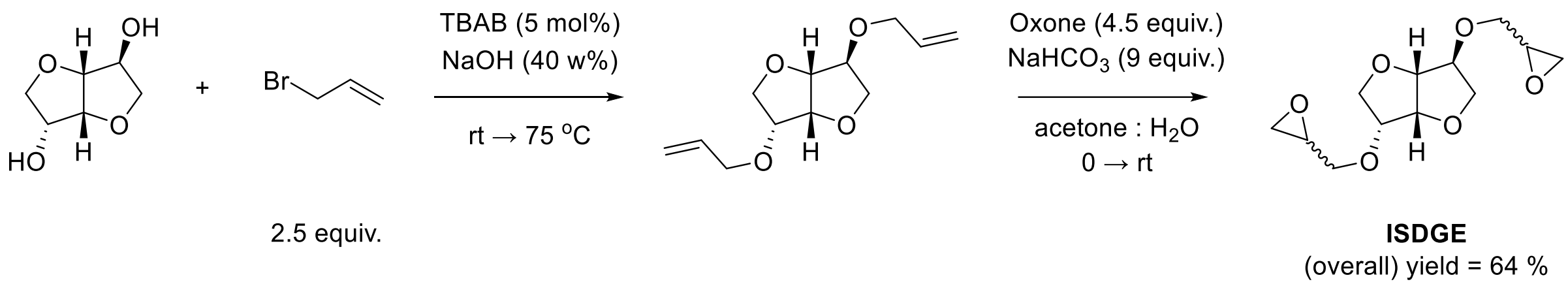


***Figure S11.*** *Diallylation of isosorbide and subsequent epoxidation to ISDGE.*

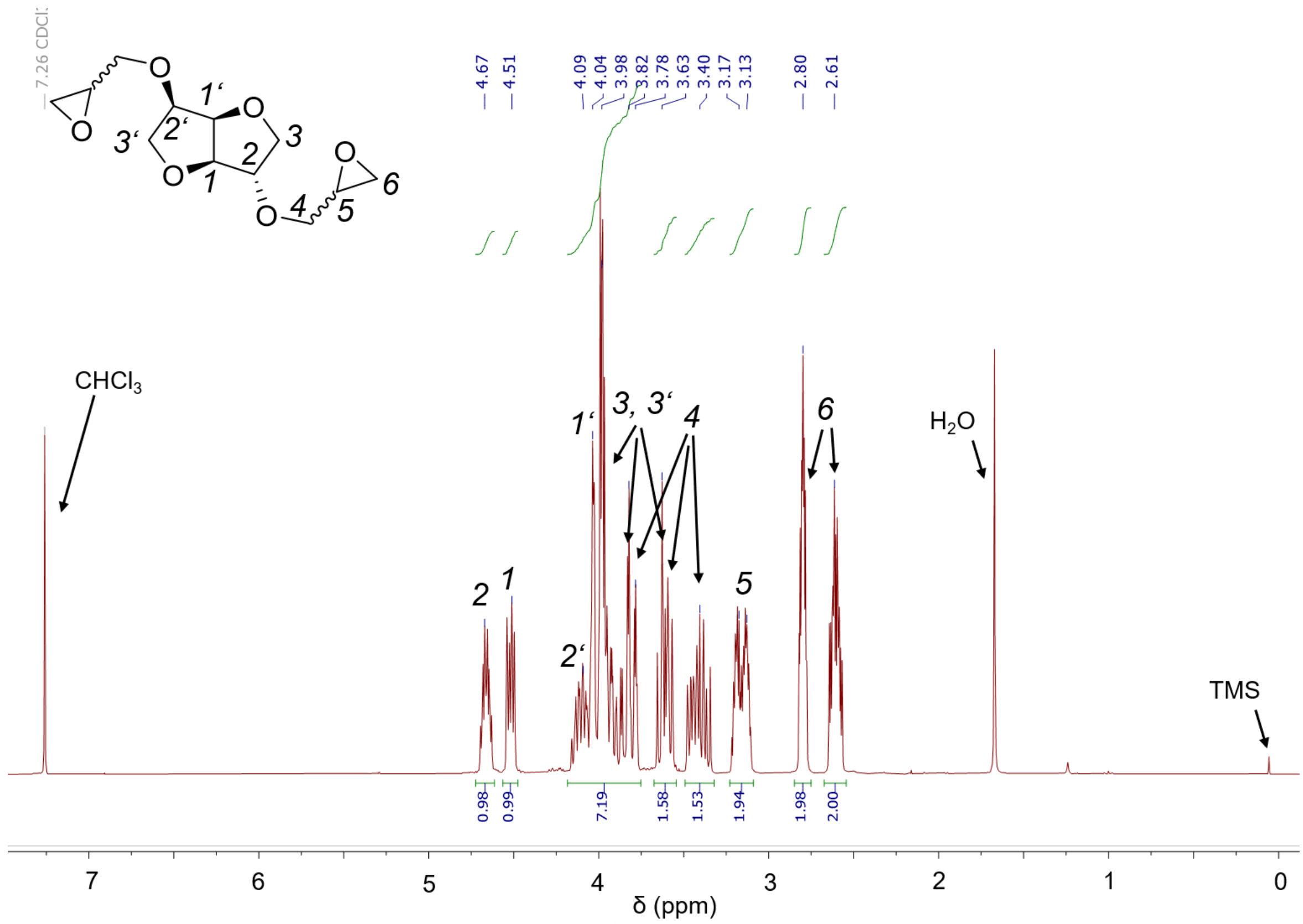

***Figure S12.*** *$^1H$-NMR spectrum of ISDGE in $CDCl_3$ (300 MHz).*

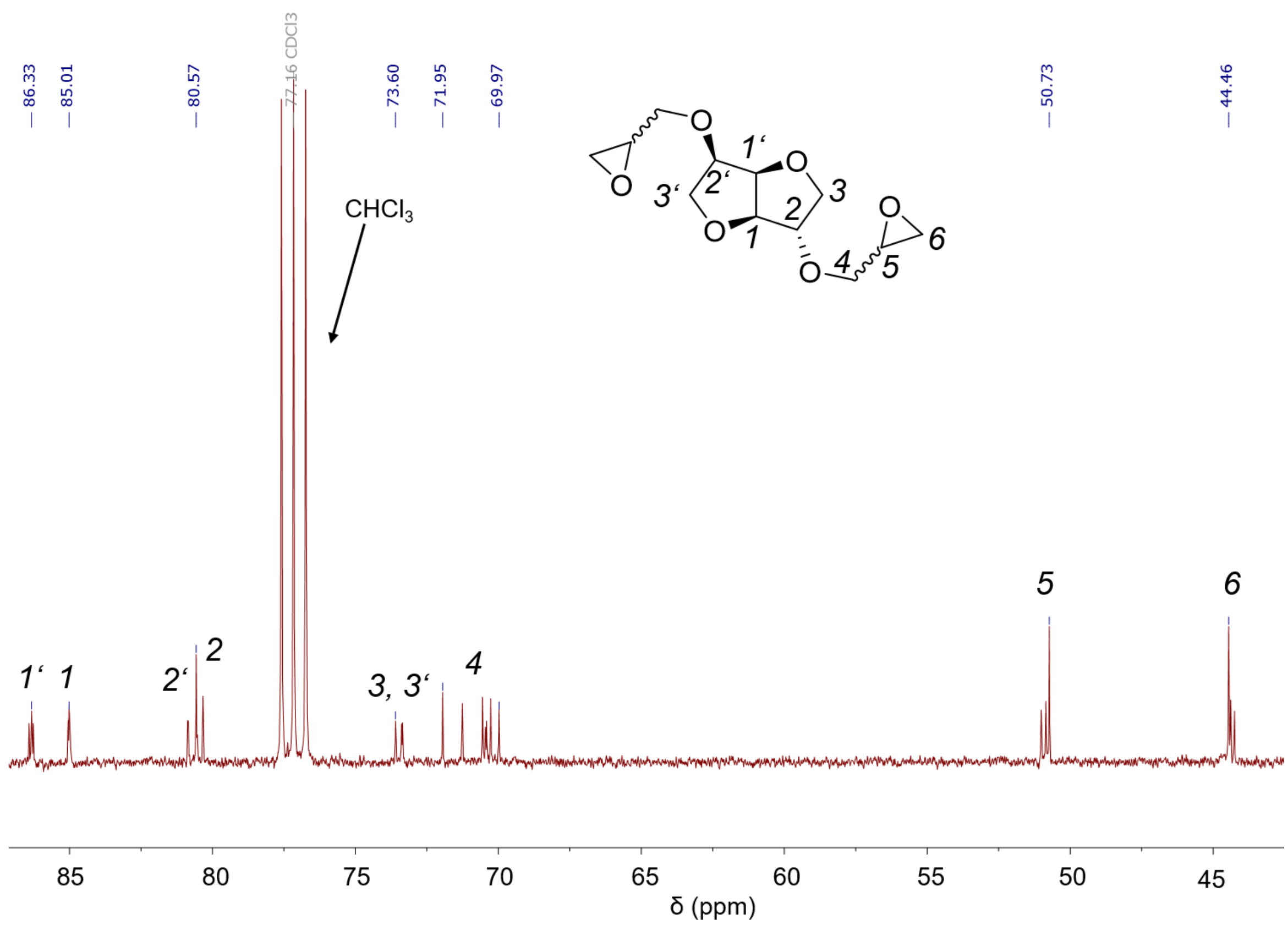

***Figure S13.*** *$^{13}C$-NMR spectrum of ISDGE in $CDCl_3$ (300 MHz).*

## TPADGE

Terephthalic acid diglycidyl ether is commercially available (95 %, Denacol Ex-711, Nagase). 10 g was dissolved in 100 ml dichloromethane, washed with water three times, dried over $MgSO_4$ and the solvent was removed under vacuum.

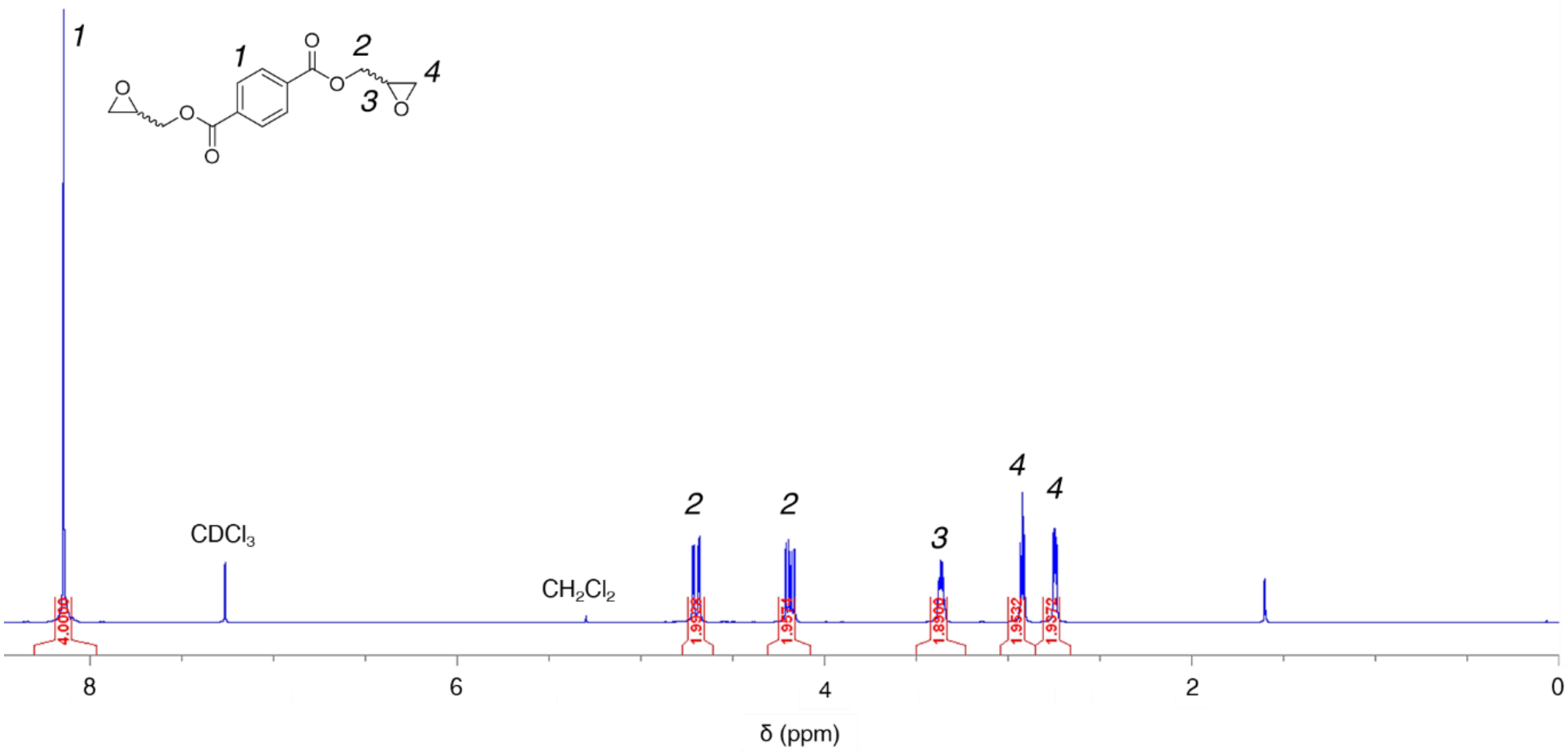

***Figure S14.*** *$^1$H-NMR spectrum of TPADGE in $CDCl_3$ (400 MHz).*[7]

## BDMDGE

1,4-benzenedimethanol diglycidyl ether was prepared by glycidylation of 1,4-benzenedimethanol following a procedure by Palmese *et al.* (**Figure S15**).[8] 0.2 mol 1,4-benzenedimethanol and 5 mol% tetrabutylammonium bromide were stirred at 50 °C for 1 h in 500 ml of an aqueous 40 w% NaOH solution. In a next step, the mixture was cooled to 0 °C (ice bath) and epichlorohydrin (8 equiv.) was added dropwise over 30 min. The reaction mixture was then left to stir for 21 h at rt, before pouring it into ice-cold water (250 ml) and extraction with diethyl ether (6 x 200 ml). The combined organic phases were then washed with distilled water until a neutral *pH* was attained and then dried over $MgSO_4$. The solvent, as well as most of the residual epichlorohydrin, was removed under reduced pressure (rotary evaporator). Finally, the crude product was purified by column chromatography over activated alumina using diethyl ether and ethyl acetate as eluents to give BDMDGE in 74 % yield. Alternatively, vacuum distillation could be used to purify the monomer.

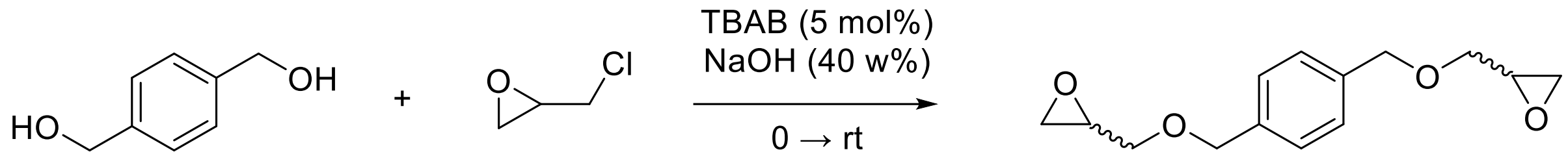


8 equiv.

**BDMDGE**
yield = 74 %

***Figure S15.*** *Diglycidylation of 1,4-benzenedimethanol.*

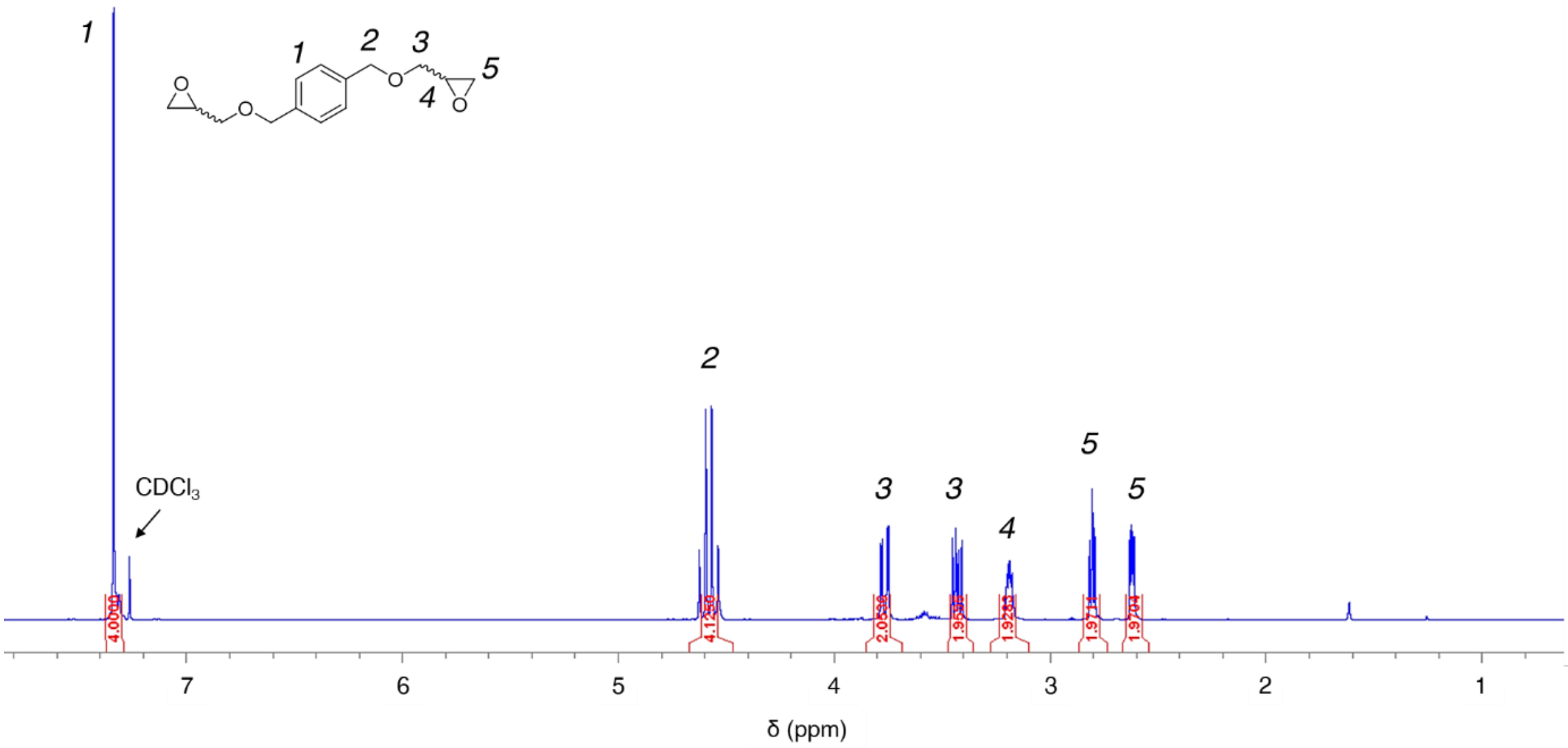


***Figure S16.*** *$^1$H-NMR spectrum of BDMDGE in $CDCl_3$ (400 MHz).*[9]

## DMHBE

Dimethylhydantoin bisepoxide is commercially available[10] (BLD pharm, 90 %) but was purified *via* column chromatography ($SiO_2$, sample dissolved in acetone/dichloromethane, eluent: EtOAc).

## DGI

Diglycidyl imidazolidinone was synthesized from 2-imidazolidinone through diglycidylation with epichlorohydrin, following a modified literature procedure (**Figure S17**).[11] 2-imidazolidinone (0.0092 mol, 0.800 g), freshly ground NaOH powder (2.5 equiv.) and 8 ml dimethylsulfoxide (DMSO) were mixed for 30 min under Ar at rt in a 2-neck flask equipped with a reflux condenser. Then, epichlorohydrin (6 equiv.) was added dropwise, and the mixture was left to stir for 30 min at rt and then stepwise heated to 70 °C, while monitoring the reaction progress by TLC. Next, dichloromethane (50 ml) and water (50 ml) were added, and the aqueous phase was extracted twice with dichloromethane (50 ml) The combined organic phases were washed with brine and dried over $MgSO_4$. After solvent removal under vacuum, a colourless, solid

crude product was obtained, which was further purified through column chromatography (ethyl acetate : ethanol 10 : 1) to yield 57 % of pure product.

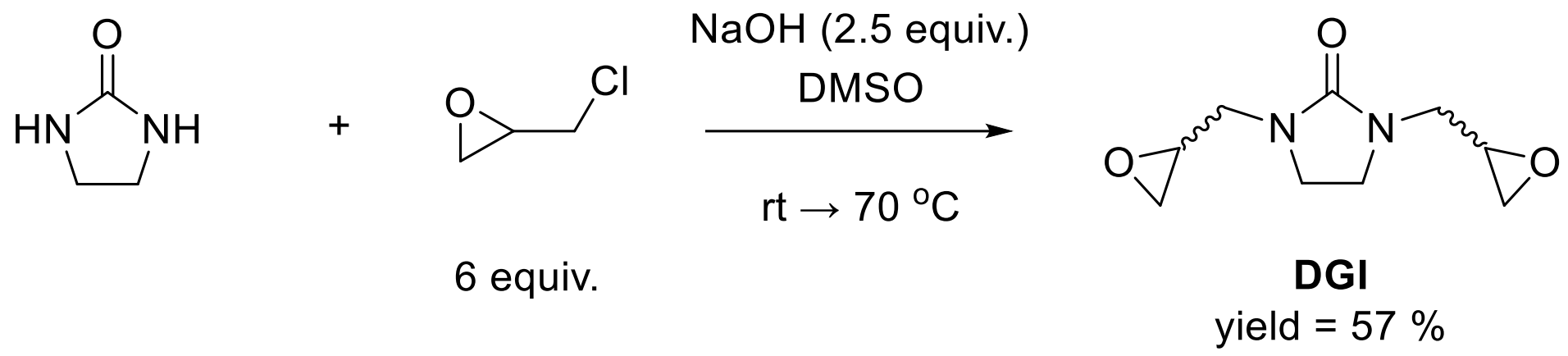


***Figure S17.*** *Diglycidylation of 2-imidazolidinone.*

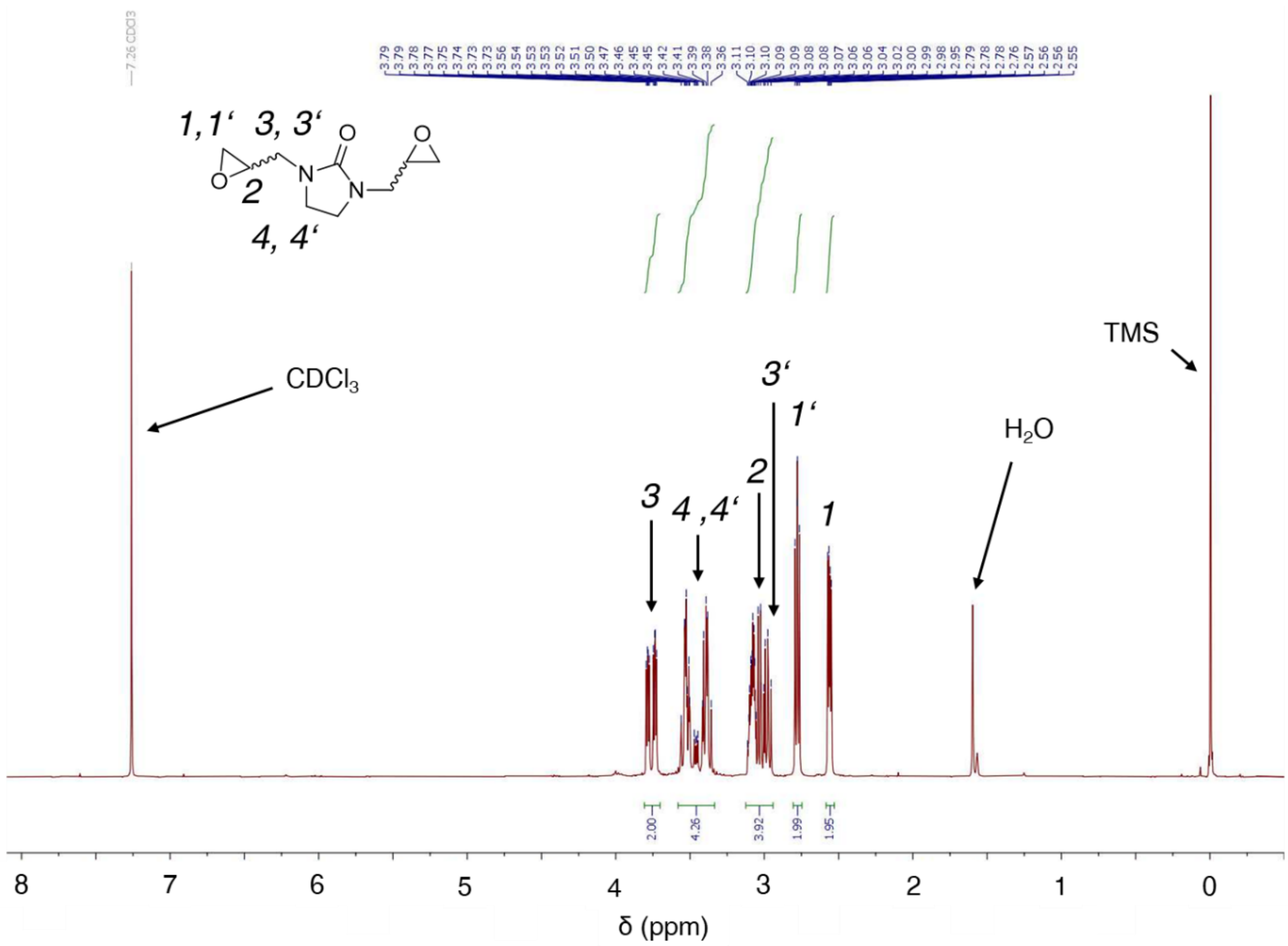


***Figure S18.*** *$^1$H-NMR spectrum of DGI in $CDCl_3$ (400 MHz).*

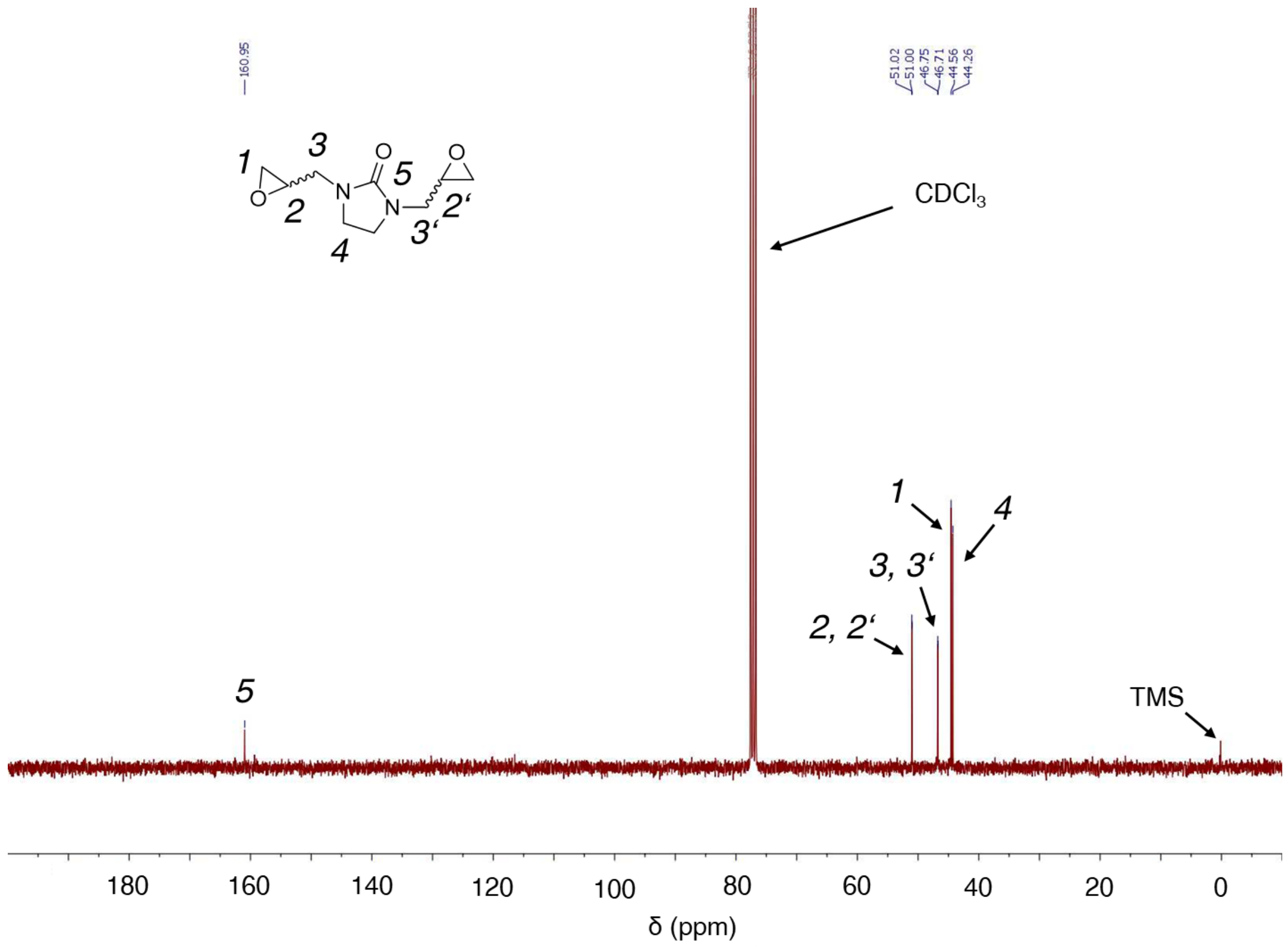


***Figure S19.*** *$^{13}$C-NMR spectrum of DGI in $CDCl_3$ (400 MHz).*

## DGBI

1,3-bis(oxiran-2-ylmethyl)-1,3-dihydro-2*H*-benzo[*d*]imidazol-2-one (DGBI) was synthesized following the same method as DGI (**Figure S20**).[11] In a two-necked flask equipped with a reflux condenser, 1,3-dihydro-2*H*-benzo[*d*]imidazole-2-one (0.0092 mol, 1.23 g) and freshly ground NaOH (2.5 equiv.) were dissolved in DMSO (8 ml) under Ar at rt and stirred for 20 min. Then, epichlorohydrin (6 equiv.) was added dropwise, and the mixture was stirred for 10 min before heating stepwise to 70 °C. The reaction progress was monitored by TLC and at the end of the reaction, dichloromethane (50 ml) and water (50 ml) were added. The aqueous phase was extracted with dichloromethane (2 x 50 ml) and the combined organic phases were washed with brine and dried over $MgSO_4$. After solvent removal under vacuum, a white, solid crude product was obtained, which was purified by column chromatography (ethyl acetate : ethanol 10 : 1) to yield 52 % of pure product.

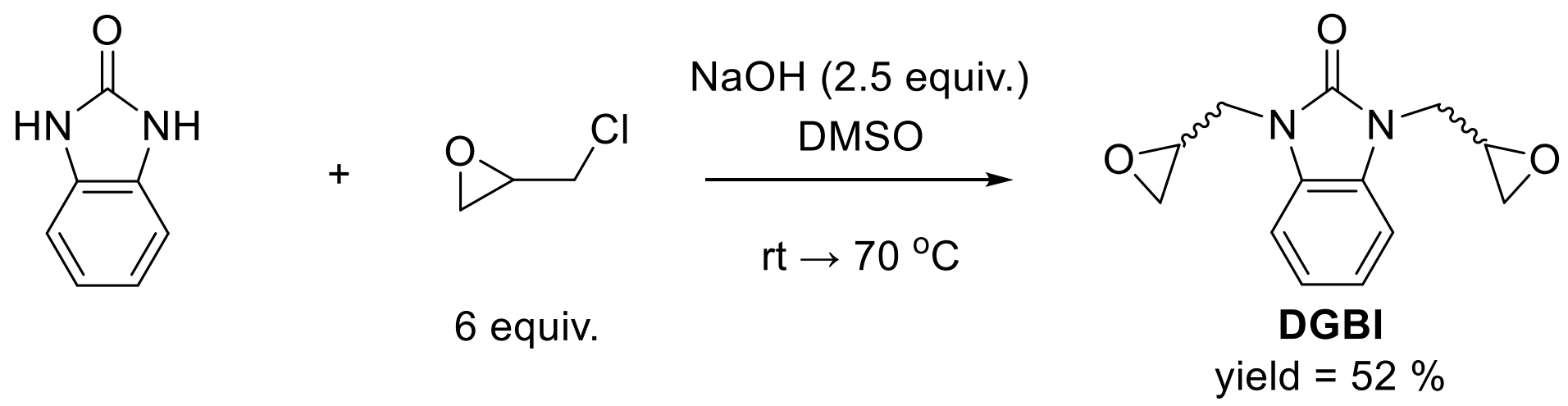


***Figure S20.*** *Diglycidylation of 1,3-dihydro-2*H*-benzo[*d*]imidazole-2-one.*

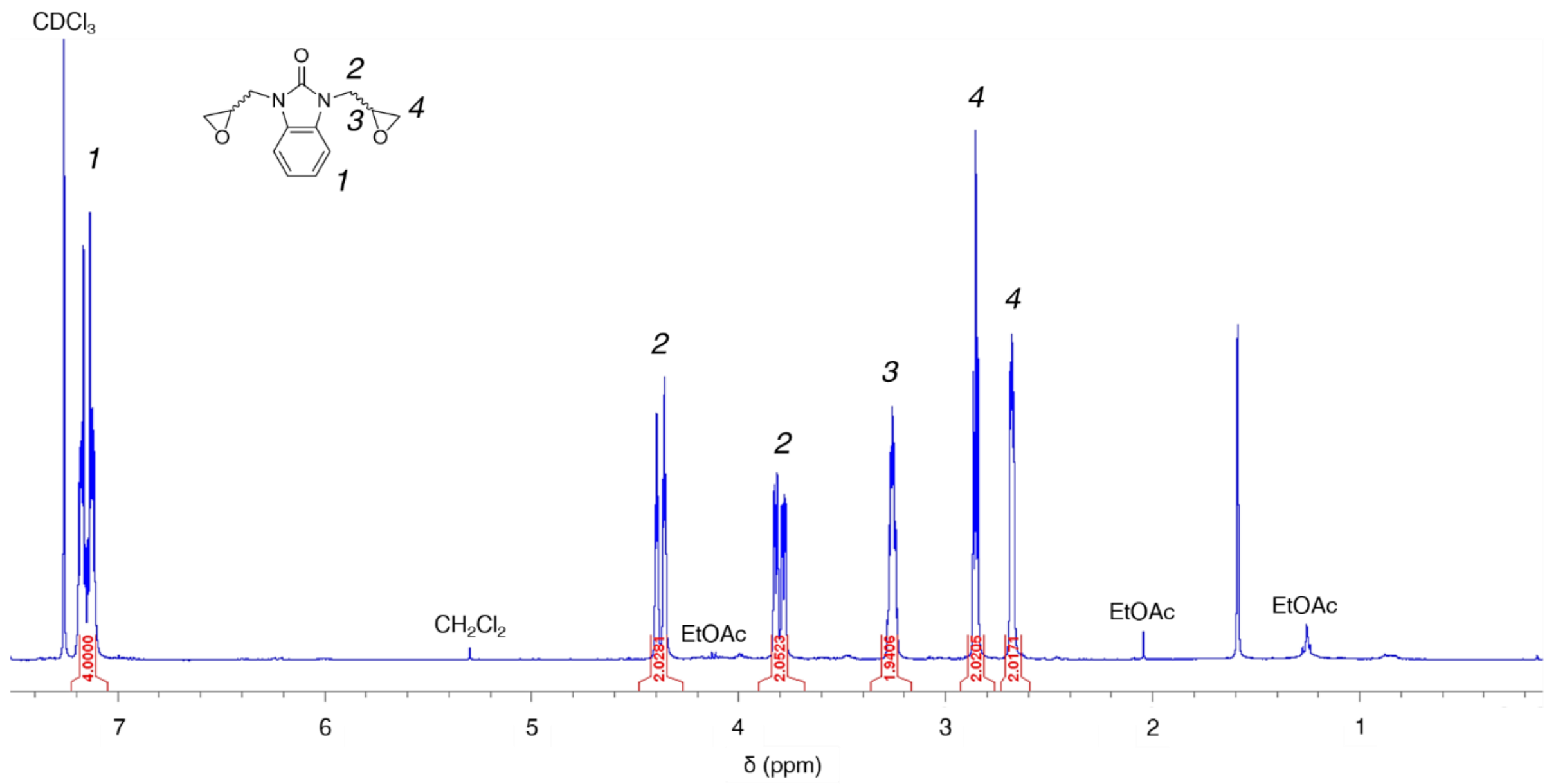


***Figure S21.*** *$^{1}$H-NMR spectrum of DGBI in $CDCl_3$ (400 MHz).*

## Polymer synthesis

### BADGE-derived polyoxazolidinone

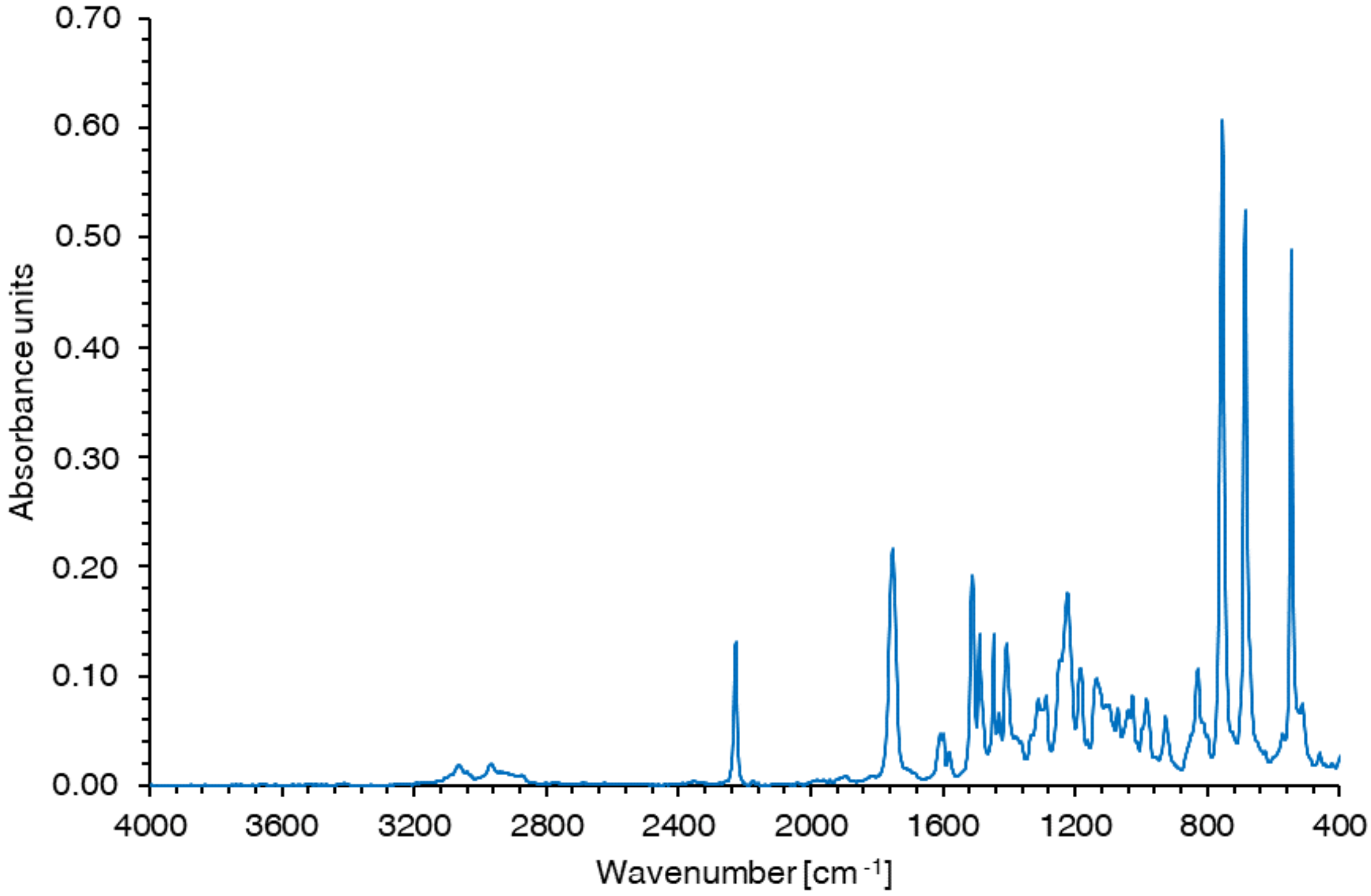


***Figure S22.*** *FTIR-spectrum of BADGE-based polyoxazolidinone.*

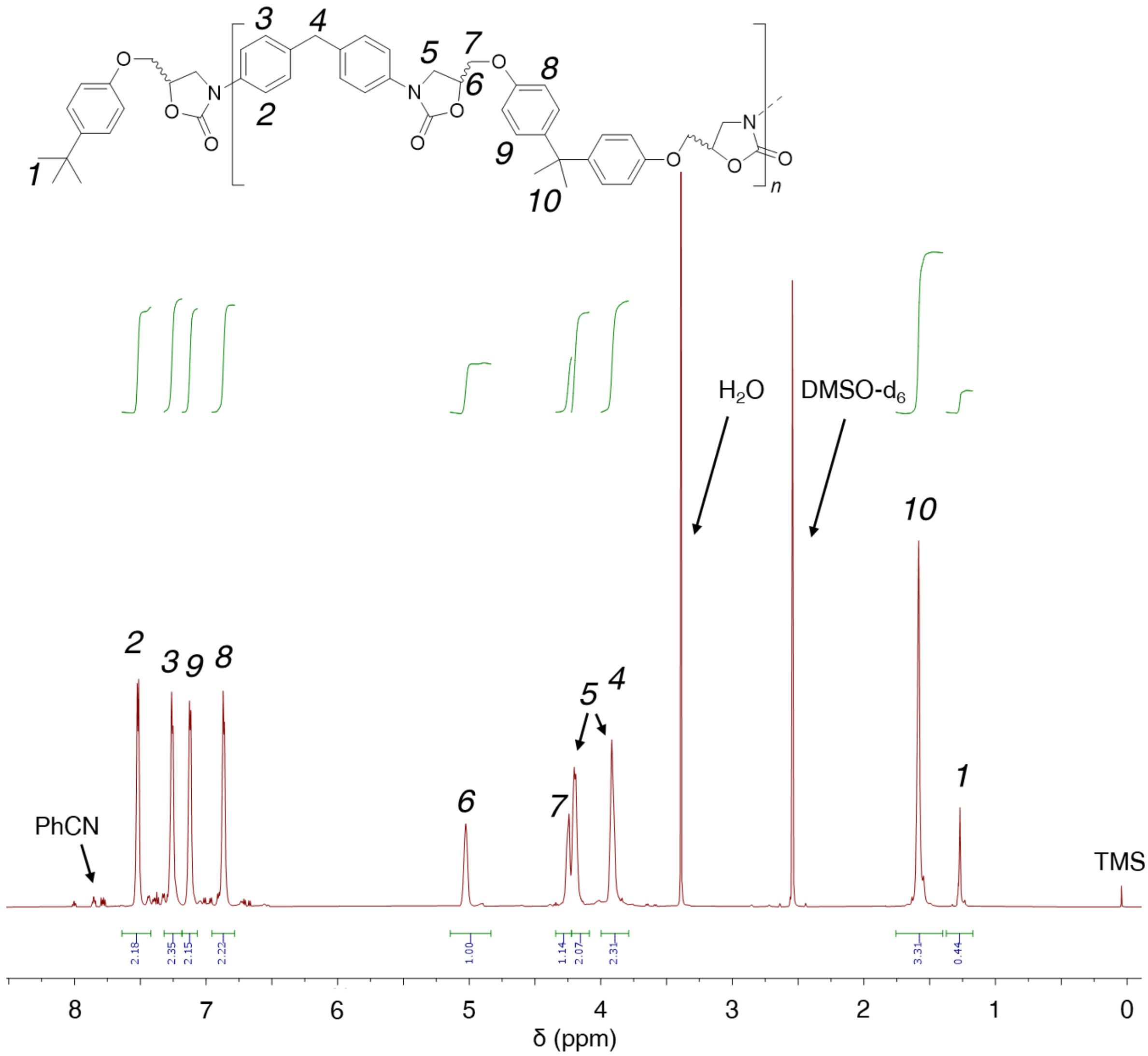


***Figure S23.*** *$^{1}$H-NMR spectrum of the BADGE-based polyoxazolidinone in DMSO-d$_6$ (700 MHz).*

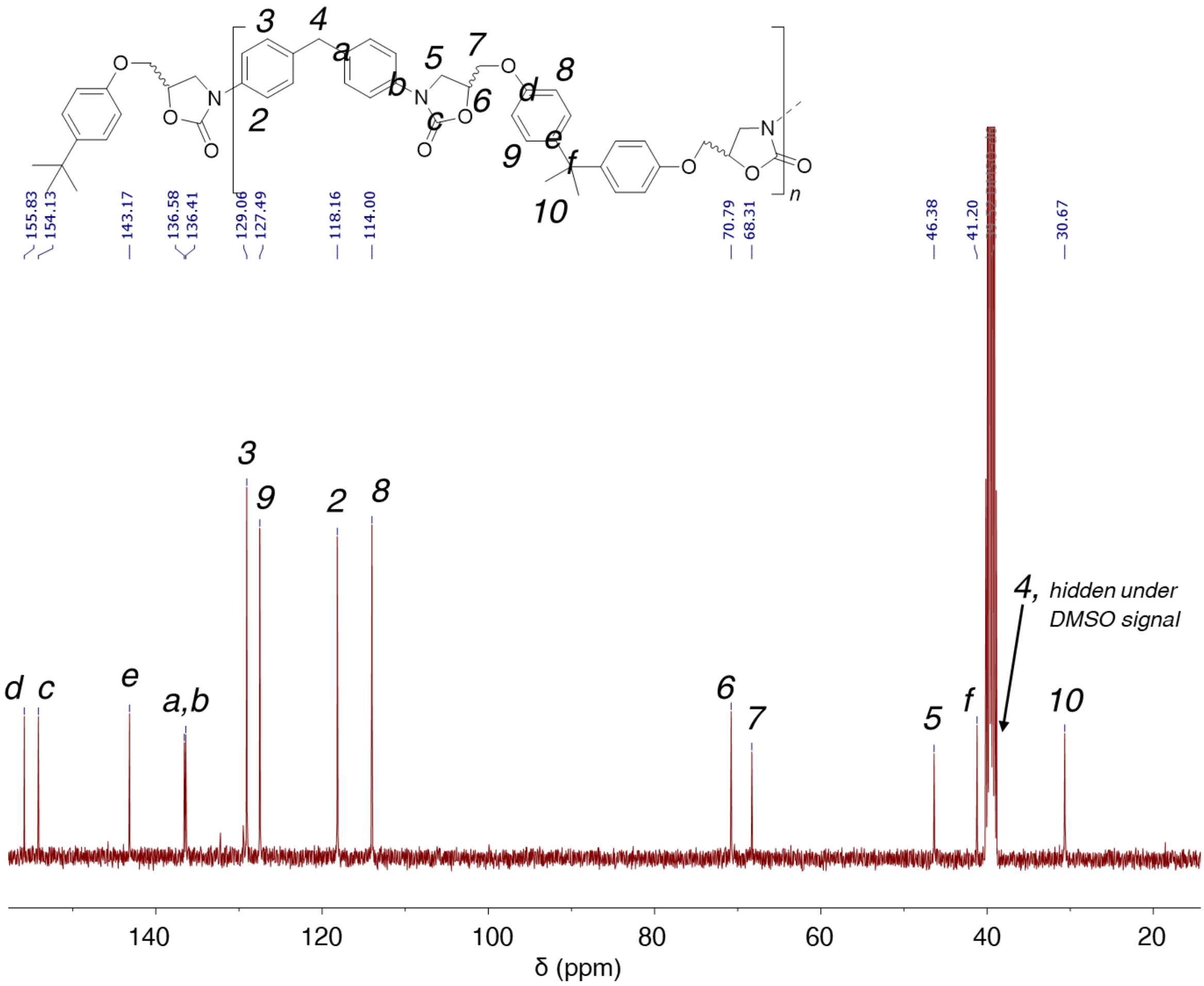


***Figure S24.*** *$^{13}$C-NMR spectrum of the BADGE-based polyoxazolidinone in DMSO-d₆ (300 MHz).*

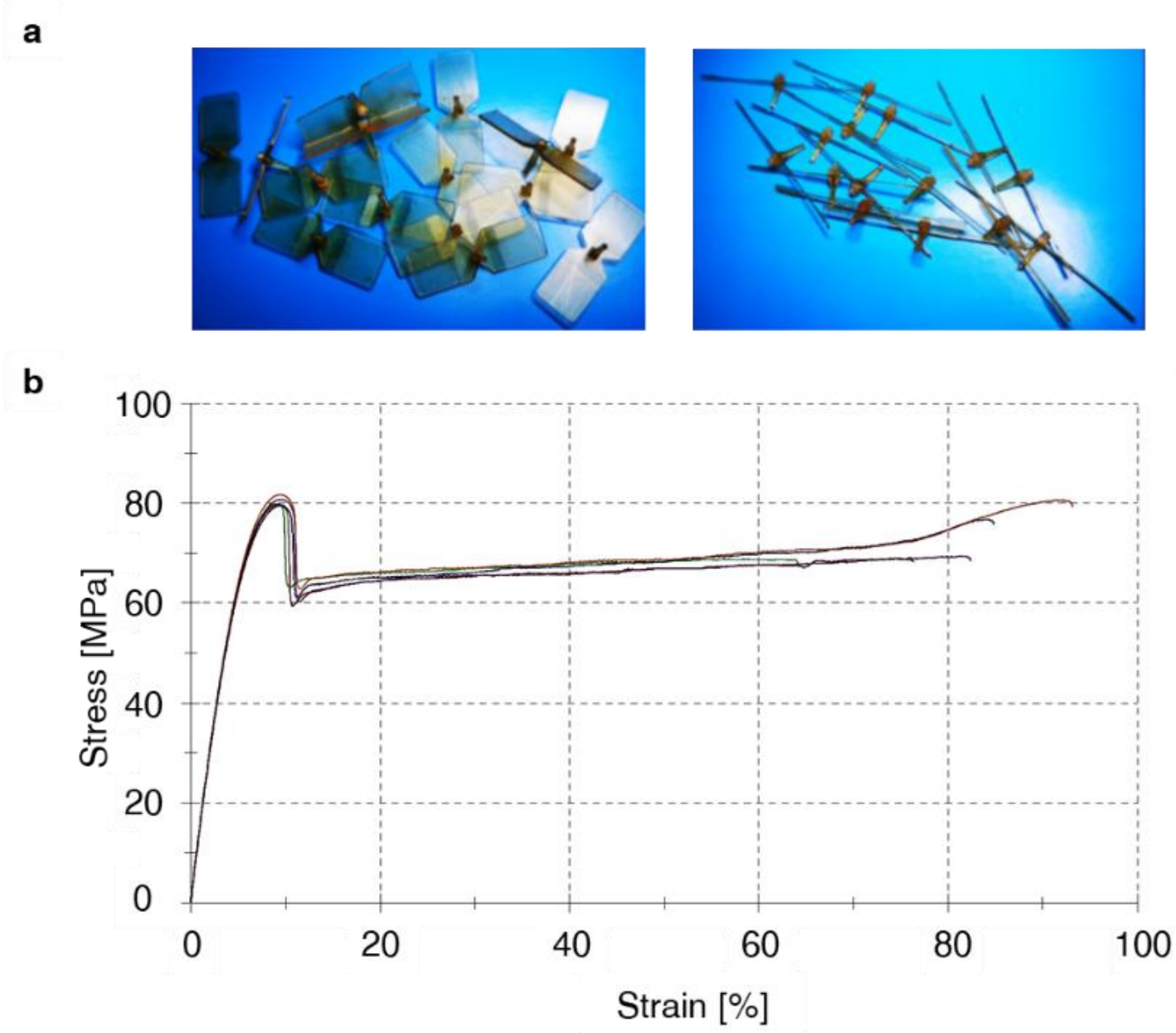


***Figure S25.*** *(a) Test specimen obtained by micro-injection moulding at KUZ Leipzig. (b) Stress-strain curve of polyoxazolidinone prepared with 4,4'-MDI and BADGE.*

## MDIDGE-derived polyoxazolidinone

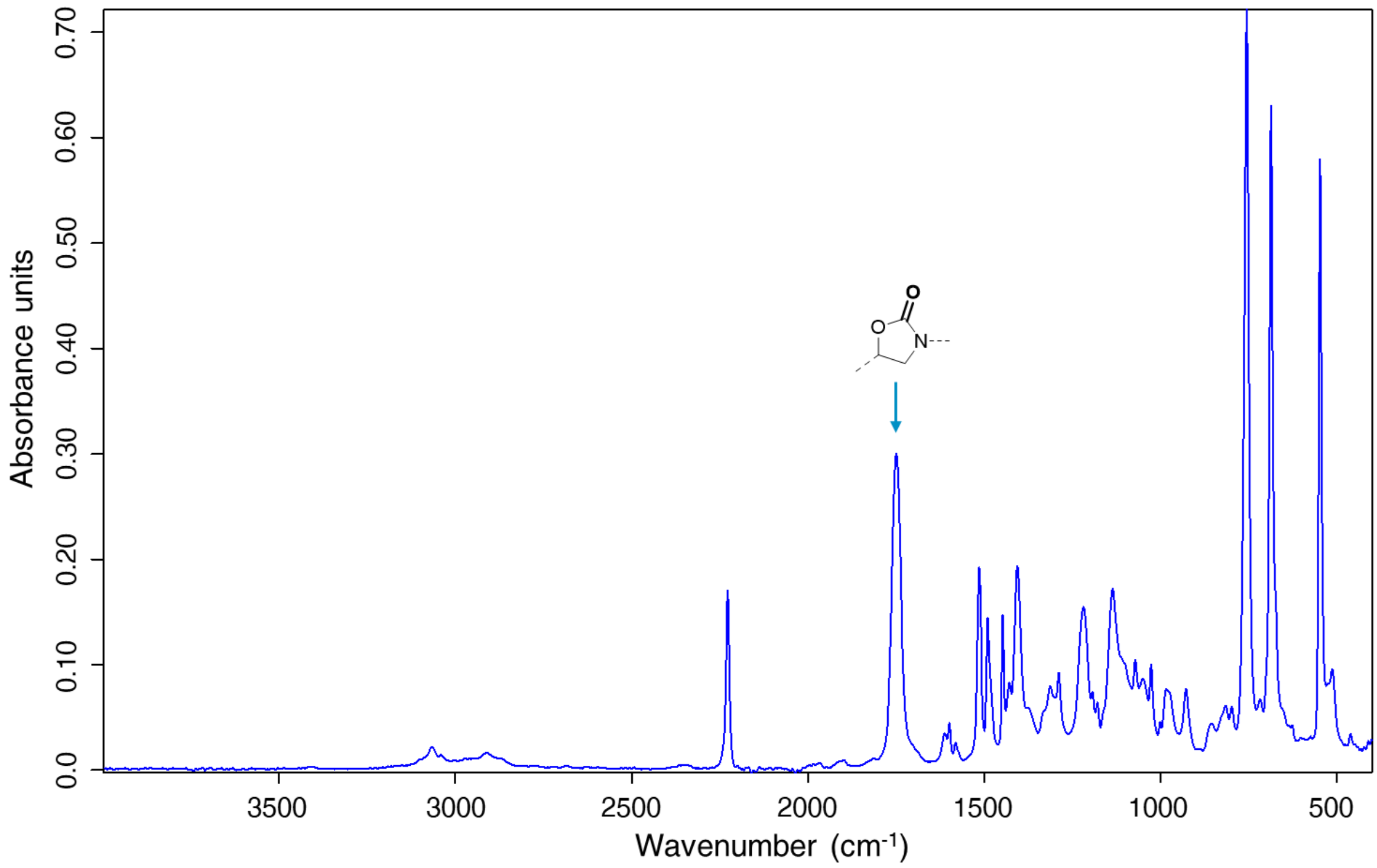


***Figure S26.*** *FTIR-spectrum of MDIDGE-based polyoxazolidinone at the end of the reaction.*

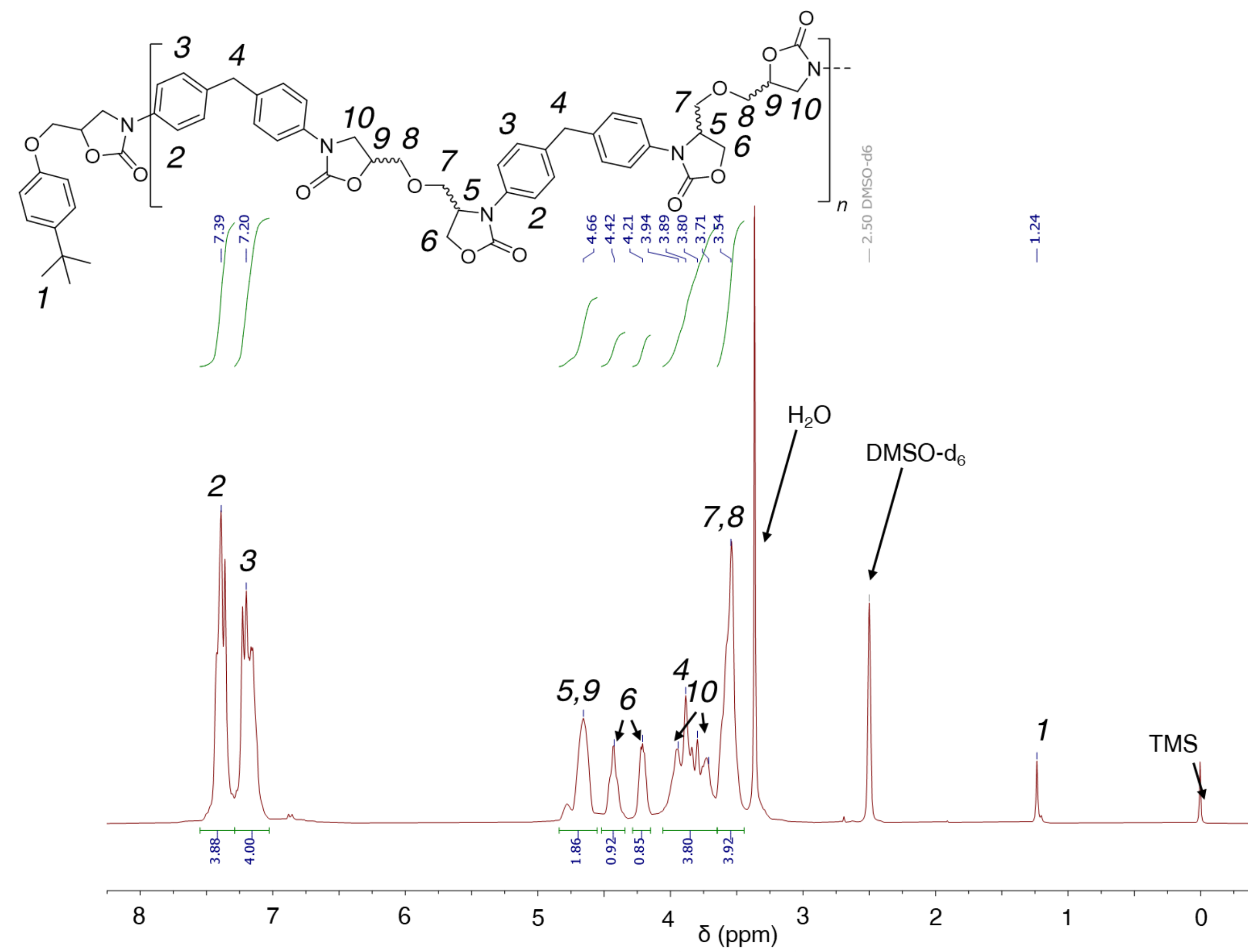


***Figure S27.*** *$^{1}$H-NMR spectrum of the MDIDGE-based polyoxazolidinone in DMSO-d₆ (300 MHz).*

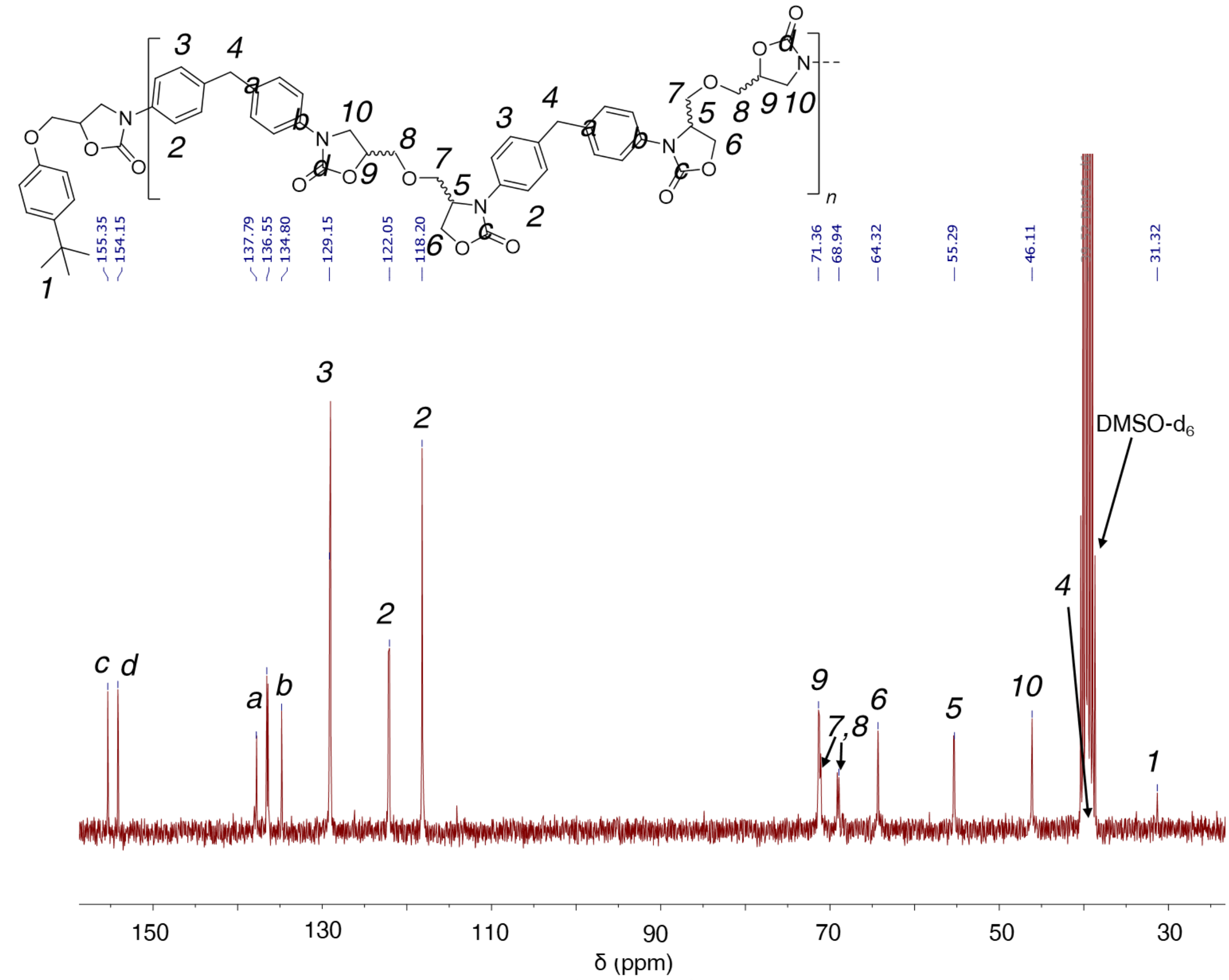


***Figure S28.*** *$^{1}$H-NMR spectrum of the MDIDGE-based polyoxazolidinone in DMSO-$d_6$ (300 MHz).*

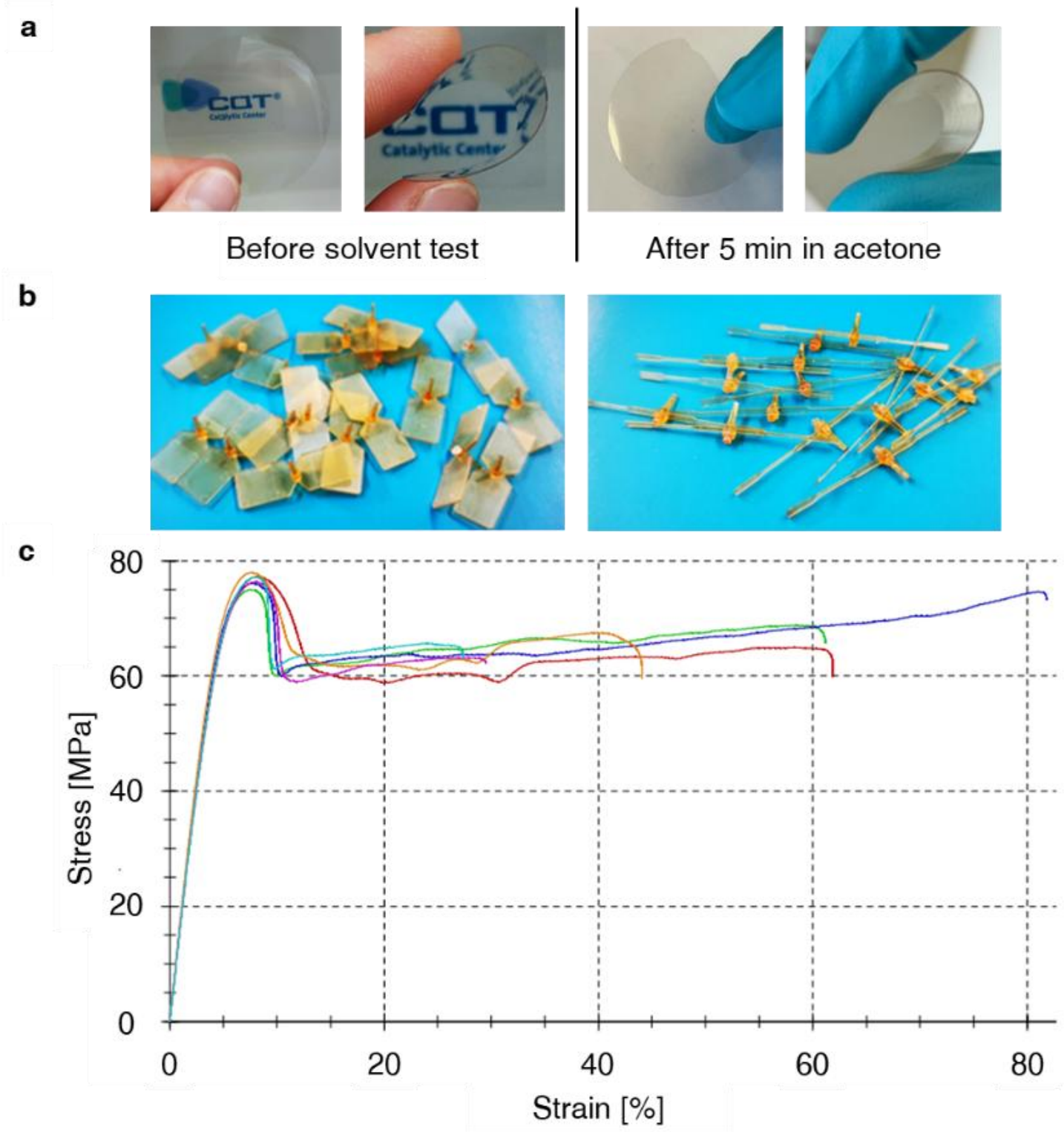


***Figure S29.*** *(a) Flexible, transparent plates from polyoxazolidinone with MDIDGE as bisepoxide were obtained in a thermal press at 230 °C and 10 kN for 30 s. In a preliminary solvent resistance test, the plate was dipped in acetone for 5 min and optically examined afterwards. (b) Test specimen obtained by micro-injection moulding at KUZ Leipzig. (c) Stress-strain curve of polyoxazolidinone prepared with 4,4'-MDI and MDIDGE.*

### DGE-derived polyoxazolidinone

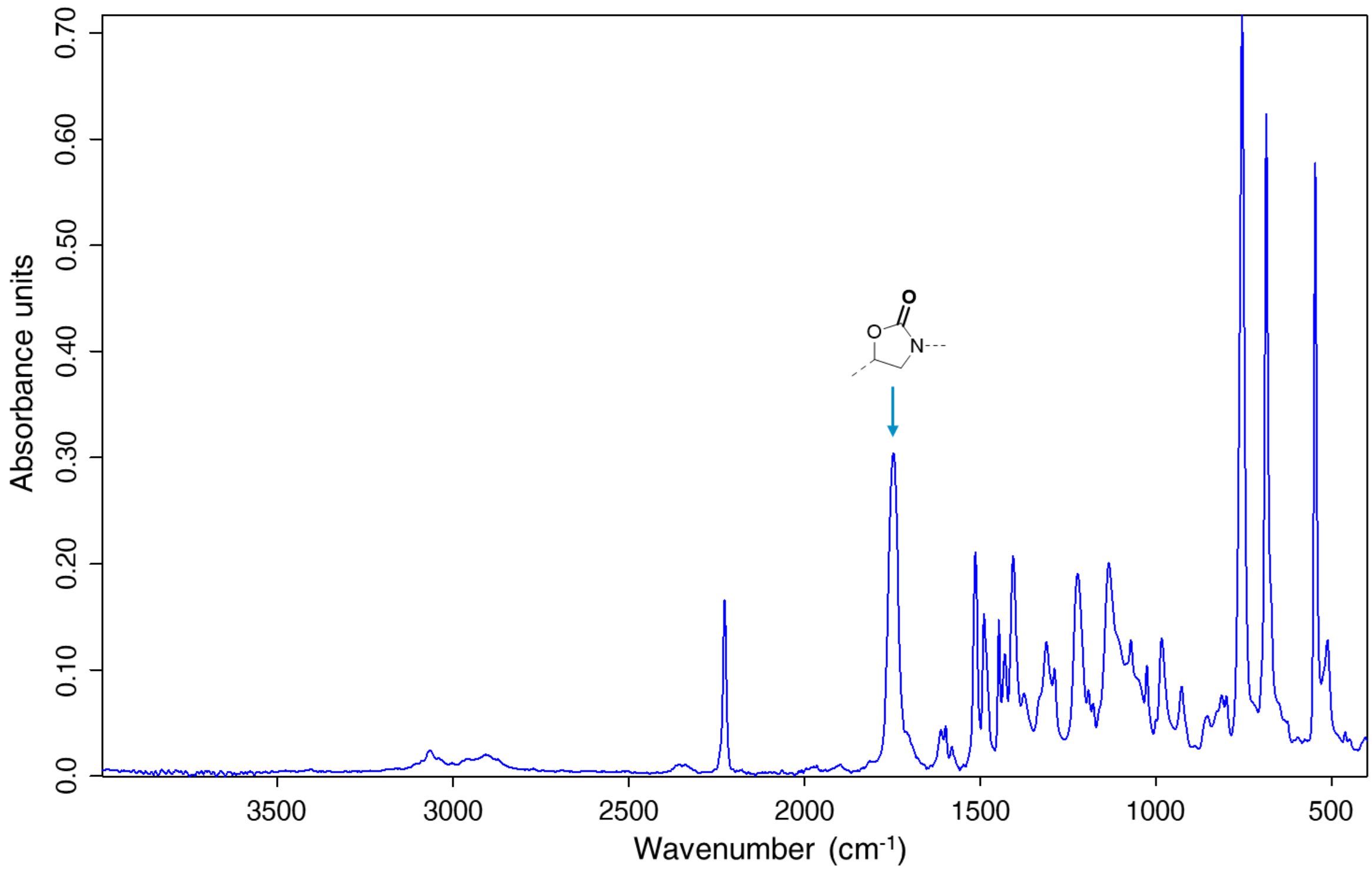


***Figure S30.*** *FTIR-spectrum of DGE-based polyoxazolidinone at the end of the reaction.*

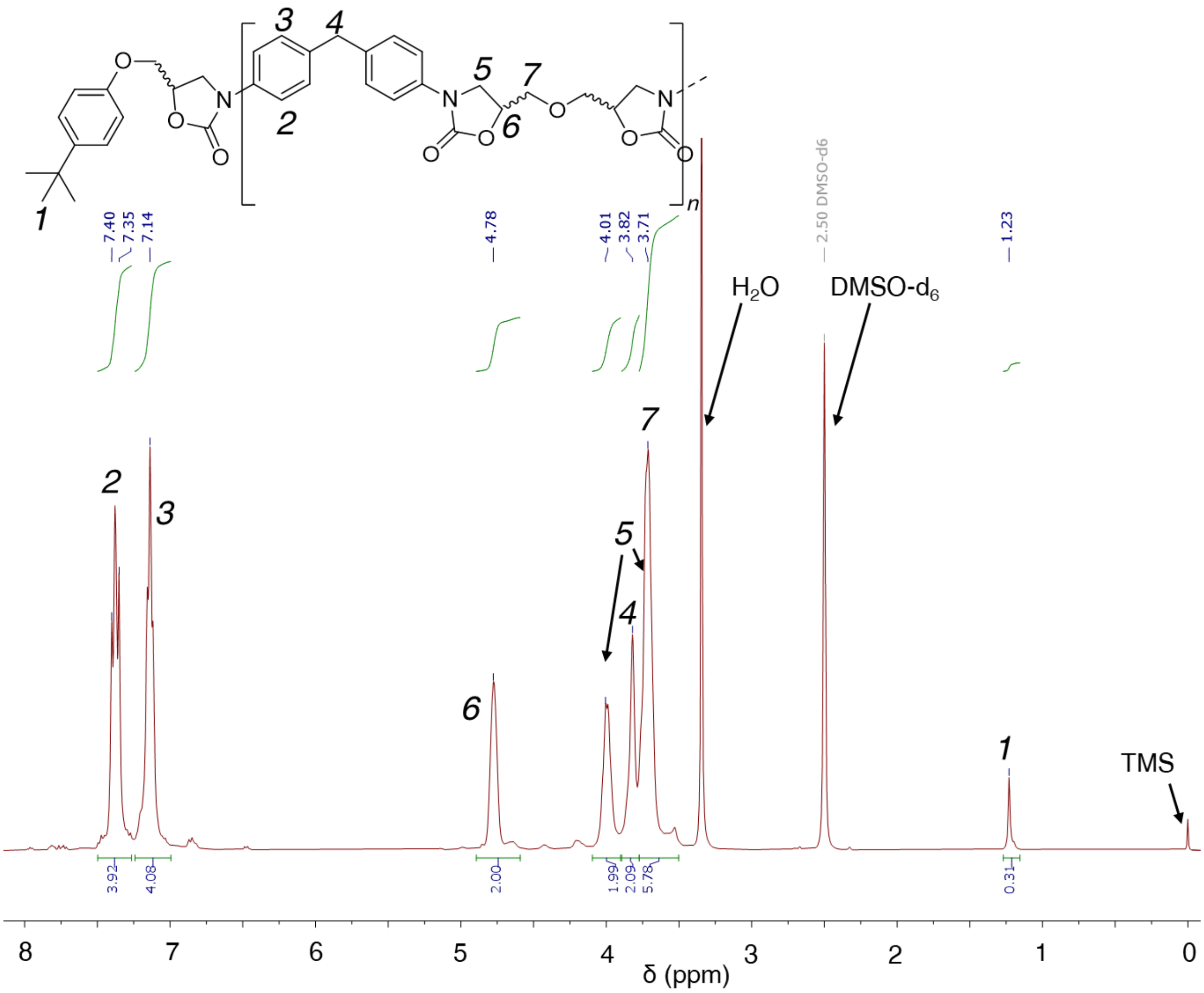


***Figure S31.*** *$^{1}$H-NMR spectrum of the DGE-based polyoxazolidinone in DMSO-d$_6$ (300 MHz).*

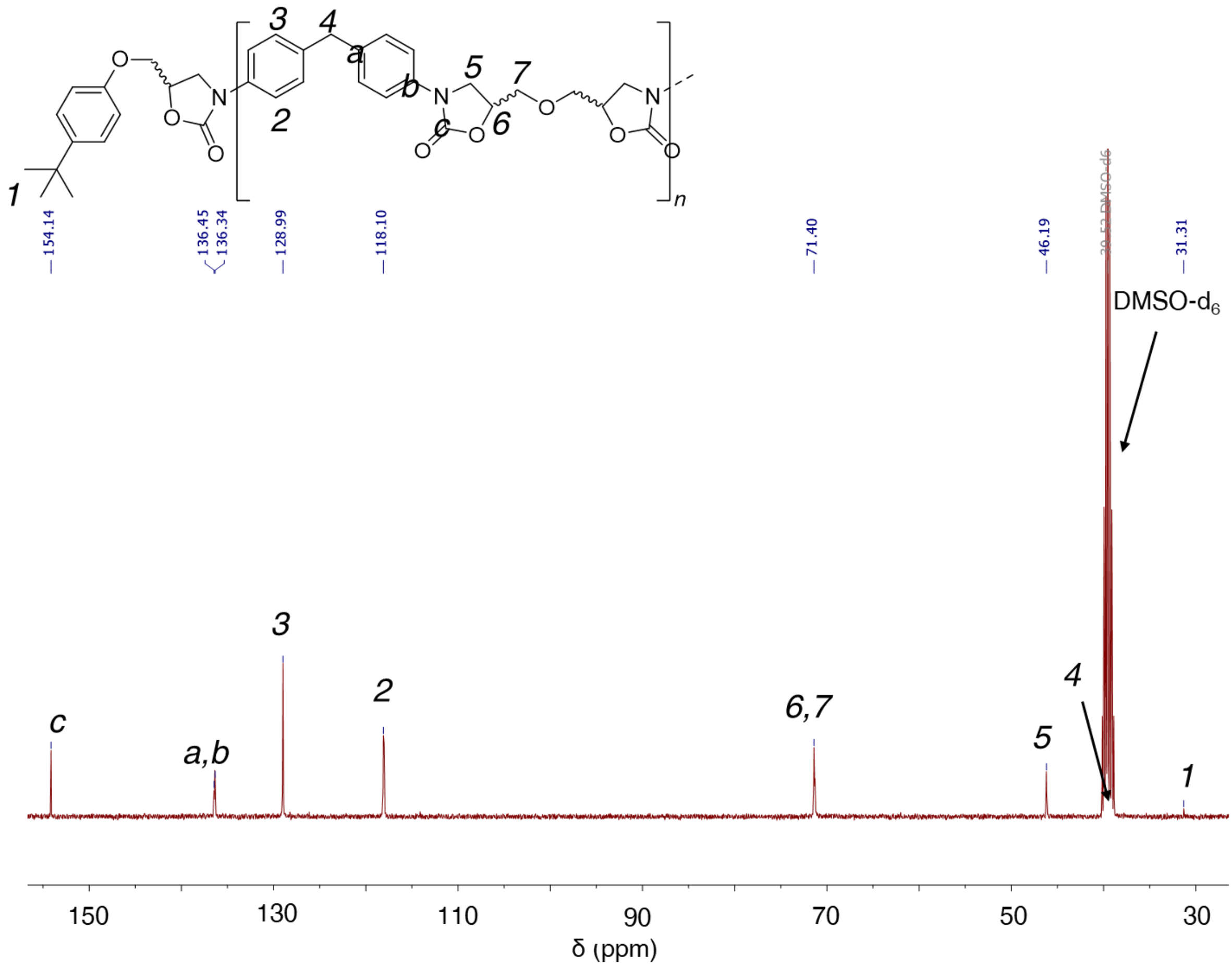


***Figure S32.*** *$^{13}$C-NMR spectrum of the DGE-based polyoxazolidinone in DMSO-d6 (300 MHz).*

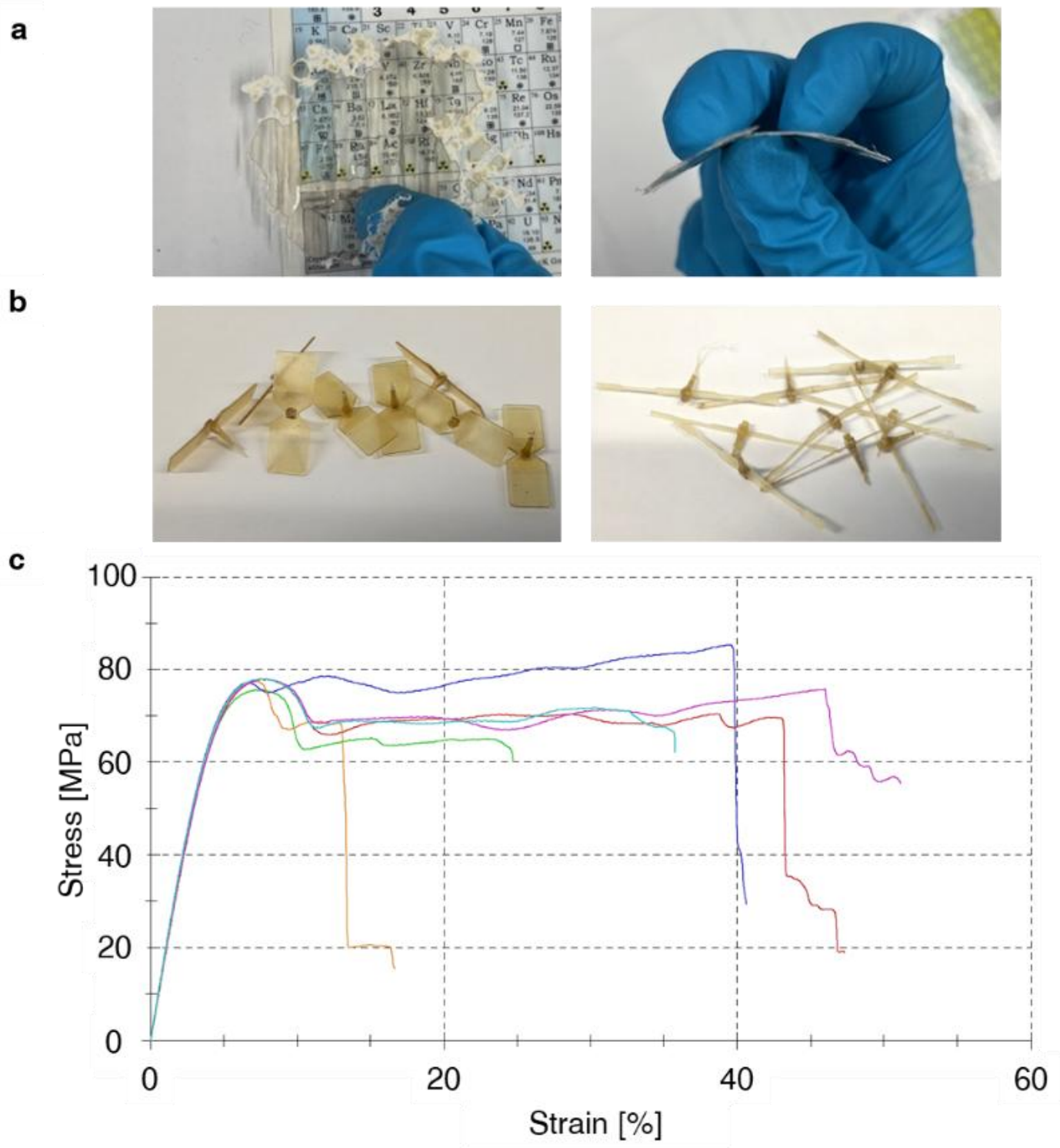


***Figure S33.*** *(a) Transparent, somewhat flexible plates from polyoxazolidinone with DGE as bisepoxide were obtained in a thermal press at 170 °C and 15 kN for 60 s. (b) Test specimen obtained by micro-injection moulding at KUZ Leipzig. (c) Stress-strain curve of polyoxazolidinone prepared with 4,4'-MDI and DGE.*

**CBDGE-based polyoxazolidinone**

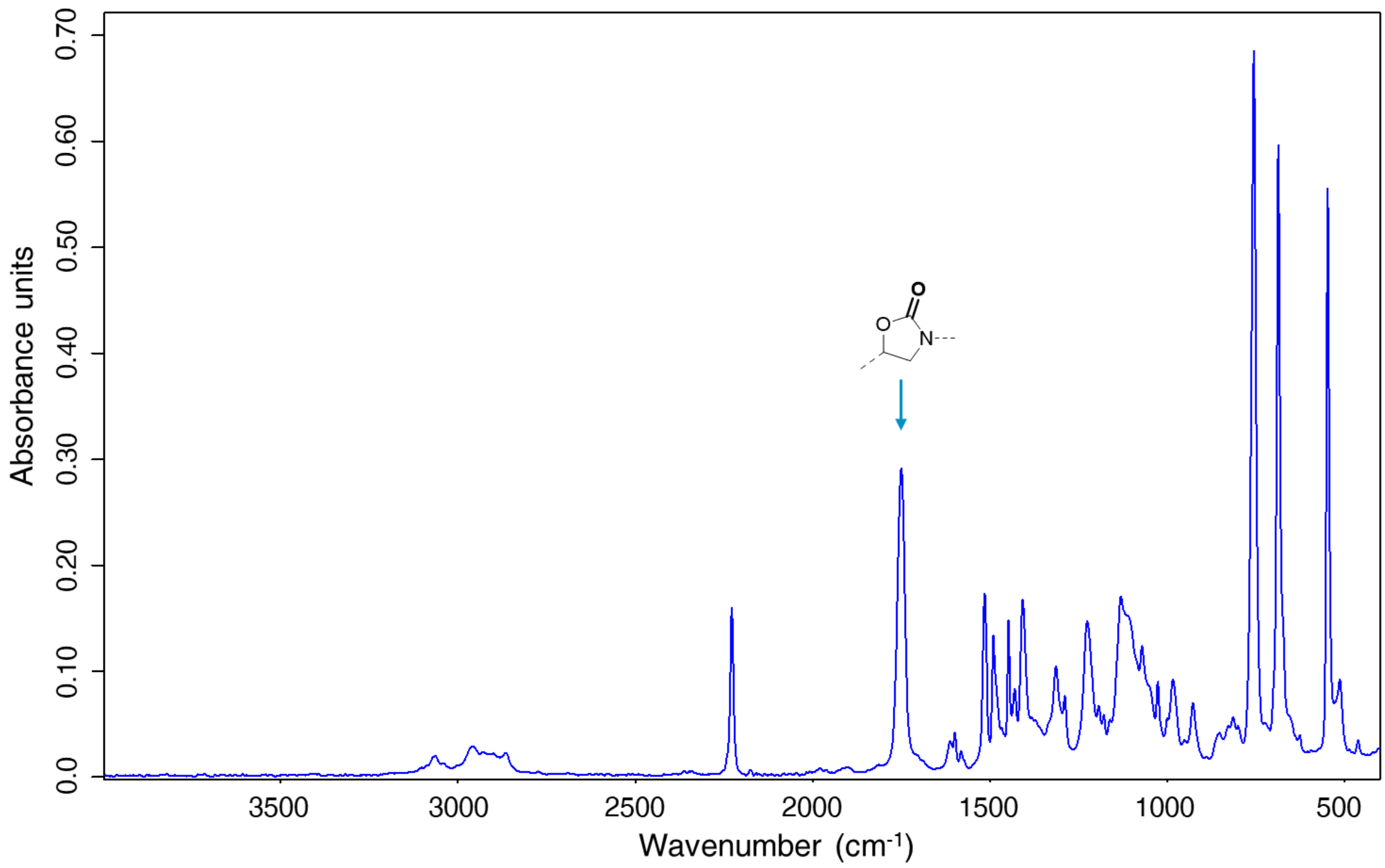


***Figure S34.*** *FTIR-spectrum of CBDGE-based polyoxazolidinone at the end of the reaction.*

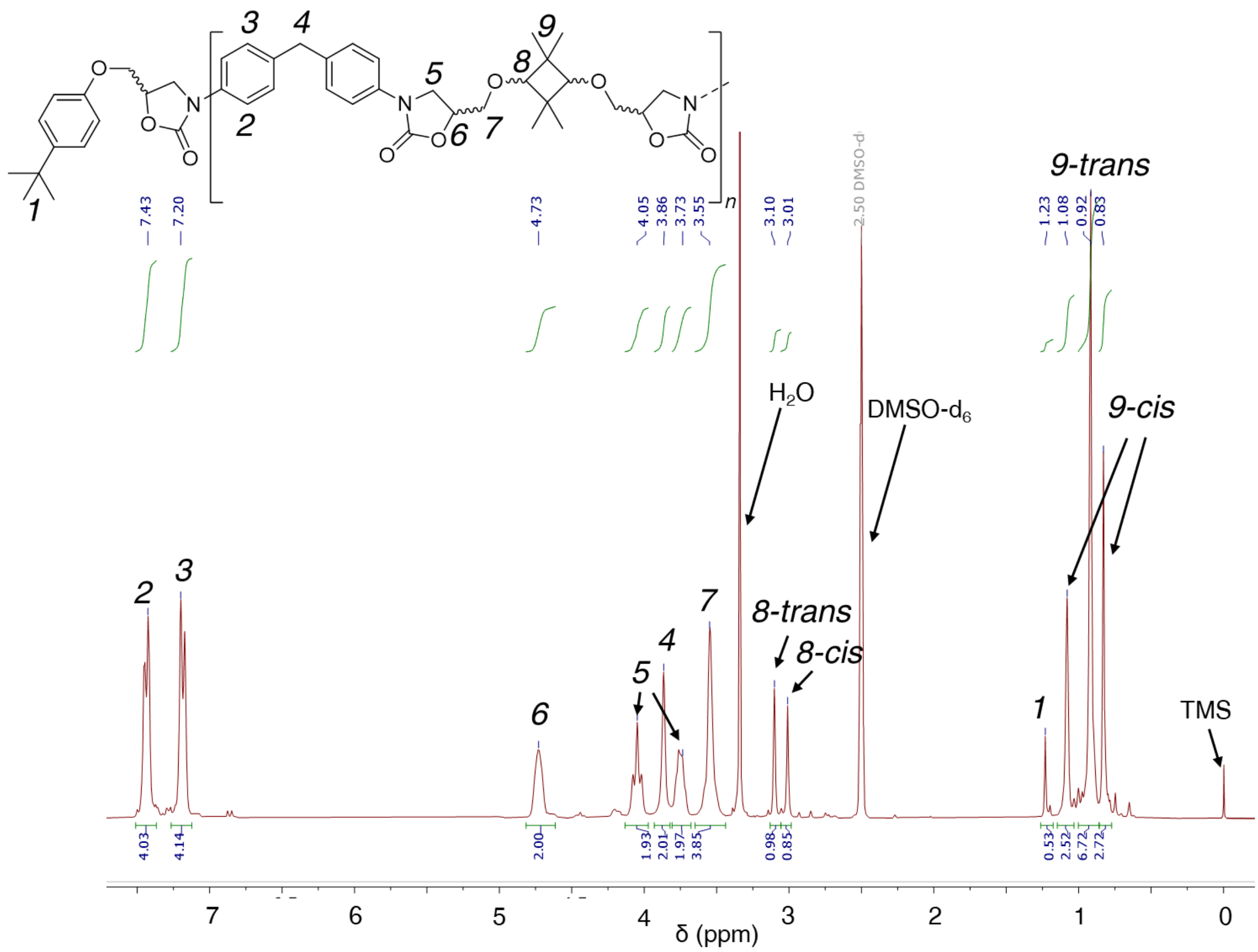


***Figure S35.*** *$^1$H-NMR spectrum of the CBDGE-based polyoxazolidinone in DMSO-d$_6$ (400 MHz).*

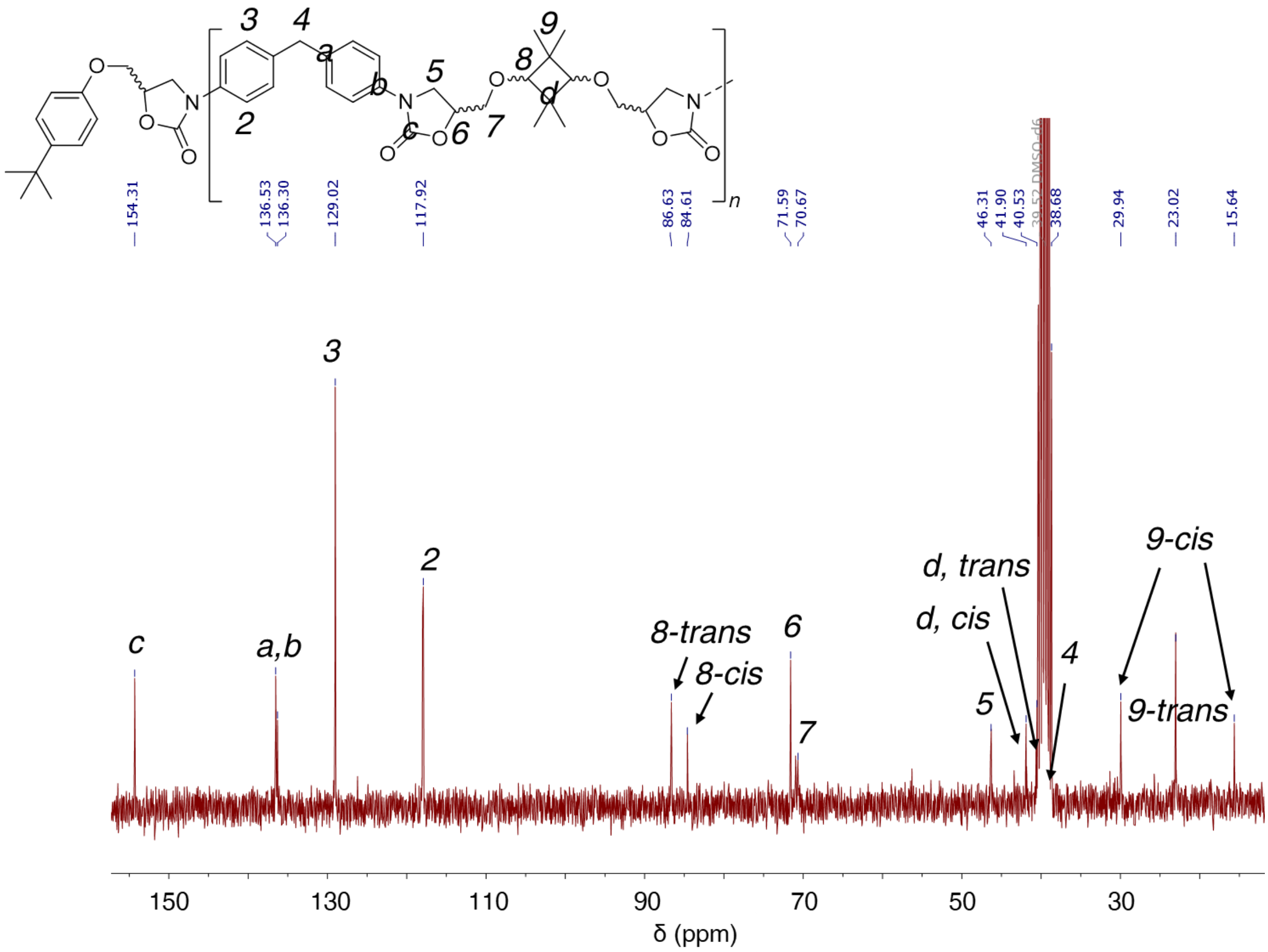


***Figure S36.*** *$^{13}$C-NMR spectrum of the CBDGE-based polyoxazolidinone in DMSO-$d_6$ (400 MHz).*

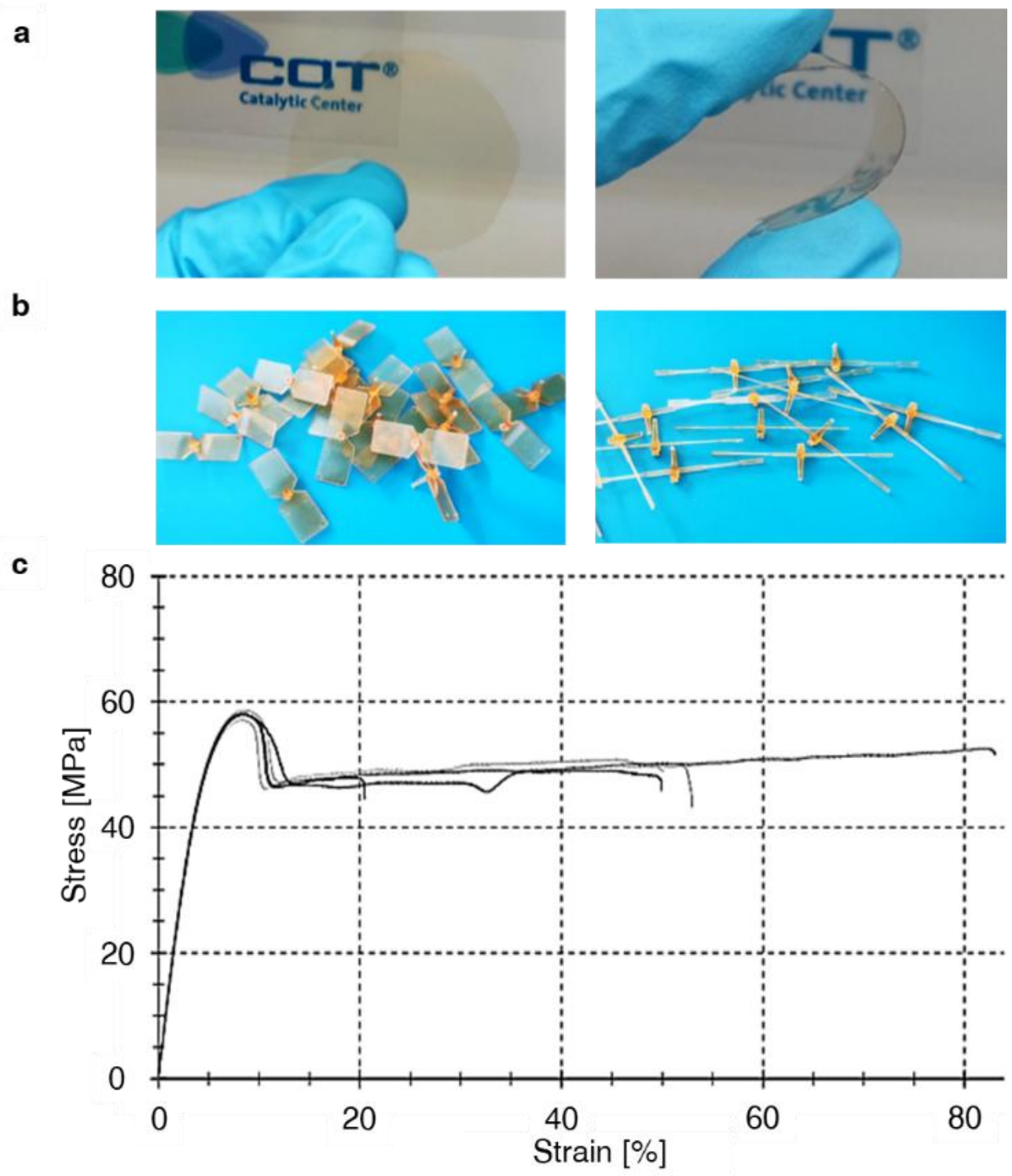


***Figure S37.*** *(a) Transparent, somewhat flexible plates from polyoxazolidinone with CBDGE as bisepoxide were obtained in a thermal press at 170 °C and 10 kN for 30 s. (b) Test specimen obtained by micro-injection moulding at KUZ Leipzig. (c) Stress-strain curve of polyoxazolidinone prepared with 4,4'-MDI and CBDGE.*

**TCDDGE-derived polyoxazolidinone**

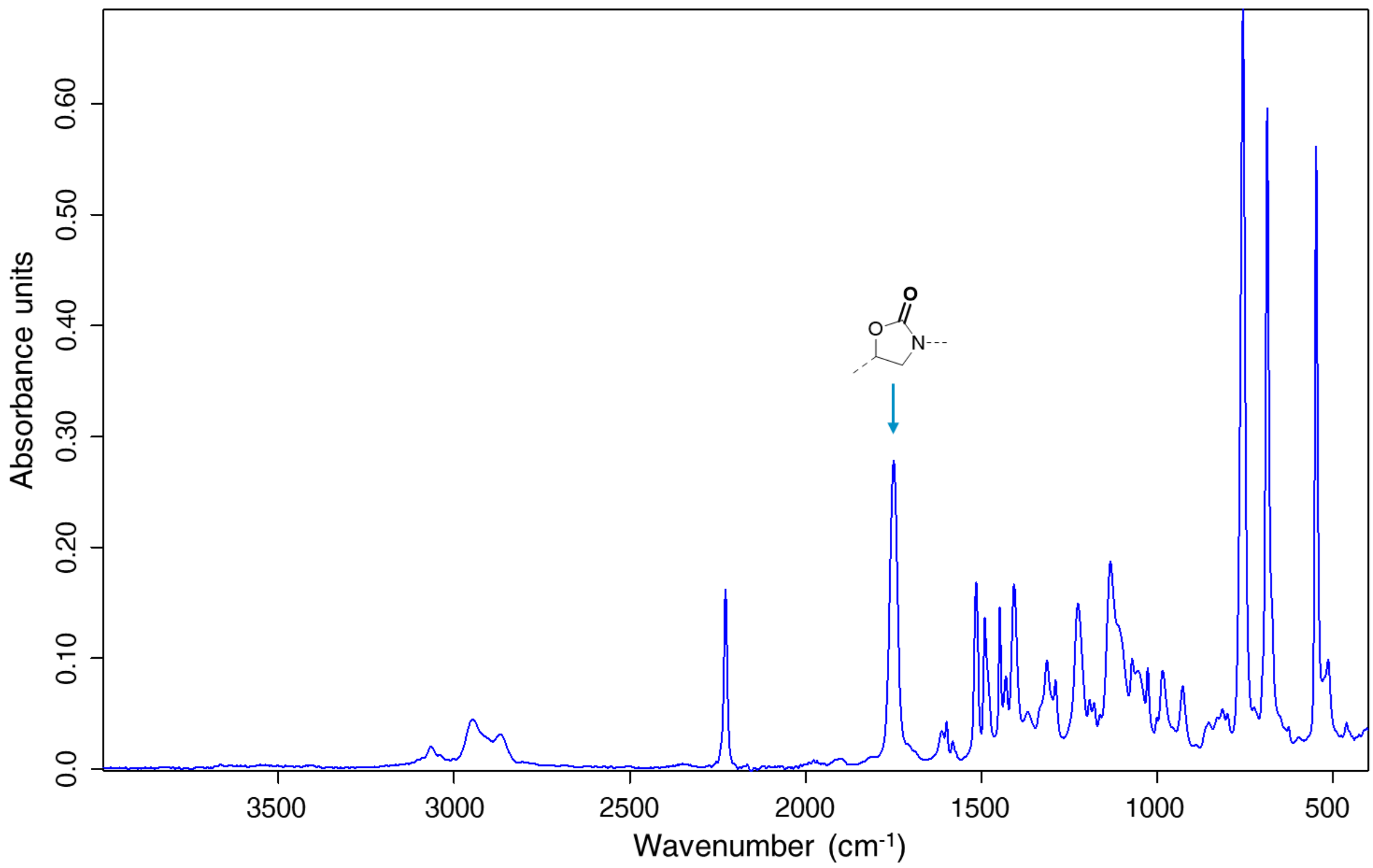


***Figure S38.*** *FTIR-spectrum of TCDDGE-based polyoxazolidinone at the end of the reaction.*

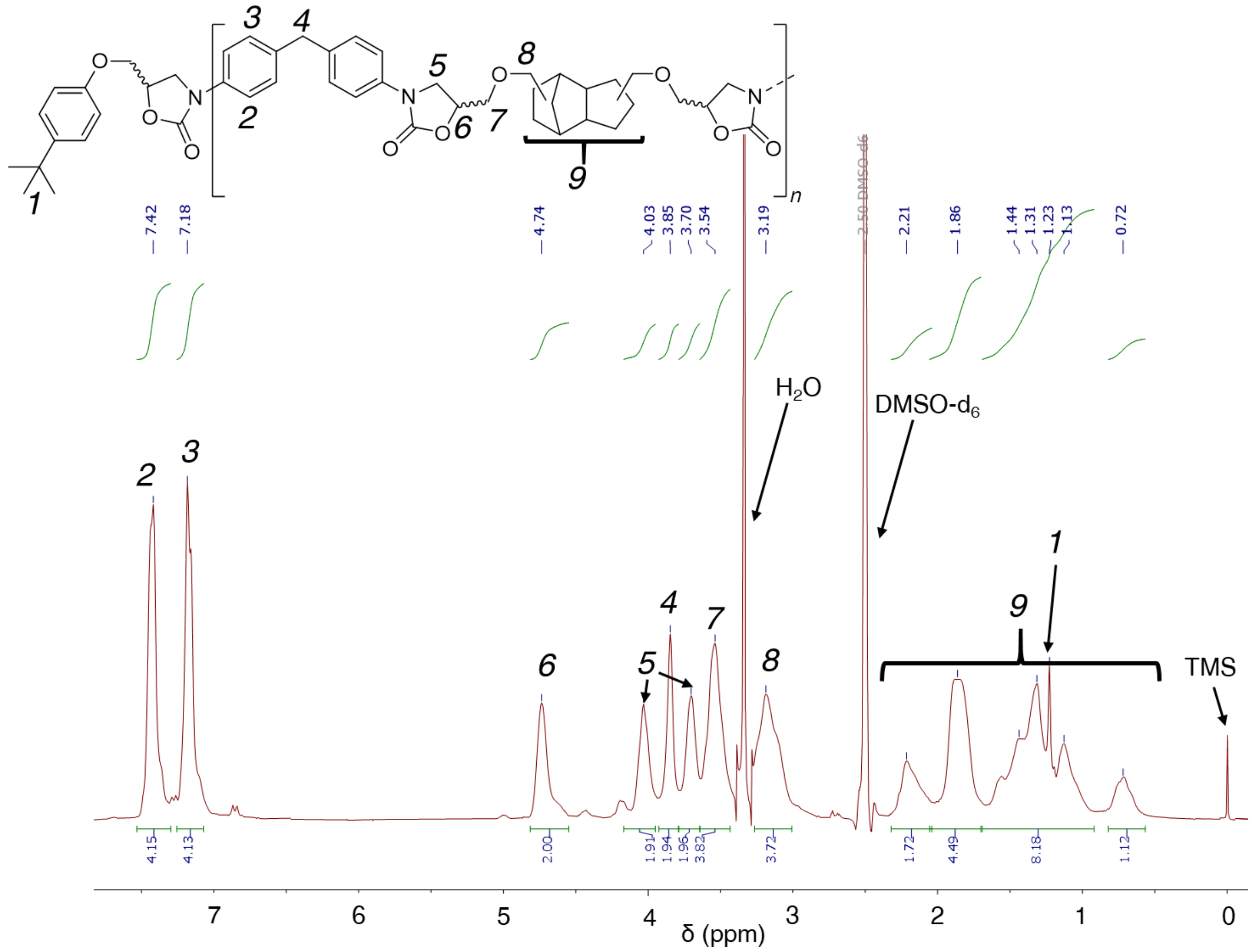


***Figure S39.*** *$^1$H-NMR spectrum of the TCDDGE-based polyoxazolidinone in DMSO-d$_6$ (400 MHz).*

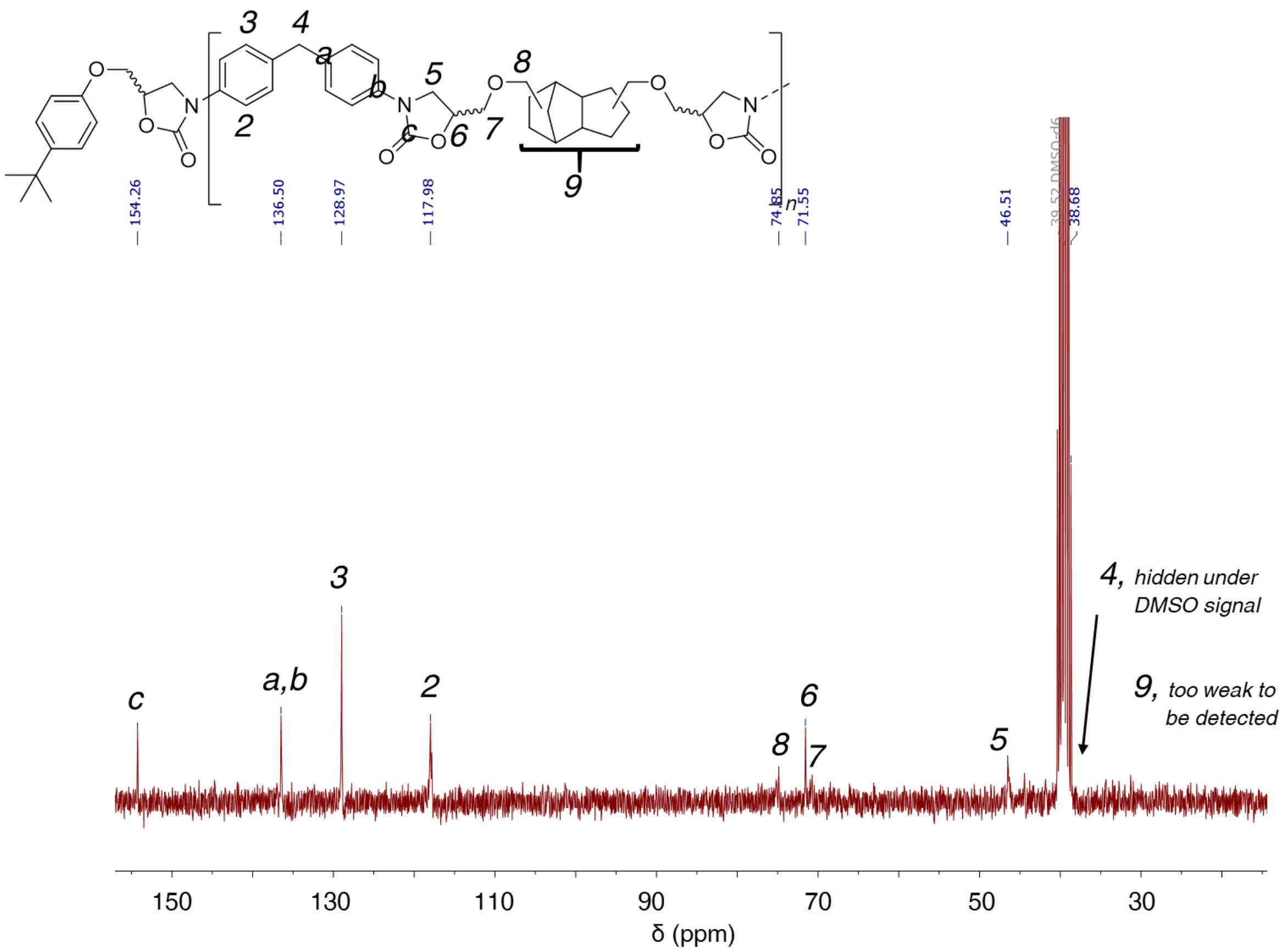


***Figure S40.*** *$^{13}$C-NMR spectrum of the TCDDGE-based polyoxazolidinone in DMSO-$d_6$ (400 MHz).*

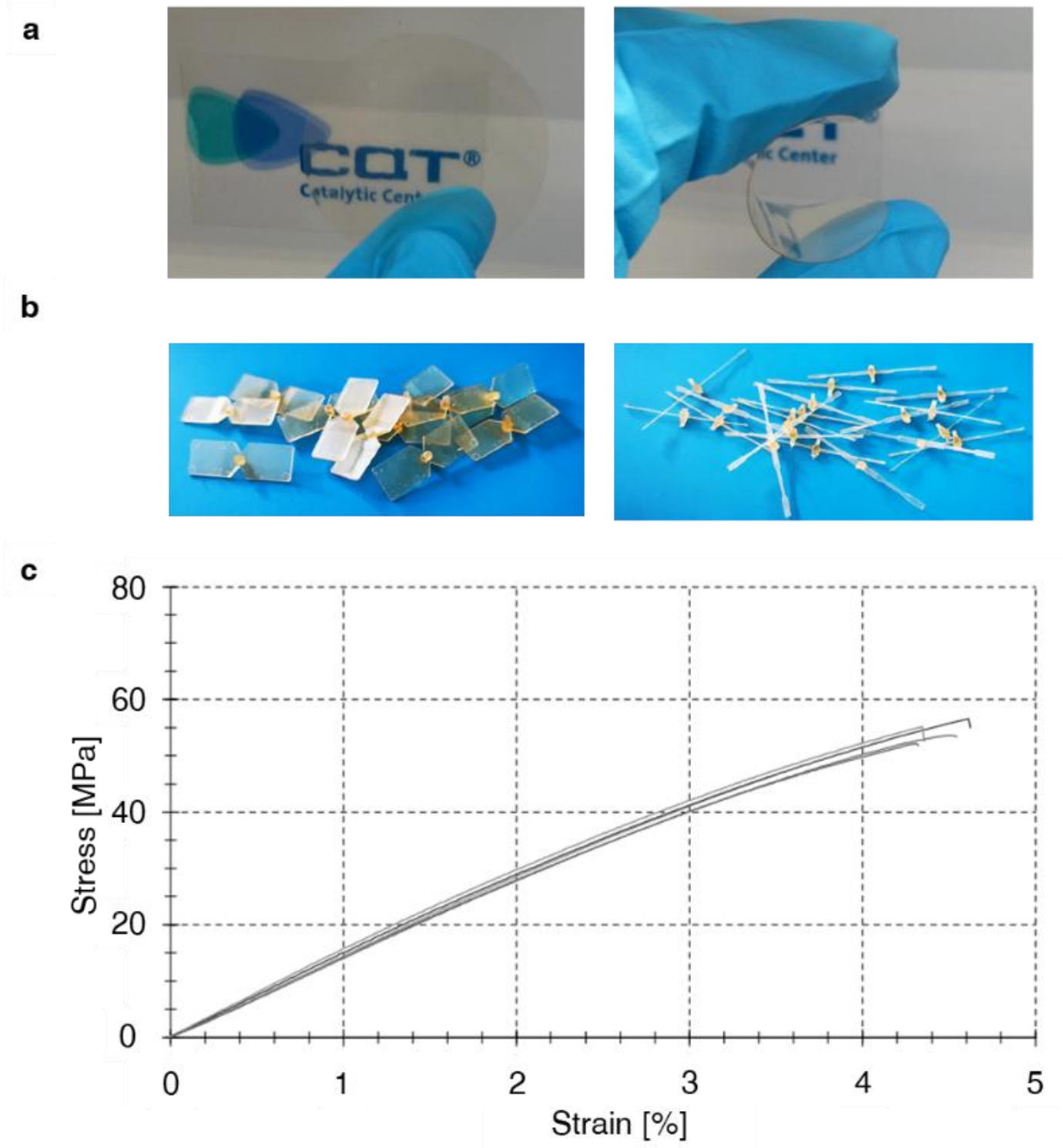


***Figure S41.*** *(a) Flexible, transparent plates from polyoxazolidinone with TCDDGE as bisepoxide were obtained in a thermal press at 150 °C. (b) Test specimen obtained by micro-injection moulding at KUZ Leipzig. (c) Stress-strain curve of polyoxazolidinone prepared with 4,4'-MDI and TCDDGE.*

## ISDGE-derived polyoxazolidinone

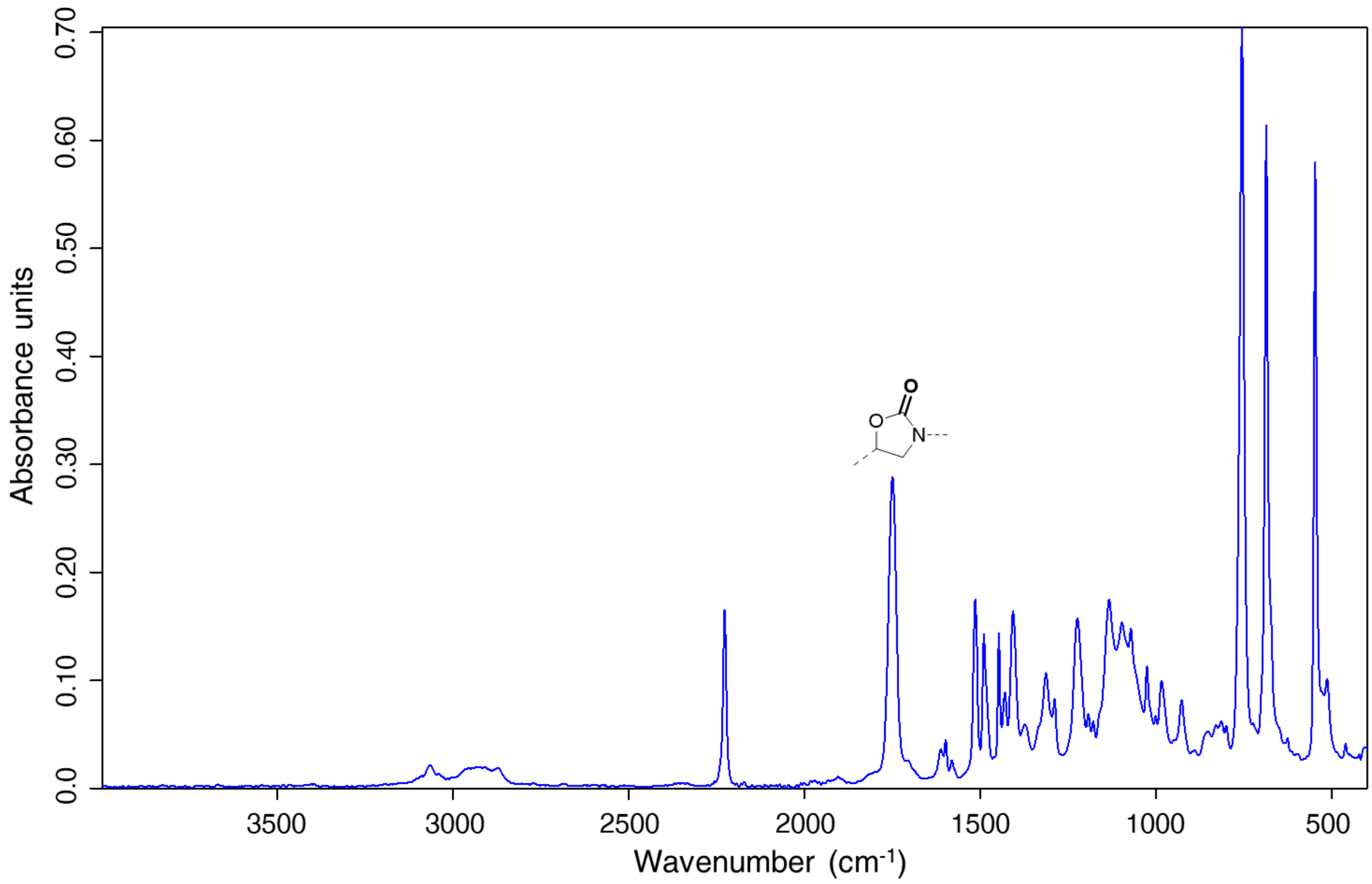


***Figure S42.*** *FTIR-spectrum of ISDGE-based polyoxazolidinone during the reaction.*

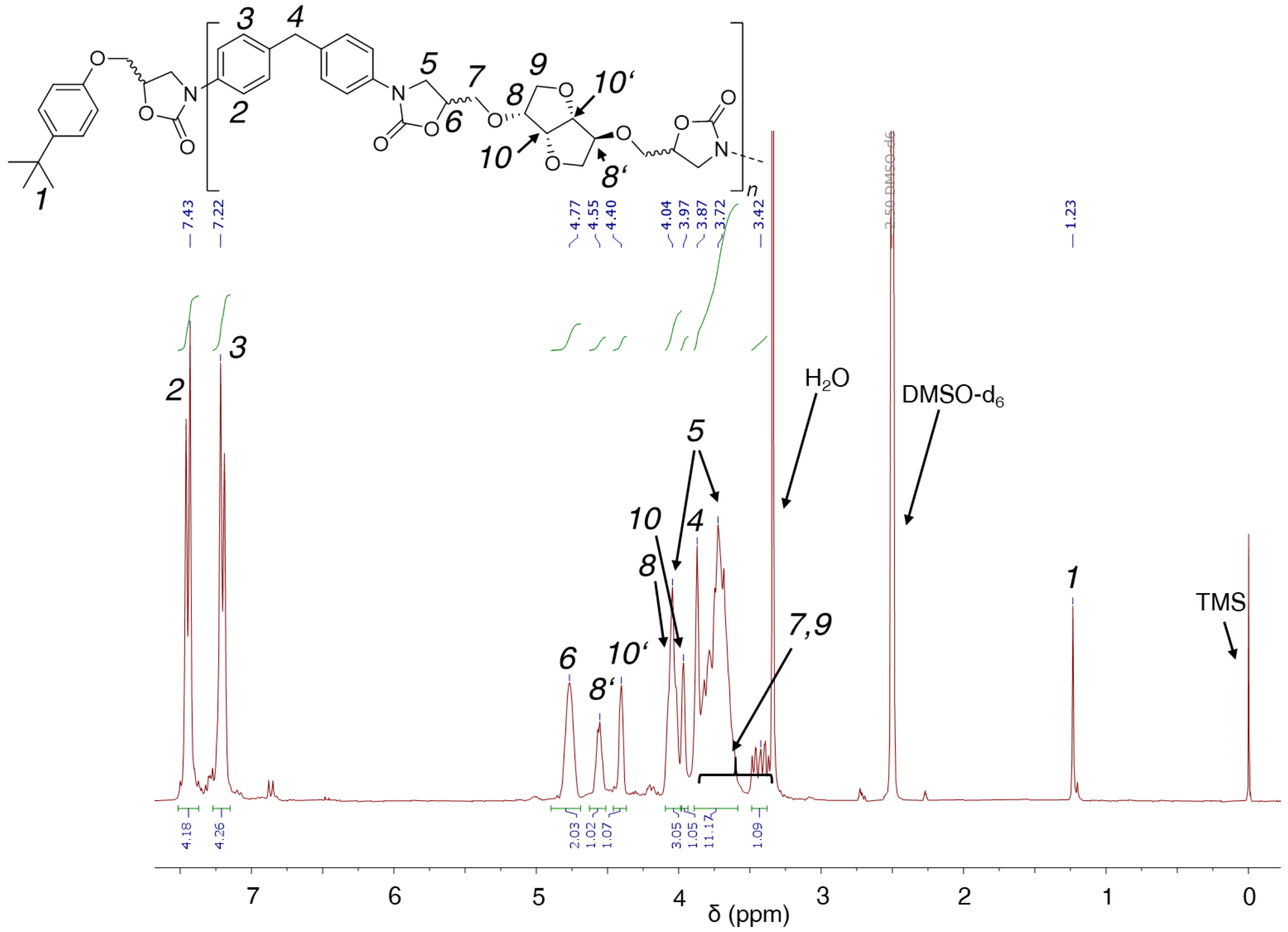


***Figure S43.*** *$^1$H-NMR spectrum of the ISDGE-based polyoxazolidinone in DMSO-d$_6$ (400 MHz).*

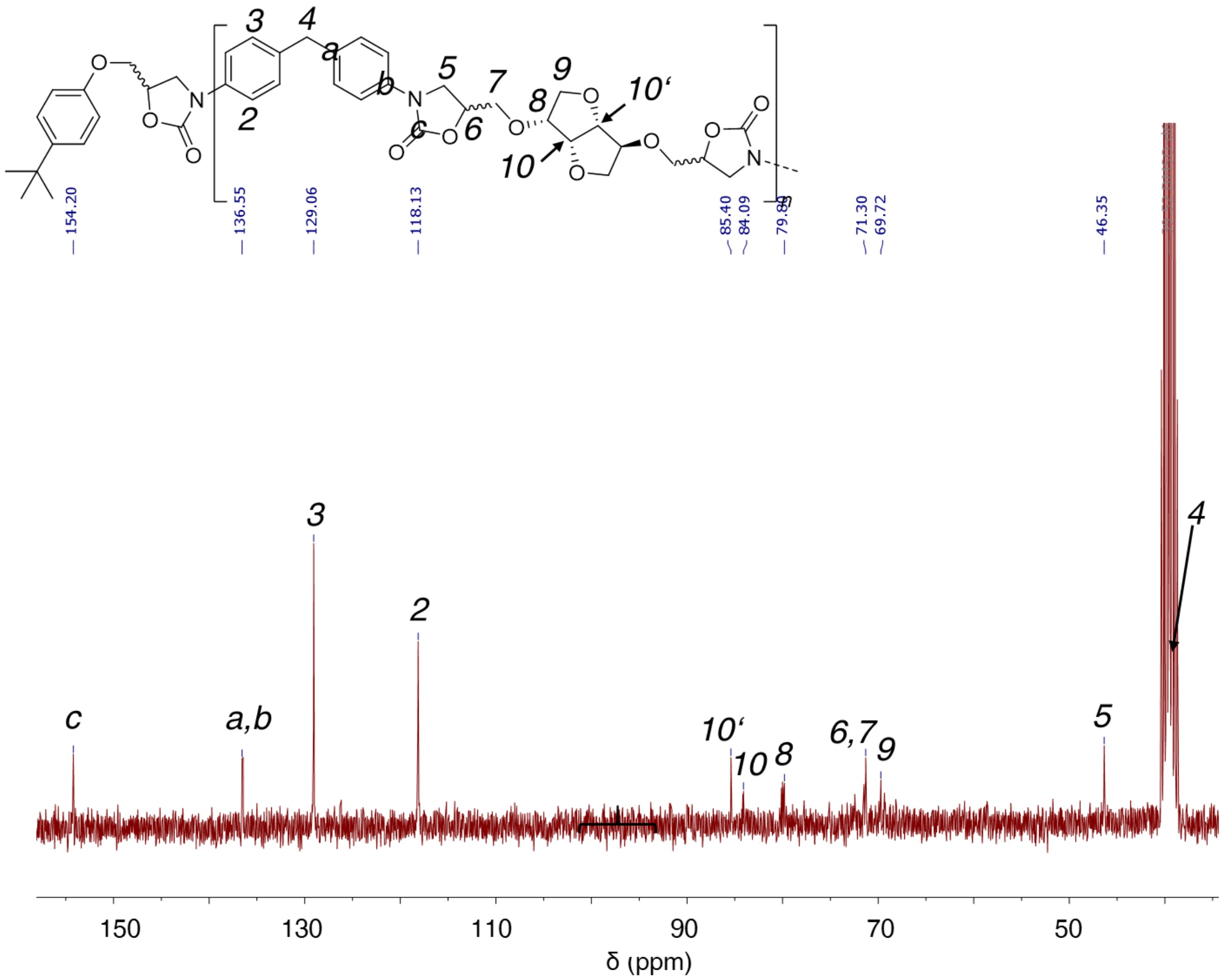


***Figure S44.*** *$^{13}$C-NMR spectrum of the ISDGE-based polyoxazolidinone in DMSO-d$_6$ (400 MHz).*

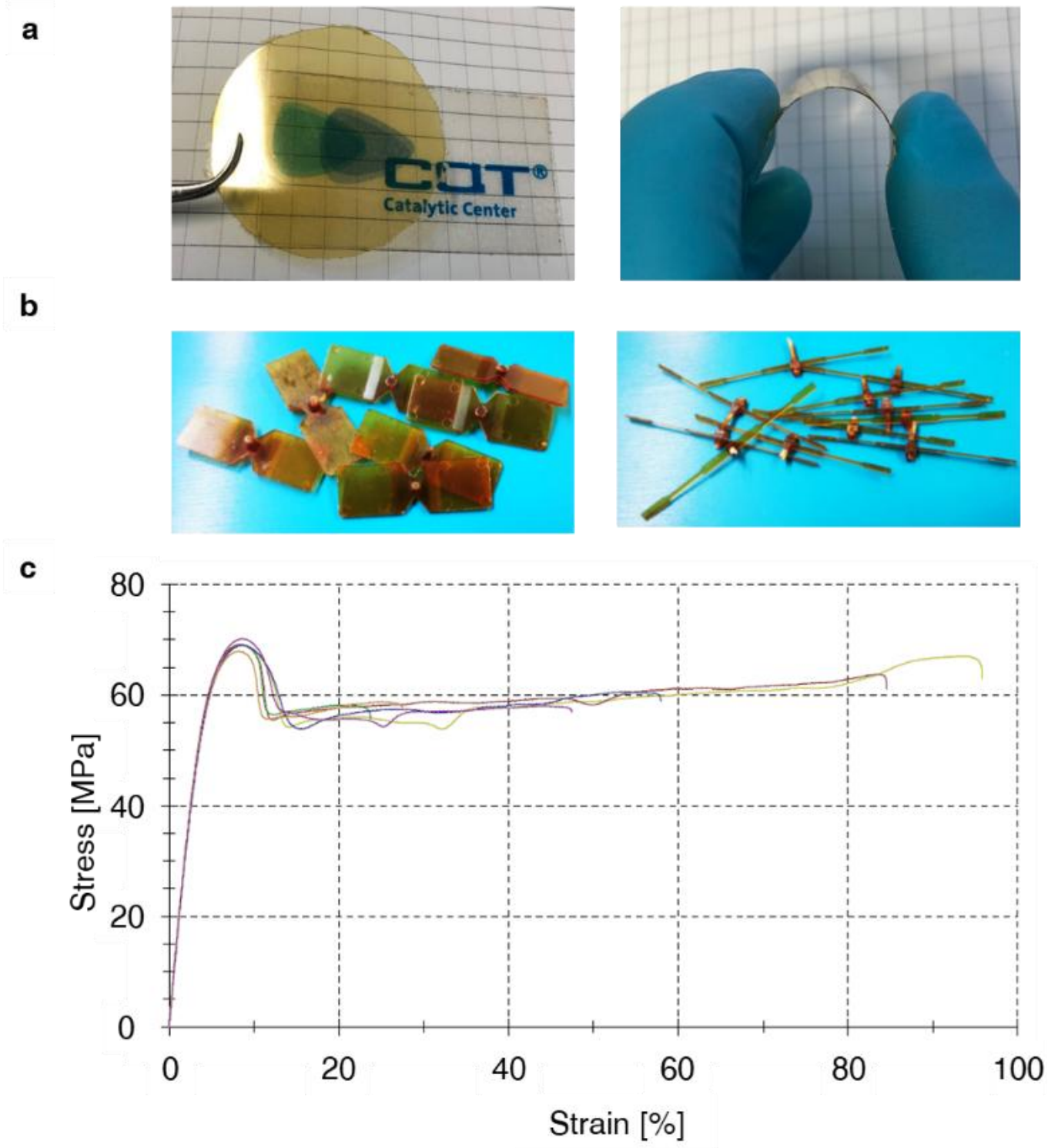


***Figure S45.*** *(a) Flexible, somewhat brownish, transparent plates from polyoxazolidinone with ISDGE as bisepoxide were obtained in a thermal press at 180 °C. (b) Test specimen obtained by micro-injection molding at KUZ Leipzig. (c) Stress-strain curve of polyoxazolidinone prepared with 4,4'-MDI and ISDGE.*

## TPADGE-derived polyoxazolidinone

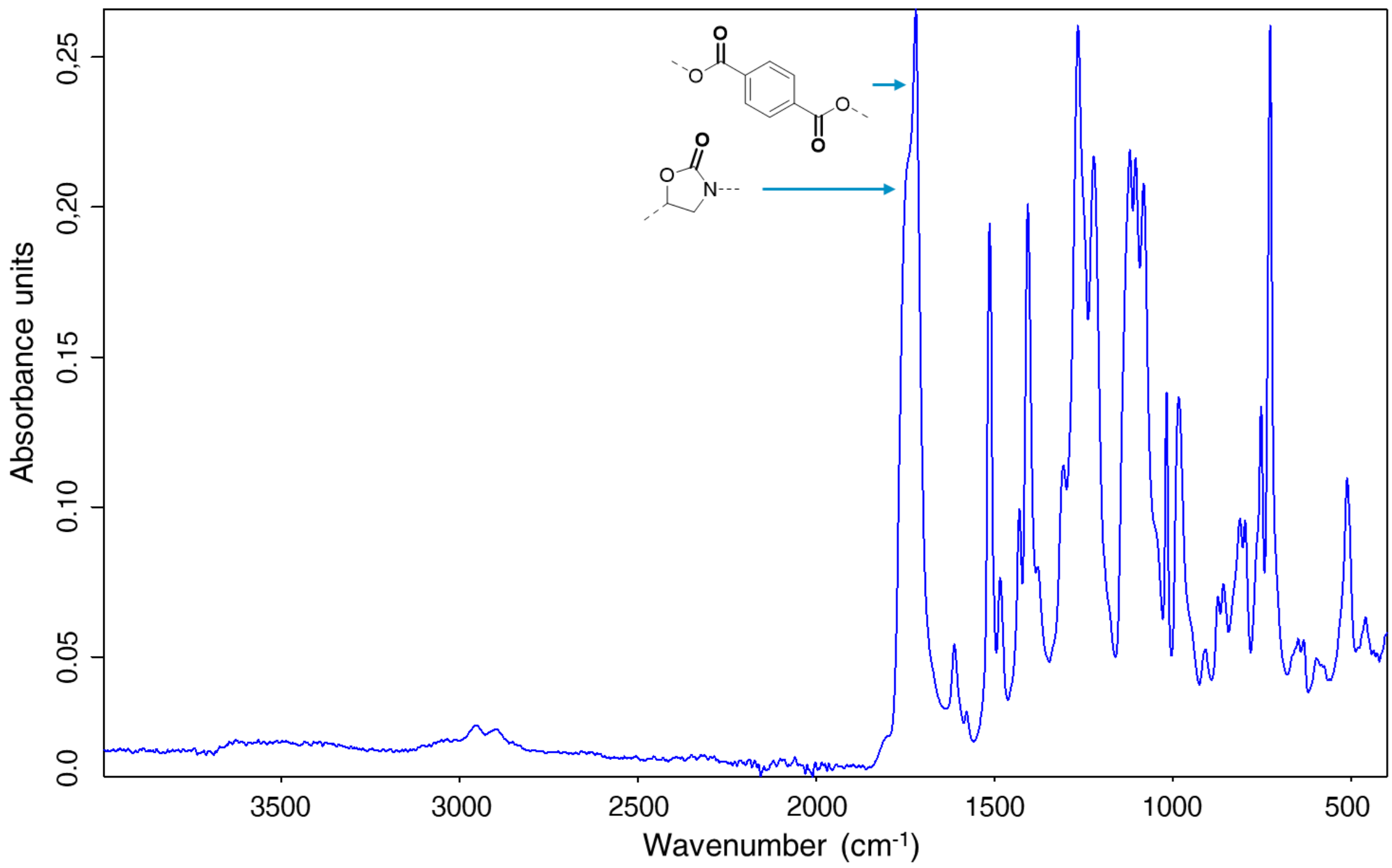


***Figure S46.*** *FTIR-spectrum of TPADGE-based polyoxazolidinone (solid polymer).*

NMR spectra were not recorded due to the insolubility of the material.

## BDMDGE-derived polyoxazolidinone

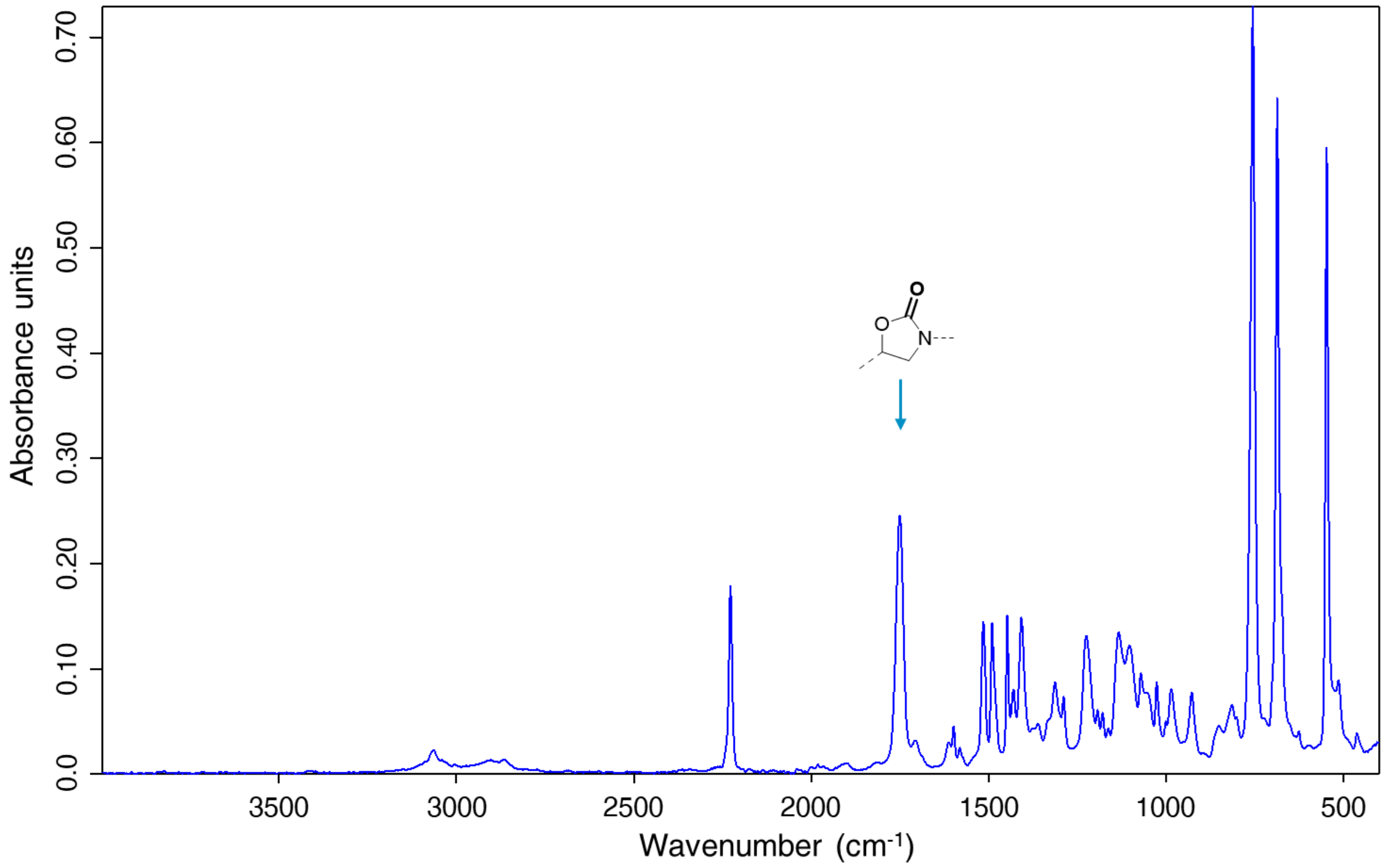


***Figure S47.*** *FTIR-spectrum of BDMDGE-based polyoxazolidinone (120 min reaction time).*

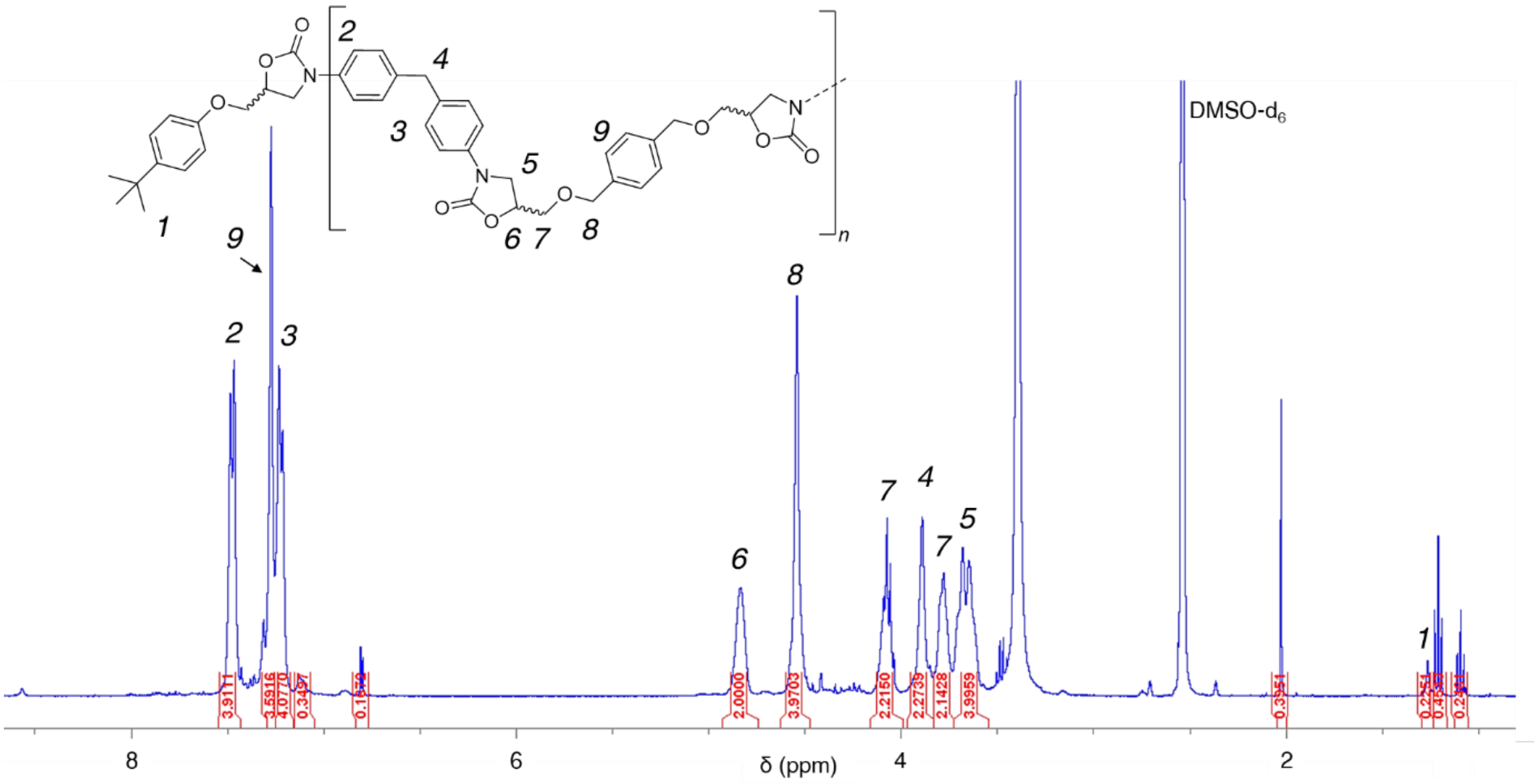


***Figure S48.*** *$^{1}$H-NMR spectrum of the BDMDGE-based polyoxazolidinone in DMSO-d*$_6$ *(400 MHz).*

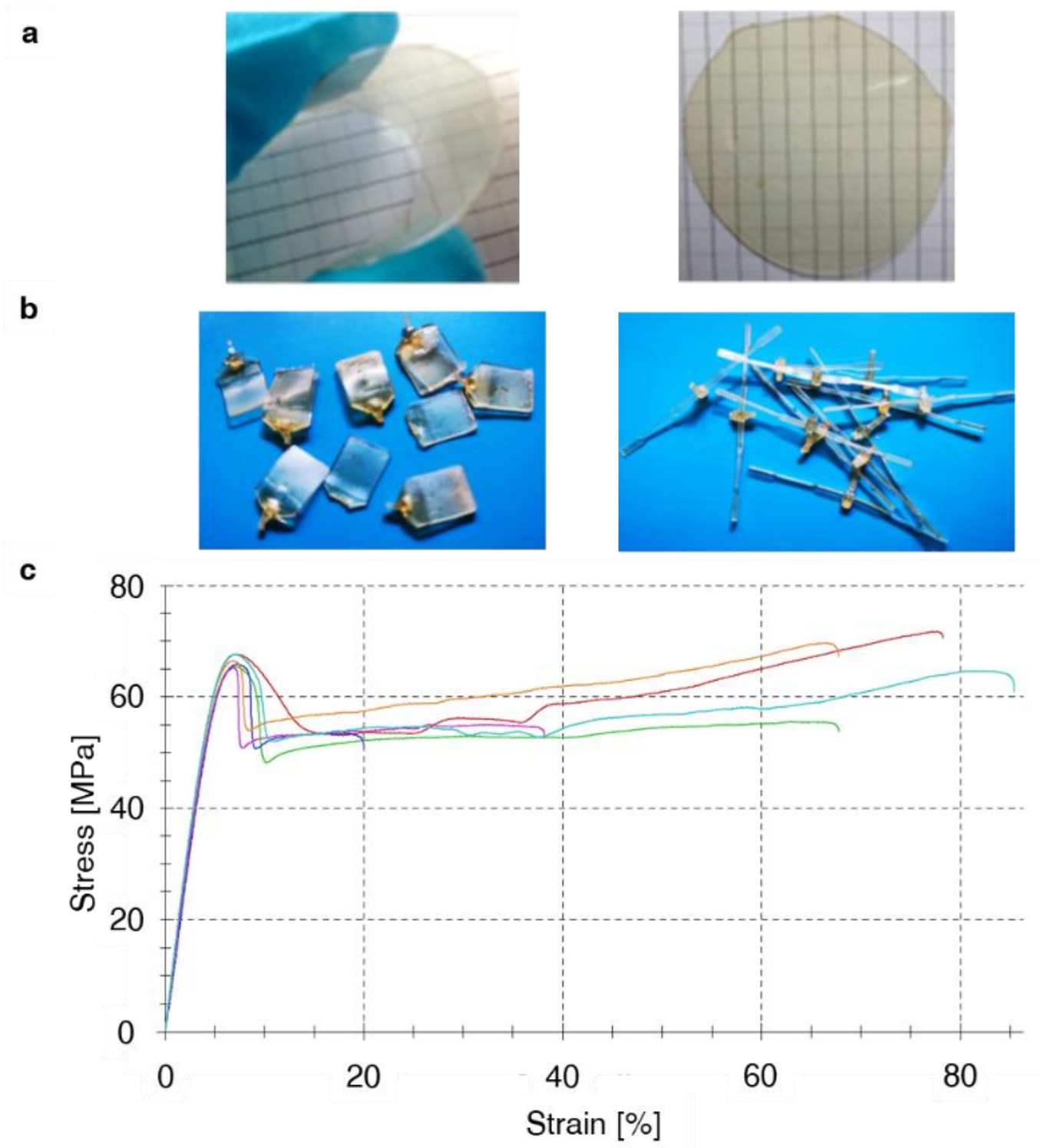


***Figure S49.*** *(a) Flexible, slightly brownish, transparent plates from polyoxazolidinone with BDMDGE as bisepoxide were obtained in a thermal press at 160 °C and 10 kN for 30 s. (b) Test specimen obtained by micro-injection moulding at KUZ Leipzig; deformation (left) due to the elasticity and stickiness of the polymer. (c) Stress-strain curve of polyoxazolidinone prepared with 4,4'-MDI and BDMDGE.*

**DMHBE-derived polyoxazolidinone**

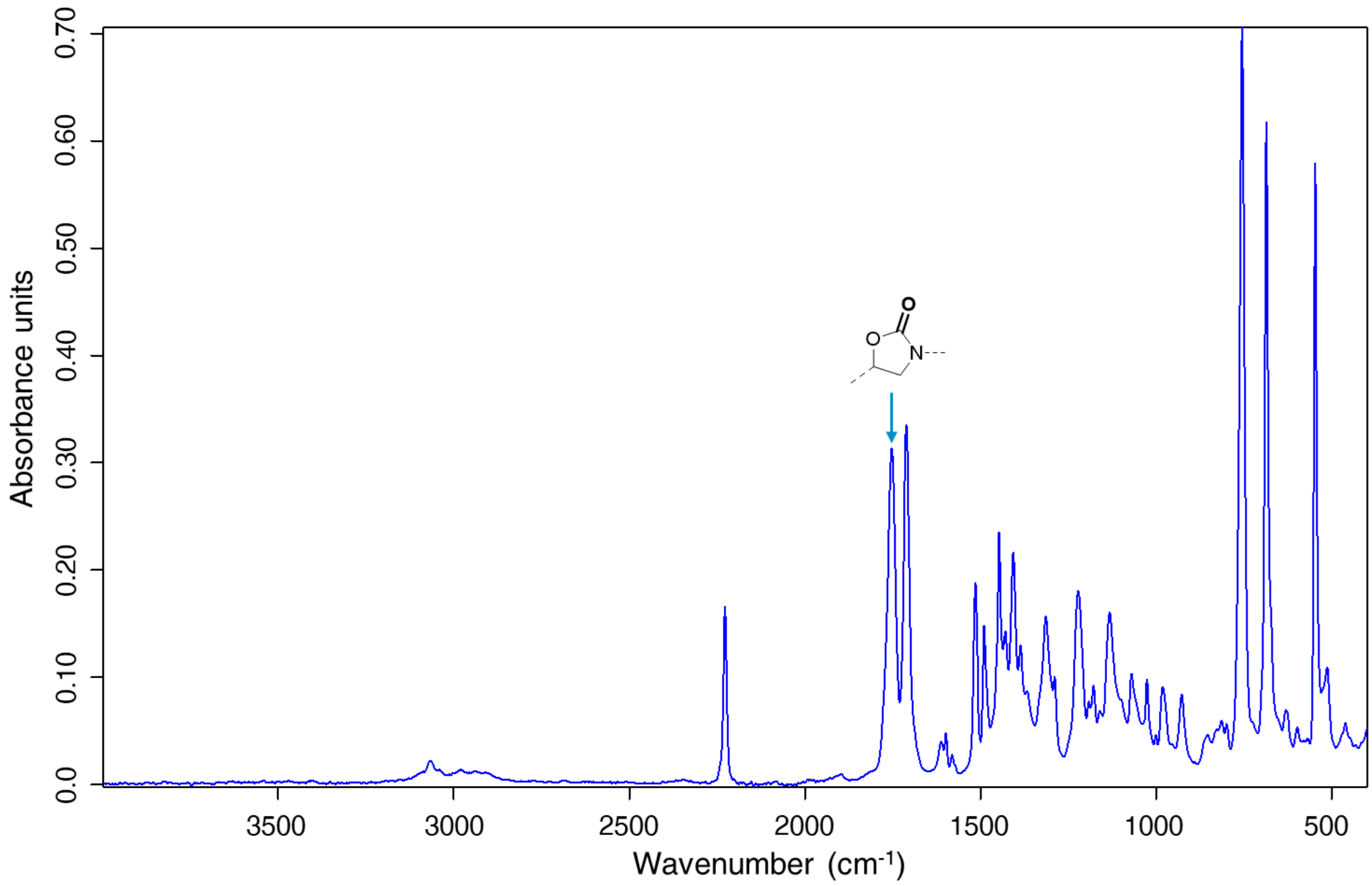


***Figure S50.*** *FTIR-spectrum of DMHBE-based polyoxazolidinone at the end of the reaction. The signal at 1712 cm$^{-1}$ corresponds with the carbonyl of the hydantoin moiety.*

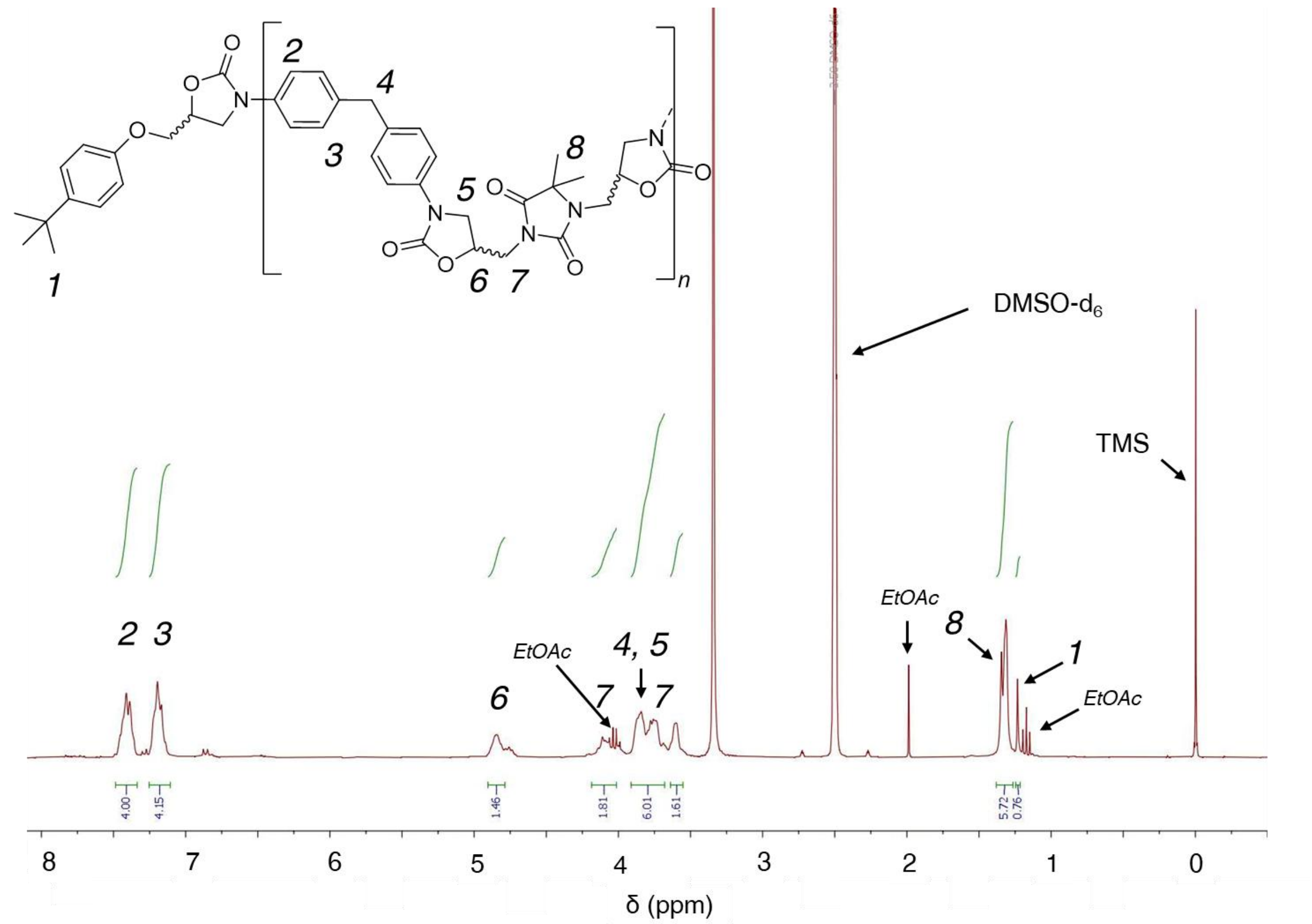


***Figure S51.** $^{1}$H-NMR spectrum of the DMHBE-based polyoxazolidinone in DMSO-d*$_6$ *(300 MHz).*

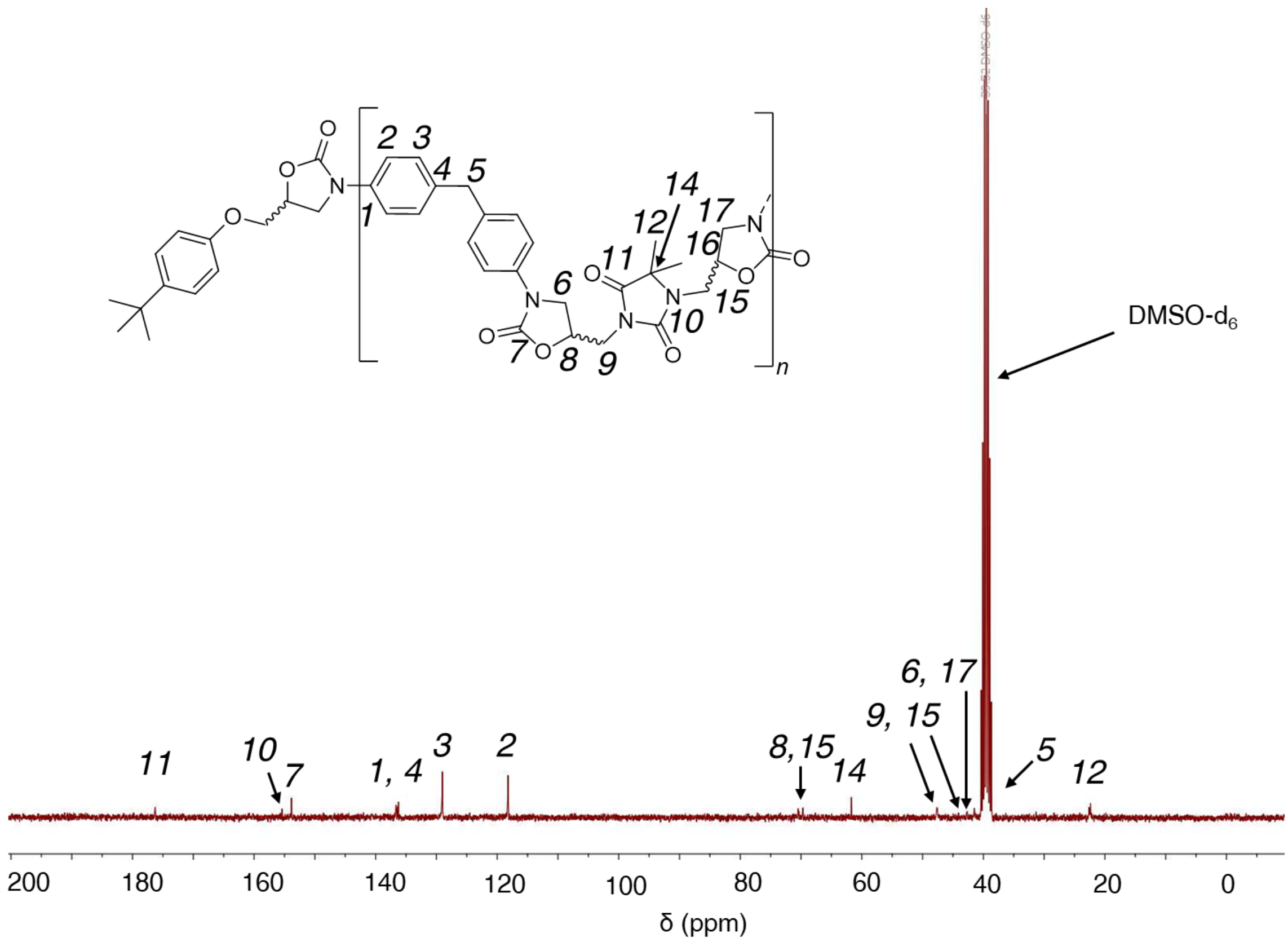


***Figure S52.*** *$^{13}$C-NMR spectrum of the DMHBE-based polyoxazolidinone in DMSO-d$_6$ (300 MHz).*

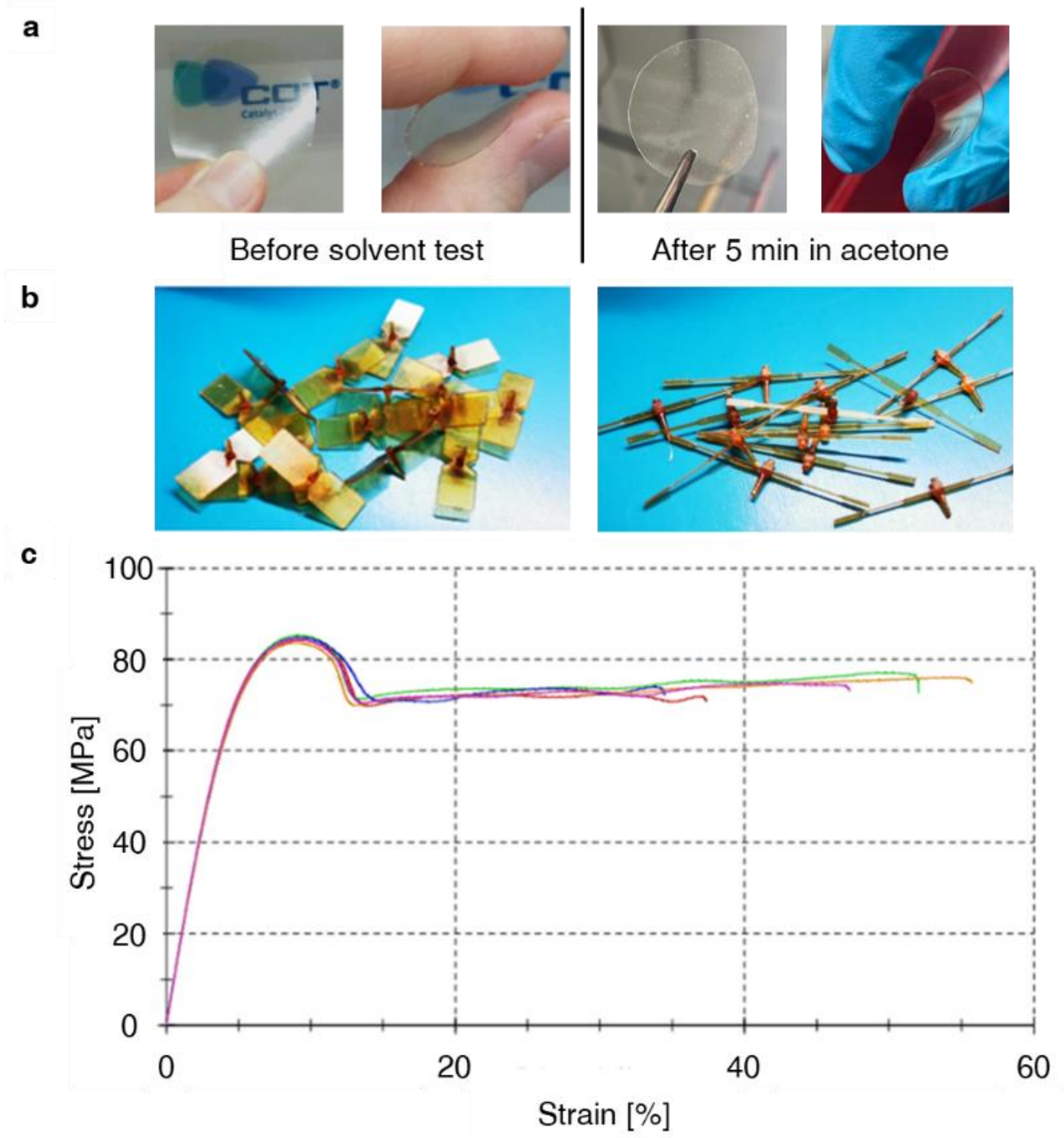


***Figure S53.*** *(a) Flexible, transparent plates from polyoxazolidinone with DMHBE as bisepoxide were obtained in a thermal press at 240 °C and 10 kN for 30 s. In a preliminary solvent resistance test, the plate was dipped in acetone for 5 min and optically examined afterwards. (b) Test specimen obtained by micro-injection moulding at KUZ Leipzig. (c) Stress-strain curve of polyoxazolidinone prepared with 4,4'-MDI and DMHBE.*

## DGI-derived polyoxazolidinone

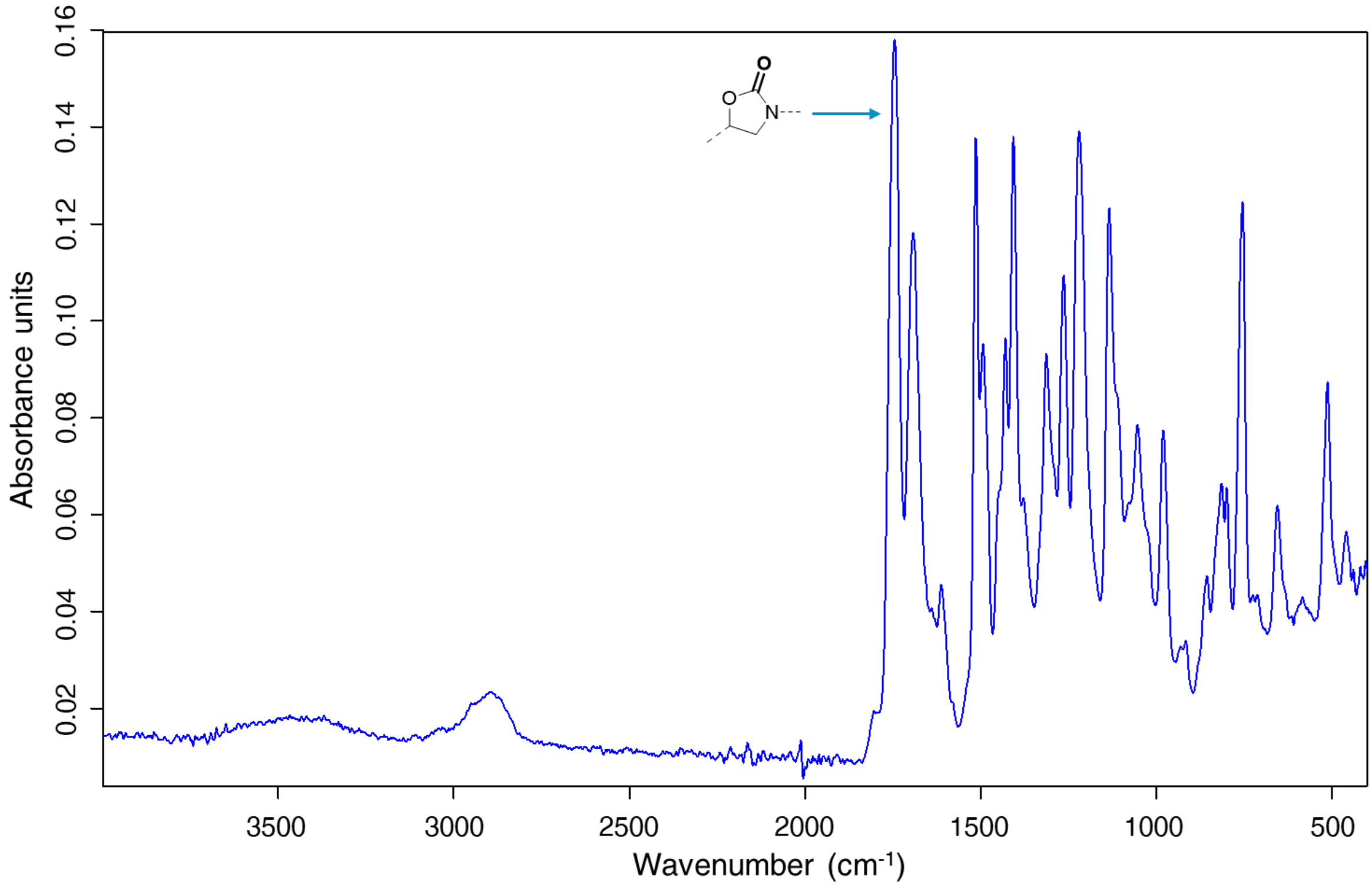


***Figure S54.*** *FTIR-spectrum of DGI-based polyoxazolidinone (solid polymer). The signal at ~ 1710 cm$^{-1}$ corresponds with the carbonyl of the imidazolidinone moiety.*

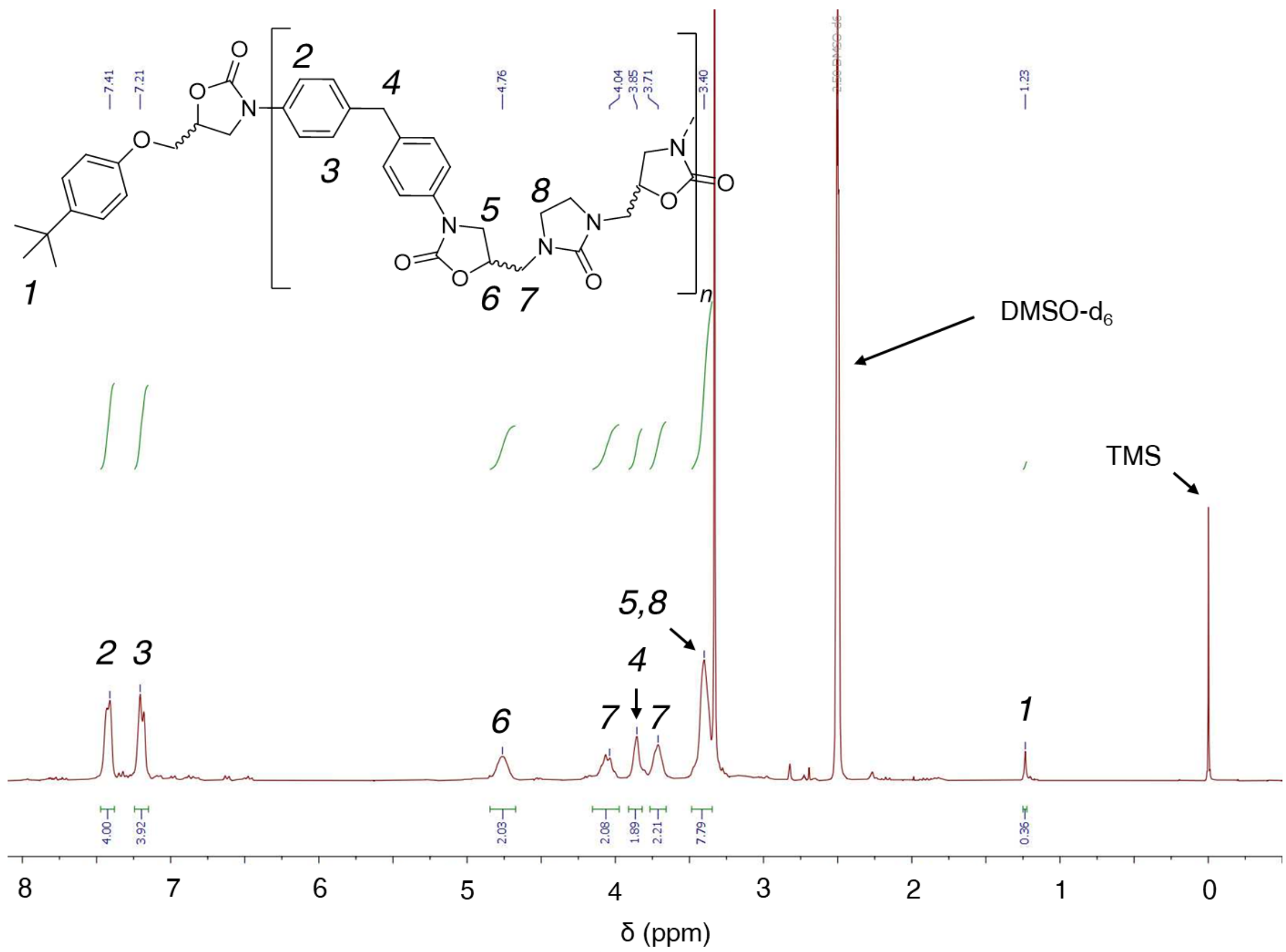


***Figure S55.*** *$^1$H-NMR spectrum of the DGI-based polyoxazolidinone in DMSO-d$_6$ (300 MHz).*

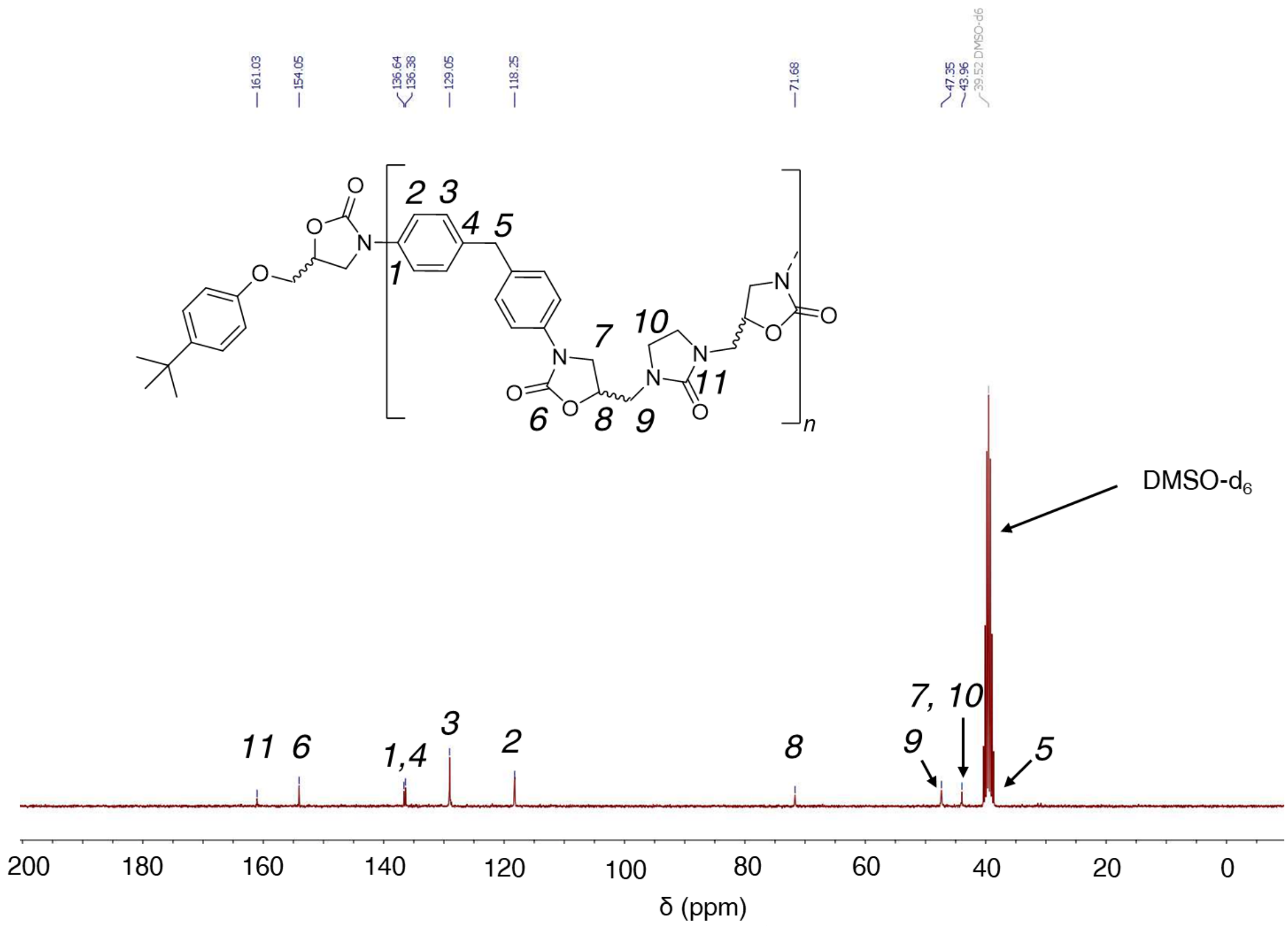


***Figure S56.*** *$^{13}$C-NMR spectrum of the DGI-based polyoxazolidinone in DMSO-d₆ (300 MHz).*

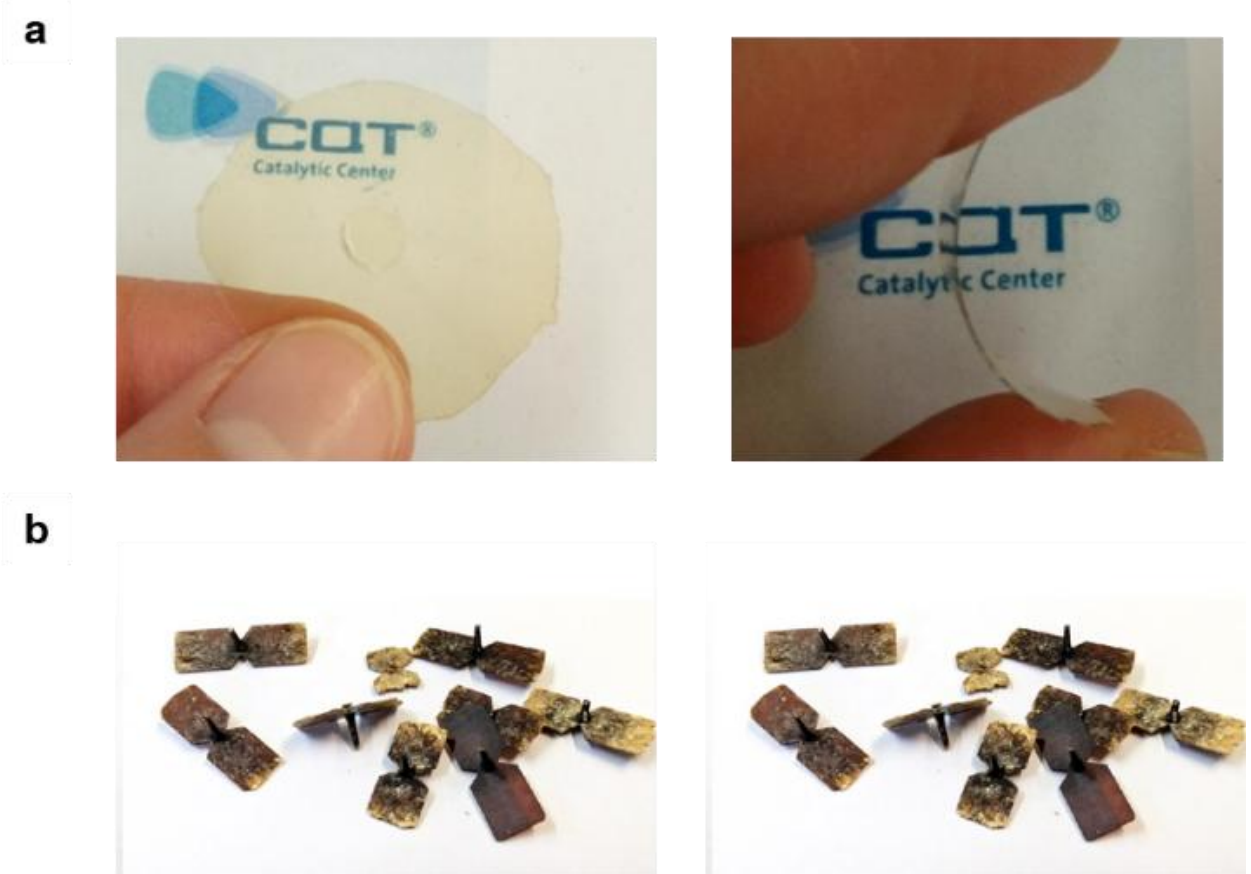


***Figure S57.*** *(a) Somewhat flexible, transparent plates from polyoxazolidinone with DGI as bisepoxide were obtained in a thermal press at 235 and 20 kN for 30-60 s. Inhomogeneous and non-reproducible micro-injection moulded test samples prepared at KUZ Leipzig.*

## DGBI-derived polyoxazolidinone

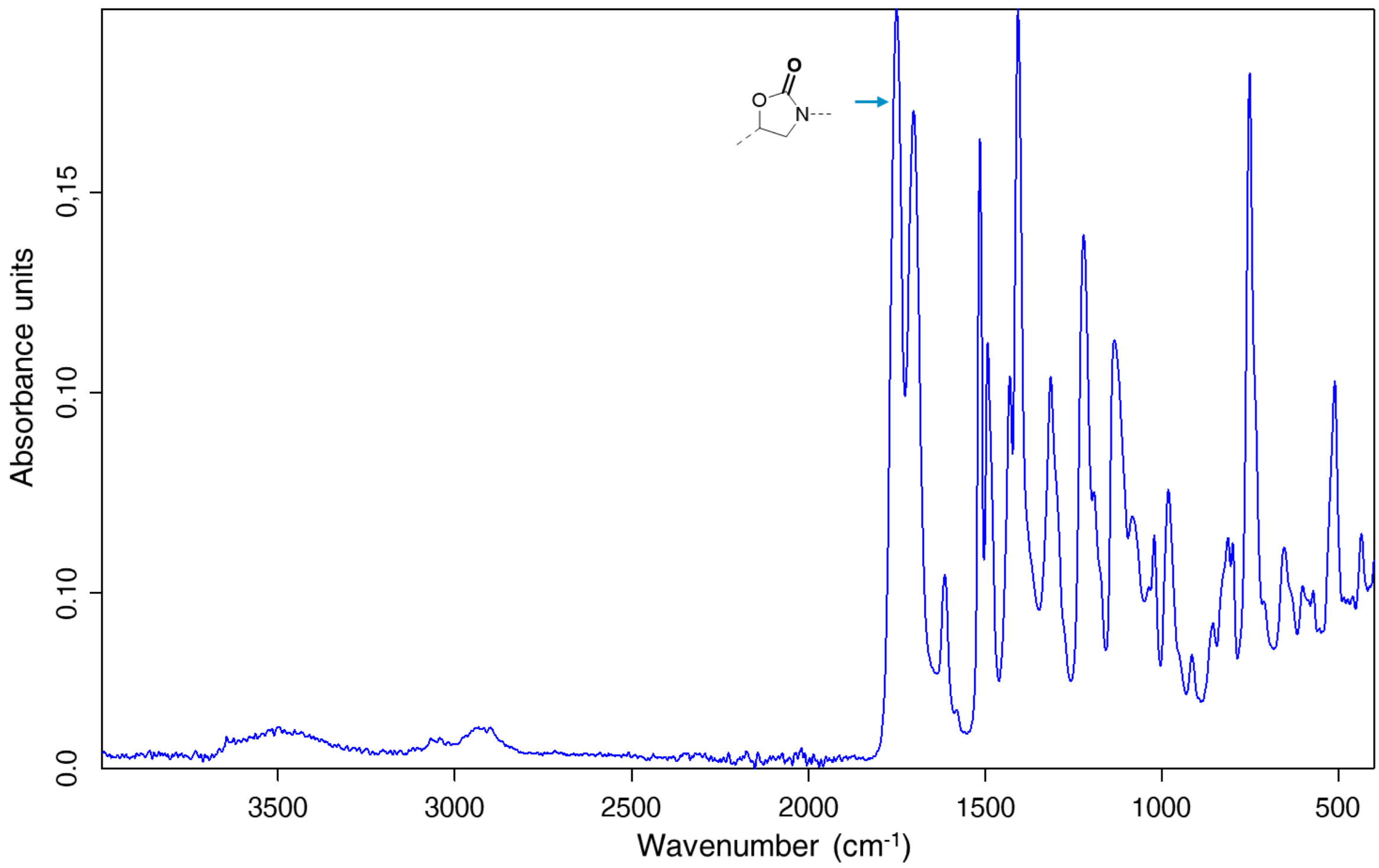


***Figure S58.*** *FTIR-spectrum of DGBI-based polyoxazolidinone (solid polymer). The signal at ~ 1710 cm$^{-1}$ corresponds with the carbonyl of the imidazolidinone moiety.*

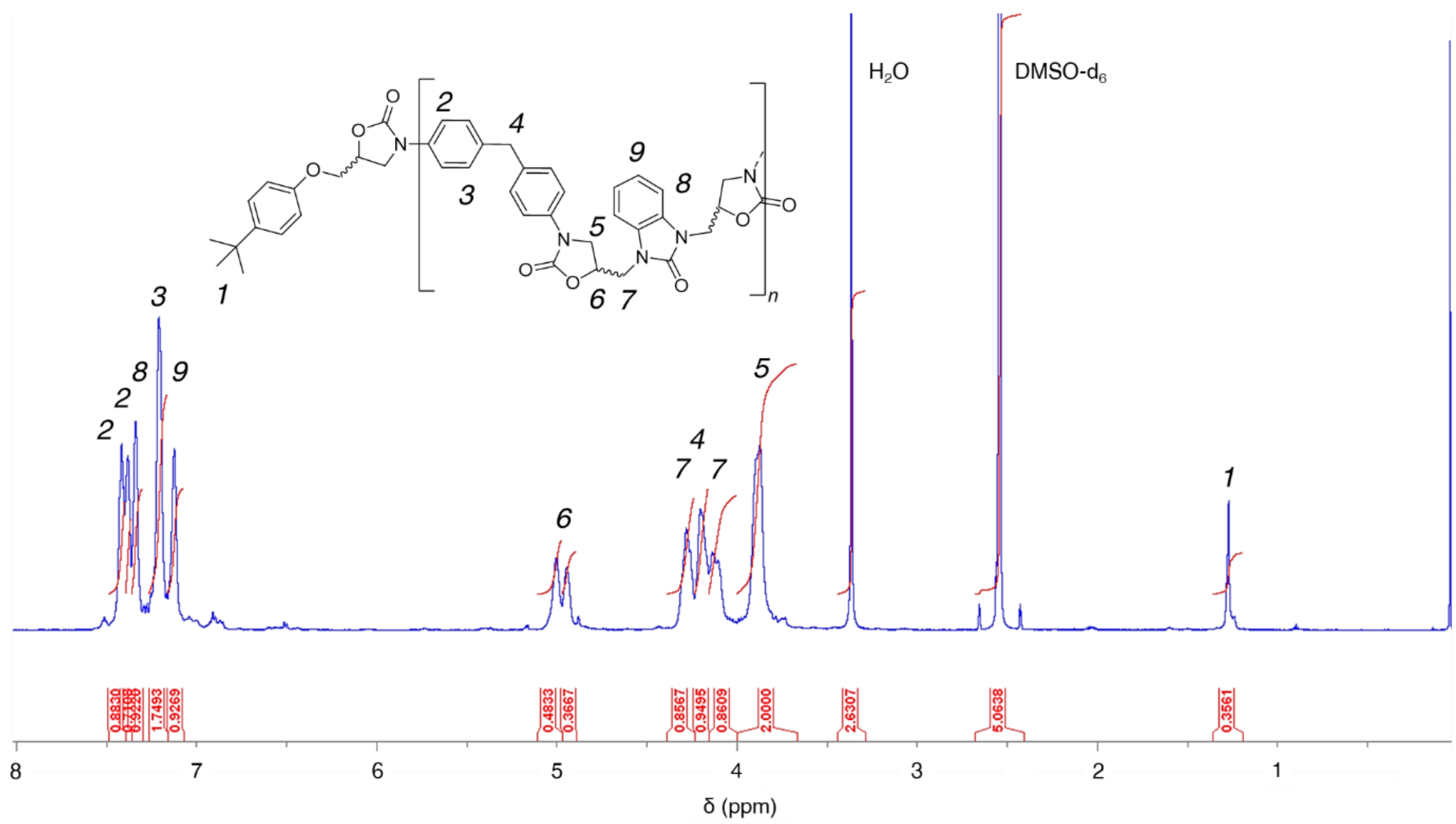


***Figure S59.*** *$^{1}$H-NMR spectrum of the DGBI-based polyoxazolidinone in DMSO-d$_6$ (300 MHz).*[12]

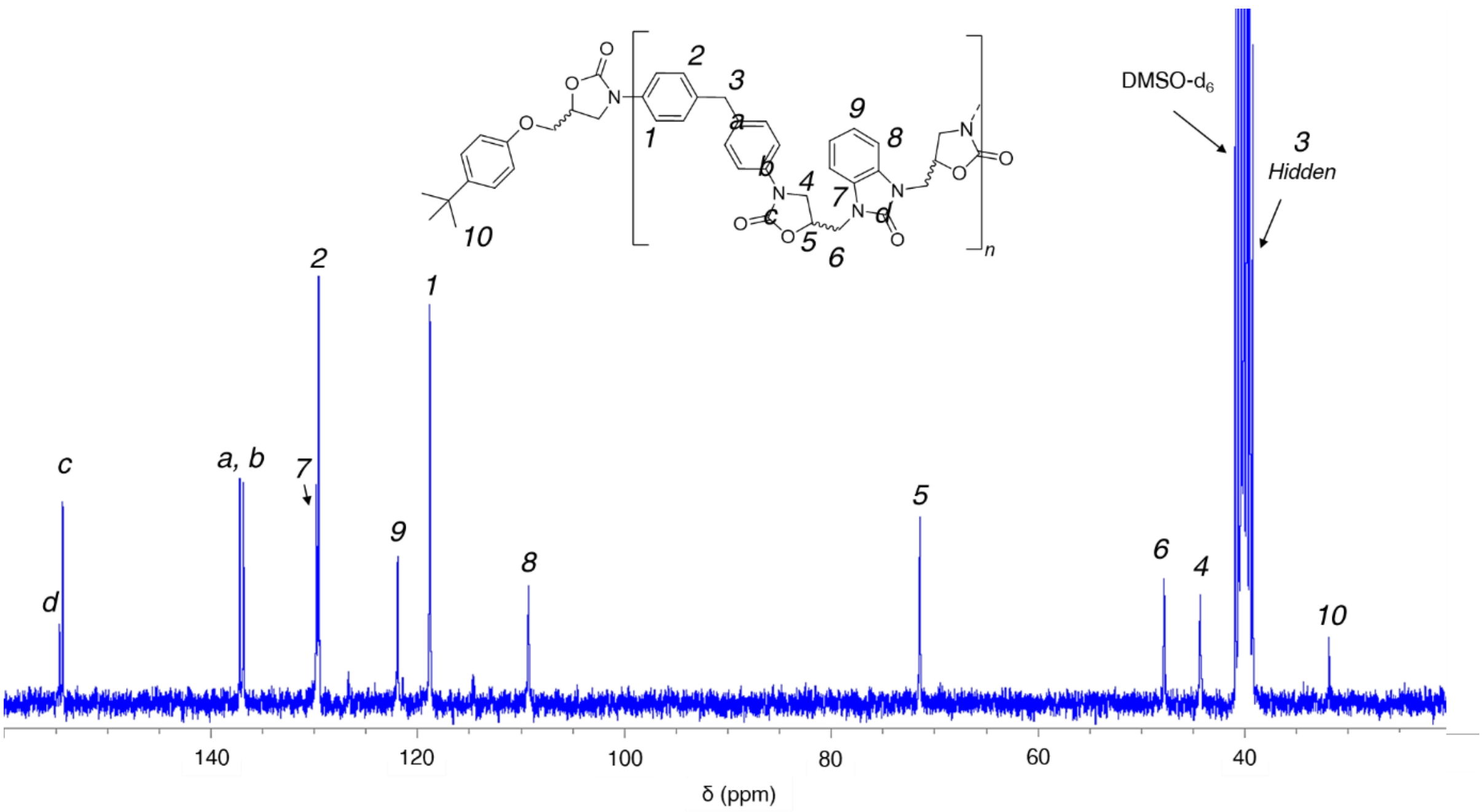


***Figure S60.*** *$^{13}$C-NMR spectrum of the DGBI-based polyoxazolidinone in DMSO-d$_6$ (300 MHz).*

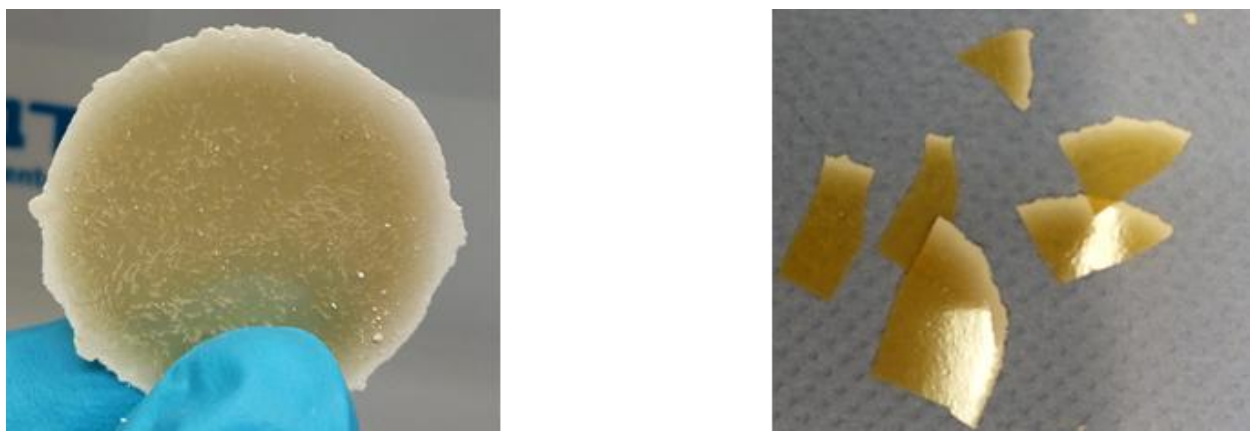

***Figure S61.*** *(a) Only brittle, brownish and opaque plates from polyoxazolidinone with DGBI as bisepoxide were obtained in a thermal press at 235 and 20 kN for 30-60 s.*

**Targeted molecular weight *versus* attained molecular weight**

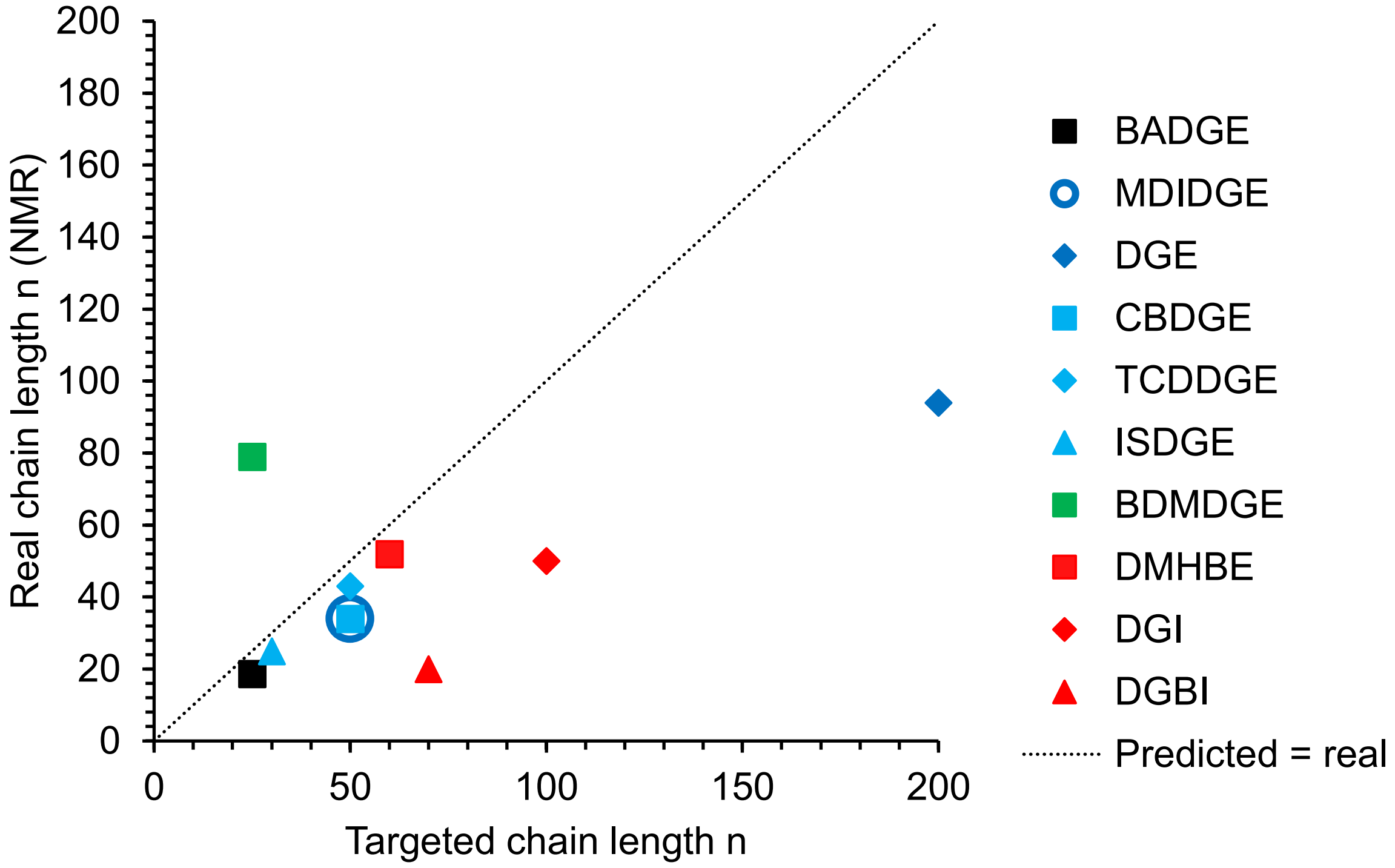


***Figure S62.*** *Actual chain length (from $^{1}$H-NMR) versus targeted chain length. The dotted line is a visual aid and connects points for which the actual* n *equals the targeted* n.

# References

[1] E. Meaurio, “Generalization of the Carothers equation for linear step growth polymers” *Sci. Rep.* **2024**, *14*, 27281.
[2] D. Schmidt, *Bisphenol a (BPA) Free Epoxy Resins*, **2012**, WO2012149340A1.
[3] S. Cassel, C. Debaig, T. Benvegnu, P. Chaimbault, M. Lafosse, D. Plusquellec, P. Rollin, “Original Synthesis of Linear, Branched and Cyclic Oligoglycerol Standards” *Eur. J. Org. Chem.* **2001**, *2001*, 875–896.
[4] R. Auvergne, S. Caillol, G. David, B. Boutevin, J.-P. Pascault, “Biobased Thermosetting Epoxy: Present and Future” *Chem. Rev.* **2014**, *114*, 1082–1115.
[5] J. Łukaszczyk, B. Janicki, A. López, K. Skołucka, H. Wojdyła, C. Persson, S. Piaskowski, M. Śmiga-Matuszowicz, “Novel injectable biomaterials for bone augmentation based on isosorbide dimethacrylic monomers” *Mater. Sci. Eng. C* **2014**, *40*, 76–84.
[6] J. Hong, D. Radojčić, M. Ionescu, Z. S. Petrović, E. Eastwood, “Advanced materials from corn: isosorbide-based epoxy resins” *Polym. Chem.* **2014**, *5*, 5360–5368.
[7] J. Deng, X. Liu, C. Li, Y. Jiang, J. Zhu, “Synthesis and properties of a bio-based epoxy resin from 2,5-furandicarboxylic acid (FDCA)” *RSC Adv.* **2015**, *5*, 15930–15939.
[8] F. Hu, J. J. La Scala, J. M. Sadler, G. R. Palmese, “Synthesis and Characterization of Thermosetting Furan-Based Epoxy Systems” *Macromolecules* **2014**, *47*, 3332–3342.
[9] W.-J. Yi, X.-C. Yu, B. Wang, J. Zhang, Q.-Y. Yu, X.-D. Zhou, X.-Q. Yu, “TACN-based oligomers with aromatic backbones for efficient nucleic acid delivery” *Chem. Commun.* **2014**, *50*, 6454–6457.
[10] D. Binningen Porret (Novartis AG), *N, N’-Diglycidyl-Bis-Hydantoinyl Compounds, Process for Their Preparation, Their Use*, **1975**, DE1670490B2.
[11] V. R. Pulgam, S. Svenson, M. A. Zhuravel (Dendritic Nanotechnologies Inc.), *Process for Converting Multifunctional Aliphatic Alcohols*, **2009**, WO2009110971A2.
[12] L. Duan, P. Zhong, J.-B. Liu, K. Liu, N. Luo, “Photocatalytic synthesis of (un)symmetrical ureas and benzimidazolones from amines and $CS_2$” *Mol. Catal.* **2024**, *564*, 114344.
